# On-surface synthesis of a doubly anti-aromatic carbon allotrope

**DOI:** 10.1038/s41586-023-06566-8

**Published:** 2023-10-25

**Authors:** Yueze Gao, Florian Albrecht, Igor Rončević, Isaac Ettedgui, Paramveer Kumar, Lorel M. Scriven, Kirsten E. Christensen, Shantanu Mishra, Luca Righetti, Max Rossmannek, Ivano Tavernelli, Harry L. Anderson, Leo Gross

**Affiliations:** 1 Department of Chemistry, Oxford University, Chemistry Research Laboratory, Oxford, UK; 2 IBM Research Europe – Zürich, Rüschlikon, Switzerland; 3 Institute of Organic Chemistry and Biochemistry of the Czech Academy of Sciences, Prague, Czechia; 4 IBM Quantum, IBM Research – Zürich, Rüschlikon, Switzerland

## Abstract

Synthetic carbon allotropes such as graphene^1^, carbon nanotubes^2^ and fullerenes^3^ have revolutionized materials science and led to new technologies. Many hypothetical carbon allotropes have been discussed^4^, but few have been studied experimentally. Recently, unconventional synthetic strategies such as dynamic covalent chemistry^5^ and on-surface synthesis^6^ have been used to create new forms of carbon, including γ-graphyne^7^, fullerene polymers^8^, biphenylene networks^9^ and cyclocarbons^10,11^. Cyclo[*N*]carbons are molecular rings consisting of *N* carbon atoms^12,13^; the three that have been reported to date (*N* = 10, 14 and 18)^10,11^ are doubly aromatic, which prompts the question: is it possible to prepare doubly anti-aromatic versions? Here we report the synthesis and characterization of an anti-aromatic carbon allotrope, cyclo[16]carbon, by using tip-induced on-surface chemistry^6^. In addition to structural information from atomic force microscopy, we probed its electronic structure by recording orbital density maps^14^ with scanning tunnelling microscopy. The observation of bond-length alternation in cyclo[16]carbon confirms its double anti-aromaticity, in concordance with theory. The simple structure of C_16_ renders it an interesting model system for studying the limits of aromaticity, and its high reactivity makes it a promising precursor to novel carbon allotropes^15^.

## Abstract (web summary)

The carbon allotrope cyclo[16]carbon has been synthesized using on-surface chemistry and characterized with scanning tunnelling microscopy and atomic force microscopy, revealing orbital densities and bond-length alternation, which show it to be doubly anti-aromatic.

## Main

Many cyclo[*N*]carbons (*N* = 6–40) have been detected in the gas phase^12,13,16^, and two examples (C_6_ and C_8_) have been trapped in solid argon and characterized by infrared spectroscopy^17,18^. Cyclo[10]carbon, cyclo[14]carbon and cyclo[18]carbon have been characterized by scanning probe microscopy of individual molecules on NaCl surfaces at low temperature^10,11,19^. Atomic force microscopy (AFM) images revealed cumulenic structures for C_10_ and C_14_ with bond-angle alternation (BAA)^11^ and a polyynic structure for C_18_ (refs. ^10,19^). Cyclo[*N*]carbons with *N* = 4*n* + 2 (where *n* is an integer), such as C_10_, C_14_ and C_18_, are expected to be doubly aromatic and to have special stability, due to their closed-shell electronic configurations, relating to the presence of in-plane and out-of-plane aromatic Hückel circuits of 4*n* + 2 π electrons^20–25^. By contrast, cyclo[4*n*]carbons have been predicted to be less stable and doubly anti-aromatic^22–26^. Here we report the first structural characterization of a cyclo[4*n*]carbon to our knowledge. C_16_ was prepared on a NaCl surface by tip-induced chemistry from a C_16_(CO)_4_Br_2_ precursor. AFM and scanning tunnelling microscopy (STM) provide insight into the geometry and electronic structure, respectively, of neutral C_16_ and anionic C_16_^–^. We find that neutral C_16_ exhibits significant bond-length alternation (BLA), which confirms its double anti-aromaticity. Our experimental results are complemented by state-of-the-art quantum mechanical calculations, as well as by methods suitable for execution on a quantum computer.

**Fig. 1:**
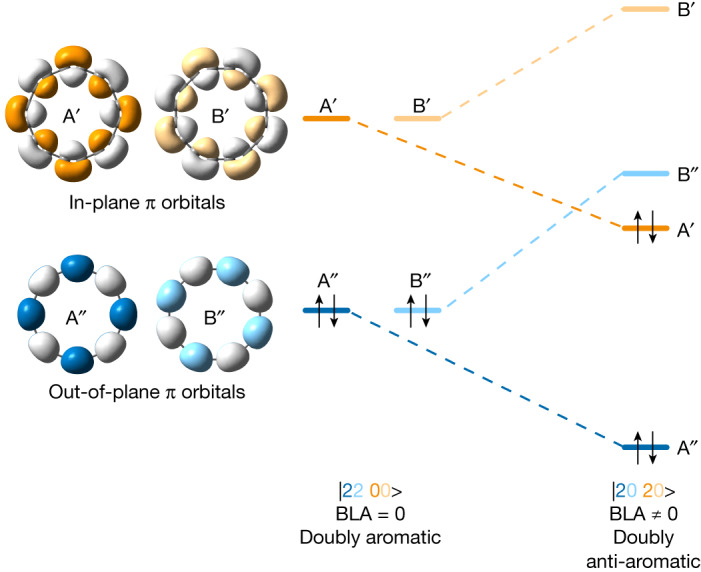
Frontier orbitals of two electronic states of C_16_. In-plane orbitals are labelled A′ and B′ and out-of-plane orbitals A′′ and B′′. Orbitals A′, B′ (and A′′, B′′) are related by rotation and have equal energy when all bonds are of equal length. Introducing BLA lifts this degeneracy, resulting in orbital reordering and a doubly anti-aromatic ground state.

Cyclocarbons have two orthogonal π systems, one with orbital lobes in the ring plane and the other out of plane, with nodes in the ring plane. In an infinitely large cyclocarbon, these two π systems are degenerate; but in a finite ring, in-plane frontier orbitals are slightly higher in energy than their out-of-plane counterparts^13^. This pattern of orbitals can lead to several possible electronic states. In the *D*_16__h_ geometry of C_16_ with no BLA, the ground state may be a doubly aromatic |2200> state (Fig. 1, left), with 18 (4*n* + 2) and 14 (4*n* − 2) electrons in out-of-plane and in-plane π systems, respectively. In this state, there are two degenerate pairs of frontier orbitals (out-of-plane A′′ and B′′ are occupied, and in-plane A′ and B′ are unoccupied). If we introduce BLA (*D*_8__h_ symmetry), these orbital pairs cease to be degenerate, with one member of each pair (A in Fig. 1, right) becoming stabilized relative to the other (B). This symmetry breaking leads to a doubly anti-aromatic |2020> configuration with 16 electrons in both in-plane and out-of-plane π systems. A third possible state would be |1111> , with *D*_16__h_ symmetry, but such open-shell configurations are known to be unstable relative to closed-shell alternatives^27^.

The unique structure, small size and high symmetry of cyclocarbons has made them a target of many theoretical studies, sometimes producing conflicting results^13^. Here, we investigate C_16_ using both state-of-the-art computational methods and a variational quantum eigensolver^28^ paired with the quantum unitary coupled-cluster singles and doubles (q-UCCSD)^29^ ansatz. These calculations confirm that the doubly anti-aromatic configuration is the ground state of C_16_, with strong BLA.

## Precursor synthesis

**Fig. 2:**
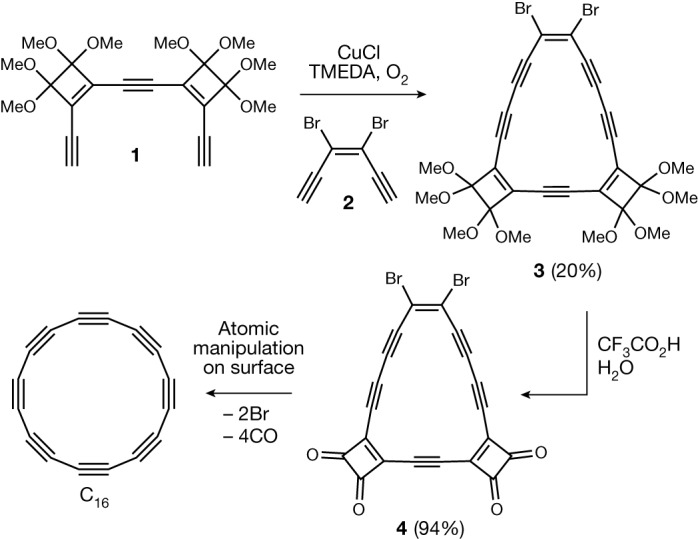
Synthesis of C_16_. TMEDA is tetramethylethylenediamine.

Cyclo[16]carbon was synthesized as shown in Fig. 2. Glaser–Hay coupling of a mixture of alkynes **1** and **2** gave macrocycle **3** in 20% yield, and the structure of this product was confirmed by single-crystal X-ray diffraction (Supplementary Fig. 3). Compounds **3** and **4** are anti-aromatic (as confirmed by the ^1^H nuclear magnetic resonance spectrum of compound **3**; Supplementary Fig. 2). Deprotection of **3** to give **4** proved difficult because of the high reactivity of compound **4**, but after testing many reaction conditions, we found that **3** can be converted to **4** in 94% yield using trifluoroacetic acid containing water (2.5% by volume).

## On-surface synthesis and characterization

**Fig. 3:**
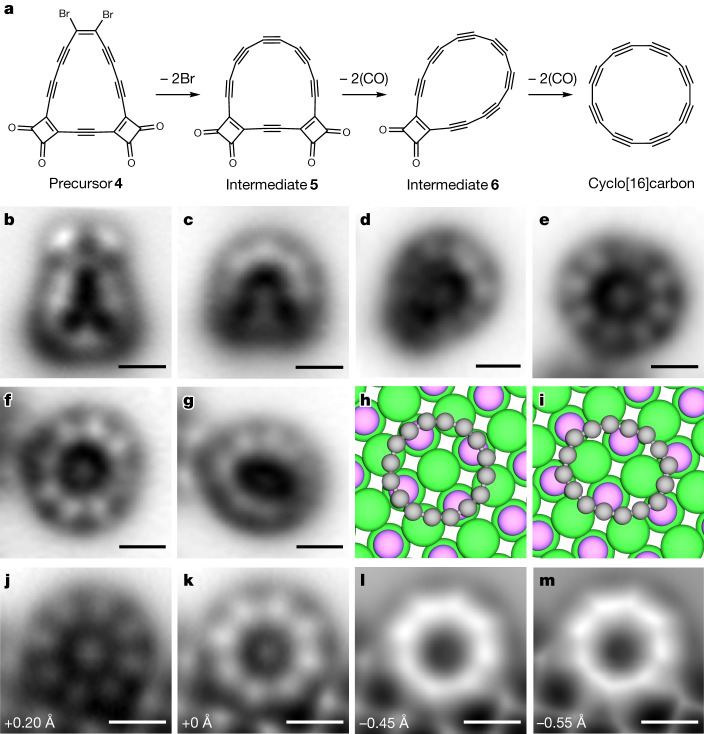
On-surface synthesis of C_16_ and structural characterization. **a**, Reaction scheme. **b**–**e**, Constant-height, CO-tip AFM images of precursor **4** (**b**), intermediates **5** (**c**) and **6** (**d**), and C_16_ (**e**). **f**–**i**, AFM image of C_16_ in neutral (**f**) and anionic (**g**) charge state, and calculated lowest-energy adsorption sites of C_16_^0^ (**h**) and C_16_^–^ (**i**) on NaCl (colour code: Na pink, Cl green). **j**–**m**, C_16_^0^ adsorbed in a bay of a third-layer NaCl island, imaged with AFM at different decreasing tip-height offsets: +0.20 Å (**j**), +0 Å (**k**), −0.45 Å (**l**) and −0.55 Å (**m**). All molecules are adsorbed on NaCl(2 ML)/Cu(111). The tip-height offsets provided in the images refer to the STM setpoint of *I* = 0.2 pA and *V* = 0.2 V on bare NaCl(2 ML)/Cu(111). Scale bars, 0.5 nm.

Precursor **4** was sublimed by fast heating from a Si wafer^10^ onto a Cu(111) single-crystal surface partially covered with NaCl at a sample temperature of about *T* = 10 K. On-surface synthesis (Fig. 3a) and characterization by STM and AFM with CO-tip functionalization^30,31^ were performed at *T* = 5 K. We found intact molecules of **4** on bilayer NaCl, denoted NaCl(2 ML)/Cu(111), as shown in Fig. 3b. The Br atoms appear as bright (repulsive) dots in the AFM image^19^, whereas the CO masking groups are dark features^10^. The triple bonds show up as bright features due to bond-order related contrast obtained with CO-tip functionalization^10,31,32^ (for further data on **4**, see Supplementary Fig. 6).

Voltage pulses applied for a few seconds at constant tip height were used to unmask the acetylenes in individual molecules of precursor **4**. We successively increased the voltage and decreased the tip height for the pulse until it resulted in dissociation reactions. For tunnelling currents on the order of few pA, the minimum voltage required for debromination of **4** to give **5** (Fig. 3c; see Supplementary Fig. 7 for further data) was 1.3 V, coinciding with the bias for resonant tunnelling: that is, electron attachment to **4** (Supplementary Fig. 6). For CO unmasking, larger bias voltages were required, typically about 3 V. We speculate that the dissociation reactions are triggered in transiently charged species by inelastic electron tunnelling processes^31^. Intermediate **6** was observed after dissociating the first pair of CO masking groups (Fig. 3d; see Supplementary Fig. 8 for further data). Removal of a second pair of CO molecules gave the final product, C_16_ (Fig. 3e and Supplementary Figs. 9 and 10). Previously, gas-phase C_16_ has been formed from a molecular precursor^33,34^ and studied in its anionic^34,35^ and cationic^16,36^ forms, but to our knowledge, this is the first time C_16_ has been generated in a condensed phase or structurally characterized. The yield for the on-surface synthesis of C_16_ was about 30%; in unsuccessful attempts, the ring opened to form linear polyynic chains (Supplementary Fig. 11) or the molecule was picked up by the tip.

**Fig. 4:**
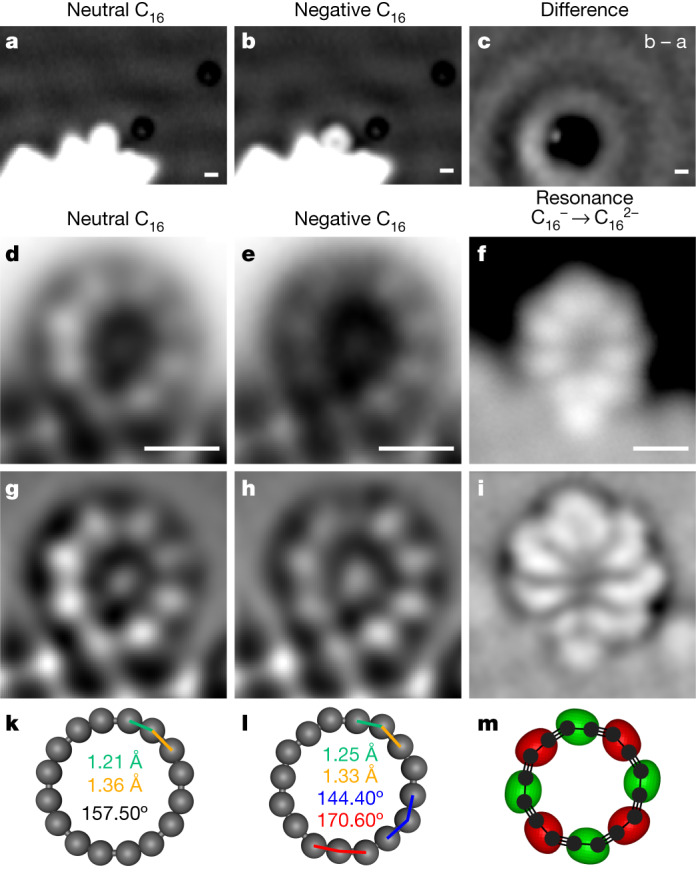
Charge-state switching and electronic characterization. **a**,**b**, Constant-current STM images of C_16_ in neutral (**a**) and negative charge state (**b**), respectively (*V* = 50 mV, *I* = 0.2 pA). **c**, Difference of panels **b** and **a**. **d**,**e**, Constant-height AFM images of C_16_^0^ (**d**) and C_16_^–^ (**e**). **f**, Constant-current STM (*I* = 0.4 pA and *V* = +1.2 V) mapping the ionic resonance of C_16_^–^ to C_16_^2–^. **g**–**i**, Same data as **d**–**f** after applying a Laplace filter. The molecule was adsorbed on NaCl(2 ML)/Cu(111) near a third-layer island. Scale bars, 0.5 nm. **k**,**l**, Optimized geometries (ωB97XD/def2-TZVP) of C_16_^0^ (**k**) and C_16_^–^ (**l**), with bond lengths and bond angles indicated. **m**, Simulated isosurface at 0.2 atomic units (1.4 e/Å^–^^3^) of the LUMO of C_16_^–^.

We observed C_16_ in two different forms on the NaCl surface (Fig. 3f,g) that we assign to neutral C_16_^0^ and negatively charged C_16_^–^, respectively (see also Fig. 4, Supplementary Figs. 12 and 13 and Supplementary Tables 1 and 2). Whereas C_16_^0^ appears circular, C_16_^–^ adopts a distorted oval geometry. We observed a variety of adsorption sites for C_16_^0^ on the NaCl surface (Supplementary Fig. 14), indicating a weak interaction with the substrate. In contrast, C_16_^–^ showed a systematic preference for adsorption above a bridge site (Supplementary Figs. 15 and 16). To investigate the interaction of C_16_^0^ and C_16_^–^ with the NaCl surface, we performed density functional theory (DFT) calculations with periodic boundary conditions, both on a pristine surface and at NaCl island step edges. The calculated lowest-energy adsorption sites of C_16_^0^ and C_16_^–^ on pristine NaCl are shown in Fig. 3h,i, respectively. For the neutral charge state, we calculated an adsorption energy of 0.65 eV, similar to the value of 0.67 eV previously calculated for C_18_ on NaCl (ref. ^37^) that was predicted to diffuse freely across the surface even at low temperatures. The calculated relaxed adsorption geometry of C_16_^–^ on pristine NaCl is oval shaped, with the molecule centred on a bridge site (Fig. 3i), in agreement with its experimentally observed site and shape (Fig. 3g, Supplementary Fig. 15 and Supplementary Table 1). This adsorption geometry can be attributed to electrostatic interactions of the C_16_^–^ anion with the Na cations and Cl anions, resulting in a substantially stronger adsorption energy (1.44 eV) than that of the neutral molecule.

The C_16_ molecules frequently moved on the surface during imaging with AFM and STM, indicating a small diffusion barrier and making them challenging to characterize. We never observed neutral C_16_ stably isolated on the NaCl surface, but always near a third-layer NaCl step edge, to provide a more stable adsorption site and facilitate detailed characterization. Figure 3j–m shows C_16_ adsorbed in a bay of a third-layer island imaged with AFM at different tip heights. Kelvin probe force spectroscopy confirmed that the molecule in Fig. 3f,j–m is charge neutral (Supplementary Tables 2 and 3 and Supplementary Fig. 17). The bright contrast obtained by CO-tip AFM above the triple bonds for larger tip heights (Fig. 3j,k) evolves to the shape of an octagon with corners at the positions of triple bonds at decreased tip heights (Fig. 3l,m). The results indicate BLA^10^: that is, a polyynic structure of neutral C_16_. Our computations (Supplementary Table 1 and Supplementary Figs. 54 and 55) predict a larger adsorption energy (1.13 and 2.61 eV for C_16_^0^ and C_16_^–^, respectively) at defect sites compared to the pristine surface, accompanied by an increase in BAA (up to 35° for C_16_^0^ and up to 50° for C_16_^–^, compared to 20–30° on a pristine surface). BLA is maintained in all cases, with no fundamental changes in the electronic structure.

## Charge-state switching

The charge state of C_16_ can be controllably switched using the applied bias, as shown in Fig. 4. At about *V* = 0.5 V, the molecule switched from neutral C_16_^0^ to the anion C_16_^–^, (and at *V* = –0.3 V in the reverse direction, C_16_^–^ to C_16_^0^; Supplementary Figs. 12 and 18). The STM images in Fig. 4a,b show C_16_^0^ and C_16_^–^, respectively. The negative charge state leads to a characteristic dark halo (Fig. 4b) and interface state scattering as observed in the difference image Fig. 4c (ref. ^38^); see also Supplementary Fig. 12 for images with enhanced contrast. The assignments of these charge states are corroborated by Kelvin probe force spectroscopy (Supplementary Fig. 18). AFM data for C_16_^0^ and C_16_^–^ are shown in Fig. 4d,e with corresponding Laplace-filtered data in Fig. 4g,h, respectively. In this case, the structural distortion of C_16_^0^ and C_16_^–^ is similar, which we assign to the influence of the third-layer NaCl island (Supplementary Table 1).

The more stable adsorption at the third-layer island allowed us to image the molecule at increased bias voltages without inducing movement of the molecule. At about 1.2 V, we observe the onset of an electronic resonance by scanning tunnelling spectroscopy (Supplementary Fig. 18). The STM image at 1.2 V shown in Fig. 4f (Laplace-filtered data in Fig. 4i), reveals the orbital density corresponding to that resonance^14^. As the molecule is already in the anionic charge state at *V* > 0.5 V, we assign this resonance to the transition from anionic C_16_^–^ to the dianionic charge state C_16_^2–^, giving us insight into the electronic structure of C_16_.

Multireference methods and DFT (see Supplementary Tables 4 and 5 for details) both predict C_16_^0^ to have a |2020> ground state with a polyynic geometry and BLA, but no BAA, in the gas phase (*D*_8__h_ symmetry, Fig. 4k). The electronic structure of C_16_^0^ (Fig. 1, right, and Supplementary Fig. 19) features a nearly degenerate pair of highest occupied molecular orbitals A′′ (HOMO–1) and A′ (HOMO), as well a nearly degenerate pair of lowest unoccupied molecular orbitals B′′ (LUMO) and B′ (LUMO + 1). A′ and B′ (as well as A′′ and B′′) are related by rotation; in a magnetic field, they couple to induce a strong ring current (–25 nA T^–1^, cf. 12 nA T^–1^ in benzene), reinforcing the applied field inside the ring. This current is a signature of anti-aromaticity^39^, and it can be visualized by nucleus-independent chemical shift calculations (see comparison of the plots for the |2200> and |2020> states of C_16_ in Supplementary Fig. 28). In contrast to the neutral |2020> state of C_16_, the anion shows both BLA and BAA (Fig. 4l), due to single occupation of the B′′ orbital, resulting in *C*_8__h_ symmetry.

The DFT-predicted LUMO of C_16_^–^ (Fig. 4m and Supplementary Fig. 20) can be compared to the electronic resonance imaged by STM (Fig. 4f,i), which corresponds to the squared orbital wavefunction^14,38^, and to the addition of a second electron to the singly occupied out-of-plane orbital (B′′) in C_16_^–^. Both theory and experiment show high-density lobes above the long bonds of C_16_^–^, which are located between the bright features of the corresponding AFM images. The symmetry lowering from *D*_8__h_ to *C*_8__h_ in C_16_^–^, which is the effect of BAA, is reflected in the shape of the orbital lobes and can be observed in both experiment (Fig. 4f,i) and theory (Fig. 4m). AFM data showing BLA, and STM data showing the orbital density for the C_16_^–^ to C_16_^2–^ transition, corresponding to the addition of an electron to the B′′ orbital of C_16_^–^, are all in excellent agreement with the calculations, strongly indicating the doubly anti-aromatic character of C_16_^0^, which causes pronounced BLA and a *D*_8__h_ geometry. The two other possible electronic configurations of C_16_, doubly aromatic |2200> and open-shell |1111>, were calculated by DFT to have nearly identical *D*_16__h_ minima with no BLA and substantially higher energies (2.47 and 1.78 eV, respectively) than the doubly anti-aromatic |2020> ground state. Relative ground-state energies of the *D*_8__h_ and *D*_16__h_ minima were also determined using q-UCCSD by simulating quantum circuits with Qiskit^40^. q-UCCSD predicts that the *D*_8__h_ minimum is more stable than the *D*_16__h_ minimum by 3.38 eV, which is very similar to the result obtained using conventional coupled-cluster singles and doubles (3.31 eV; see Supplementary Information for further discussion).

Our experimental results, most importantly the observed BLA for neutral C_16_, confirm the occupation of both π systems (in-plane and out-of-plane) with 16 electrons, making the molecule doubly anti-aromatic. Ring current calculations on neutral C_16_ also indicate significant anti-aromaticity in this electronic configuration. The investigation of both C_16_^0^ and C_16_^–^ provides confidence in the assignment of charge states and insights into the electronic structure of the molecule. The synthesis, stabilization and characterization of C_16_ opens the way to create other elusive carbon-rich anti-aromatic molecules by atom manipulation.

## Online content

Any methods, additional references, Nature Portfolio reporting summaries, source data, extended data, supplementary information, acknowledgements, peer review information; details of author contributions and competing interests; and statements of data and code availability are available at 10.1038/s41586-023-06566-8.

### Supplementary information

Supplementary Information Supplementary experimental and computational methods, figures and data.

Peer Review File

## Data Availability

The data that support the findings of this study are available in the paper and its Supplementary Information, or are available from the Zenodo public repository (https://zenodo.org/record/8226451 and 10.5281/zenodo.8226451). Crystallographic data for compound **3** are available free of charge from the Cambridge Crystallographic Data Centre (CCDC 2240722), https://www.ccdc.cam.ac.uk/data_request/cif.
