# Supplementary material for: On-surface synthesis of a doubly anti-aromatic carbon allotrope
Source: Nature. 2023 Oct 25;623(7989):977–81. doi: 10.1038/s41586-023-06566-8 (PMC10686826; doi:10.1038/s41586-023-06566-8)
Supplement: Supplementary file 1 — Supplementary experimental and computational methods, figures and data. [file 41586_2023_6566_MOESM1_ESM.pdf]

nature portfolio

https://doi.org/10.1038/s41586-023-06566-8

Supplementary information

On-surface synthesis of a doubly anti-aromatic carbon allotrope

In the format provided by the authors and unedited

Supplementary Information for

On-Surface Synthesis of a Doubly Anti-Aromatic Carbon Allotrope

Yueze Gao,[1]† Florian Albrecht,[2]†, Igor Rončević,[1,4] Isaac Ettedgui,[1] Paramveer Kumar,[1] Lorel M. Scriven,[1] Kirsten E. Christensen,[1] Shantanu Mishra,[2] Luca Righetti,[3] Max Rossmannek,[3] Ivano Tavernelli,[3] Harry L. Anderson[1]* and Leo Gross[2]*

[1] Department of Chemistry, Oxford University, Chemistry Research Laboratory, Oxford, United Kingdom.

[2] IBM Research Europe – Zürich, 8803 Rüschlikon, Switzerland.

[3] IBM Quantum, IBM Research – Zürich, 8803 Rüschlikon, Switzerland.

[4] Institute of Organic Chemistry and Biochemistry of the Czech Academy of Sciences, Prague, Czechia.

† These authors contributed equally.

*Corresponding authors. Email: harry.anderson@chem.ox.ac.uk; lgr@zurich.ibm.com

Table of Contents

Section A. Materials and Methods **2**
1) Experimental STM and AFM setup 2
2) Synthetic general methods 2
3) Computational methods 2
4) Synthetic protocols 4
5) Comparison of ^{1}H NMR spectra 12
6) Stability tests 12
7) Additional STM and AFM data 13
8) Computational results (gas phase) 13
9) Computational results (surface adsorption) 14

Section B. Supplementary Figures **15**

Section C. Supplementary Tables **54**

***Section D.* List of Deposited Datasets** **59**

***Section E.* Supplementary References** **60**

A. Materials and Methods

A1. Experimental STM and AFM setup:

The on-surface characterization and reactions were performed in a home-built combined scanning tunnelling and atomic force microscope, operated at a temperature of 5 Kelvin in UHV. Molecules of **4** where thermally sublimed onto a cold (T < 10 K) Cu(111) surface partially covered with NaCl islands of two and three atomic layers thickness. AFM measurements were performed in non-contact mode with a qPlus sensor[1]. The sensor was operated in frequency modulation mode[2] with the oscillation amplitude kept constant at 0.5 Å. If not noted otherwise, all data were recorded on molecules adsorbed on bilayer NaCl with CO functionalized tips. STM images were recorded at constant current and AFM images at constant height. The STM controlled setpoint for constant-height AFM images was I = 0.2 pA and V = 0.2 V unless stated otherwise. Positive (negative) tip-height offsets correspond to an increase (decrease) in tip-sample distance with respect to the setpoint on bare bilayer NaCl. AFM images were acquired at V = 0 V.

A2. Synthetic general methods:

Reagents were purchased reagent-grade from commercial suppliers and used without further purification. Dry solvents (toluene, Et_2O, THF, $CHCl_3$) for reactions were purified by a MBraun MB-SPS-5 bench-top SPS system under nitrogen. All other solvents used were HPLC grade. $MgSO_4$ was used as the drying reagent after the aqueous work-up. Petroleum ether has a boiling point range of 40–60 °C. Thin layer chromatography (TLC) was carried out on aluminum-backed silica gel plates with 0.2 mm thick silica gel 60 F254 (Merck) and visualized via UV-light (254/364 nm). Flash column chromatography was either carried out using flash silica gel 60 (230-400 mesh) obtained from Sigma-Aldrich, or on a Biotage Isolera One with a 200–400 nm UV detector. ^{1}H and ^{13}C NMR spectra were recorded on Bruker AVIII HD 400 spectrometers at 400 MHz (^{1}H) and 101 MHz (^{13}C) and Bruker AVIII HD 500 spectrometers at 500 MHz (^{1}H) and 126 MHz (^{13}C), respectively, at 298 K unless stated otherwise. NMR chemical shifts are reported in ppm relative to $SiMe_4$ (δ = 0) and were referenced internally with respect to residual solvent protons using the reported values (^{1}H: $CDCl_3$: 7.26 ppm; ^{13}C: $CDCl_3$: 77.0 ppm). All chemical shifts are reported in ppm, coupling constants are reported in Hz and ^{1}H multiplicities are reported in accordance with the following: s = singlet; d = doublet; t = triplet; and m = multiplet. High-resolution mass spectrometry measurements were carried out by electrospray ionization (ESI) mass spectrometry on a Thermo Scientific Q Exactive Hybrid Quadrupole-Orbitrap mass spectrometer by the mass spectrometry service at the University of Oxford. UV-vis spectra were recorded in solution on a Perkin-Elmer Lambda 20 spectrometer at 21 °C (unless otherwise noted), in fused silica cuvettes with a path length of 1 cm. IR spectra were recorded as a thin film on a Bruker Tensor 27 spectrometer equipped with a Diamond ATR sample compartment.

Single crystal X-ray diffraction data for compound **3** were collected at 150 K using a (Rigaku) Oxford Diffraction SuperNova diffractometer and CrysAlisPro. Structure was solved using 'Superflip'[3] before refinement with CRYSTALS[4,5] as per the SI (CIF). The crystallographic data have been deposited with the Cambridge Crystallographic Data Center (CCDC 2240722), and copies of these data can be obtained free of charge from the Cambridge Crystallographic Data Center via www.ccdc.cam.ac.uk/data_request/cif.

A3. Computational methods:

CASSCF, CASPT2, and NEVPT2 calculations were done using a 12,12 active space which included 6 in-plane and 6 out-of-plane orbitals (shown on Fig. S19). The geometry optimization at the NEVPT2 level was done using a grid search with a convergence criterion of 5 meV, assuming D_{8h} symmetry (see Fig. S23). EOM-CCSD calculations were done on the NEVPT2-optimized geometry and included 6 roots. Single point NEVPT2 and CASPT2 calculations used a state-averaged wavefunction with two states; increasing the number of states to four resulted in changes of less than

30 meV. CASSCF, NEVPT2, and CCSD calculations were done using ORCA[6], while CASPT2 and Dyson orbital calculations were done with OpenMolcas[7]. Canonical CCSD calculations suffered from convergence issues, so a localized variant based on domain-based local pair natural orbitals (DLPNO) was used instead. In the DLPNO approach[8], more than 95% of local pairs were explicitly correlated, leading to results very close to canonical CCSD. DFT calculations were done using Gaussian16 (ref. 9). All optimizations were carried out using the def2-TZVP basis; single-point calculations done using Gaussian and ORCA utilized the def2-QZVPP basis set[10]; calculations done in OpenMolcas used ANO-L-VTZP[11].

Adsorption of C_{16} on a pristine (100) NaCl surface, as well as at several geometries of NaCl island step-edges, was modelled using the PBE[12] density functional with D3BJ[13] corrections for long-range interactions. Calculations were performed using the VASP code[14,15], utilizing standard PAW pseudopotentials with a kinetic energy cutoff of 400 eV. To obtain a surface slab suitable for adsorbing C_{16}, a 5-layer gamma-sampled 6×6 supercell of a (100) NaCl surface with ~24 Å of vacuum in the *z* direction was reoptimized, with the lattice parameter fixed at 2.72 Å[16]. Optimizations of neutral and negatively C_{16} adsorbed on the surface (C_{16}@NaCl) and different NaCl island step-edges (see Table S1 and Fig. S54) were done by placing the C_{16} molecule in different arrangements and performing a relaxation in which the total energy was converged within 10^{-5} eV. Adsorption energy was calculated as the negative difference between the optimized geometry and a reference, which was obtained by placing a gas-phase optimized molecule of C_{16} in the center of a unit cell with the reconstructed NaCl (Fig. S24). The use of such a reference is required for calculating the adsorption energy of the C_{16} anion, as the total energy of $[C_{16}@NaCl]^-$ is affected by compensating background charges, making a direct comparison with the separated (neutral) surface slab and gas-phase C_{16}^- unphysical. For neutral C_{16}, we note that this method gives an adsorption energy 3.3 meV/atom smaller than in the case when using separated gas-phase C_{16} and the reconstructed NaCl surface as a reference.

We also employed the recently introduced quantum unitary coupled clusters singles doubles (q-UCCSD) method[17]. q-UCCSD is based on a modification of the coupled clusters algorithm in which the excitation amplitudes are mapped onto quantum circuits. q-UCCSD calculations were done using fixed orbitals obtained at the ωB97XD/def2-SVP level of theory and allowed single and double excitations in a (6,8) active space. Calculations on the optimized D_{8h} and D_{16h} geometries of C_{16} produced a result very similar to conventional DLPNO-CCSD, demonstrating that q-UCCSD can approach the accuracy of state-of-the-art wavefunction theory methods despite using a much smaller basis (912 basis functions for CCSD vs 224 for q-UCCSD) and a limited active space.

A4. Synthetic protocols:

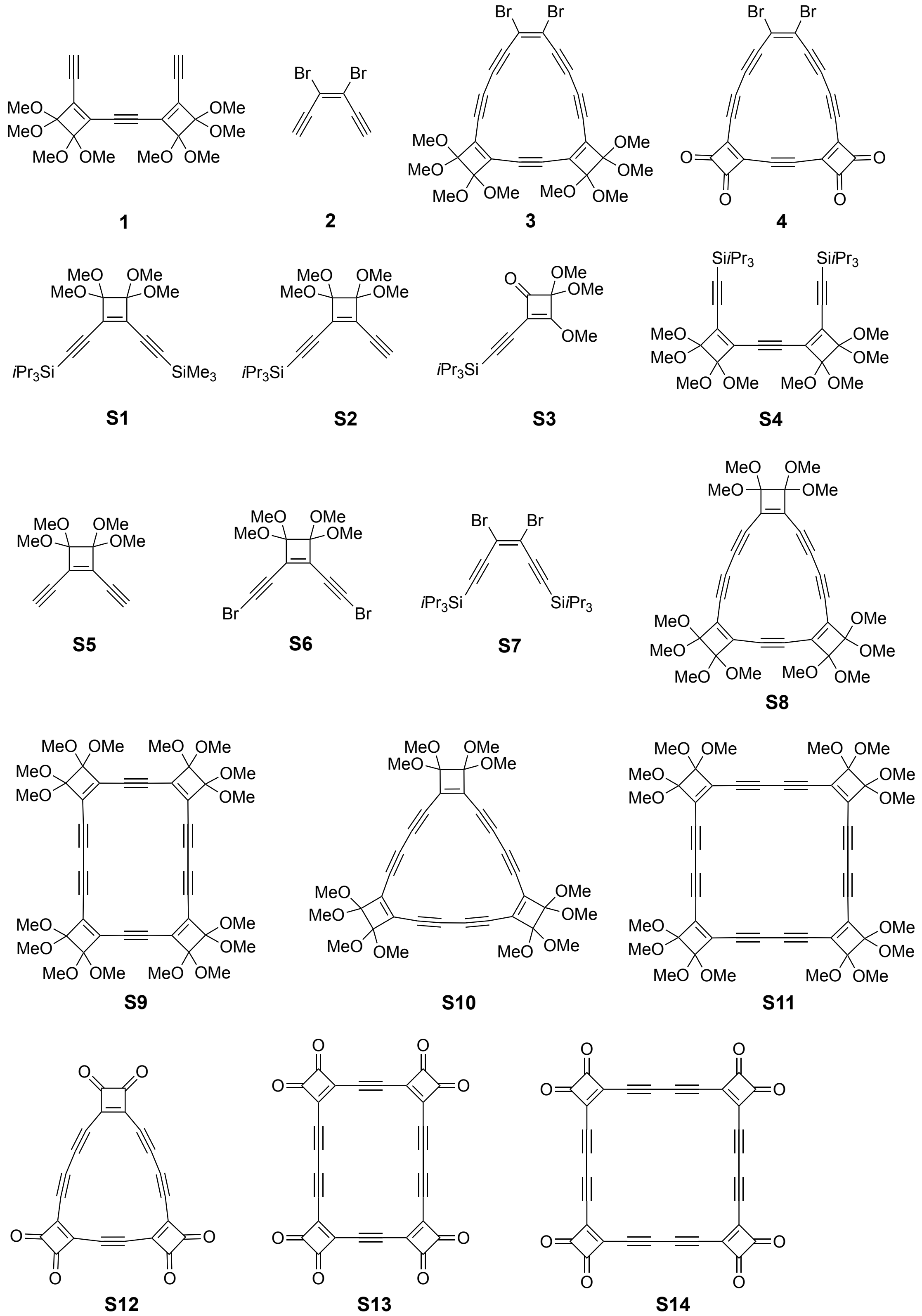

Fig. S1. Molecular structures discussed in the paper.

Compounds **2**, **S1**, **S3**, **S7**, and **S10** were synthesized as described previously[18,19].

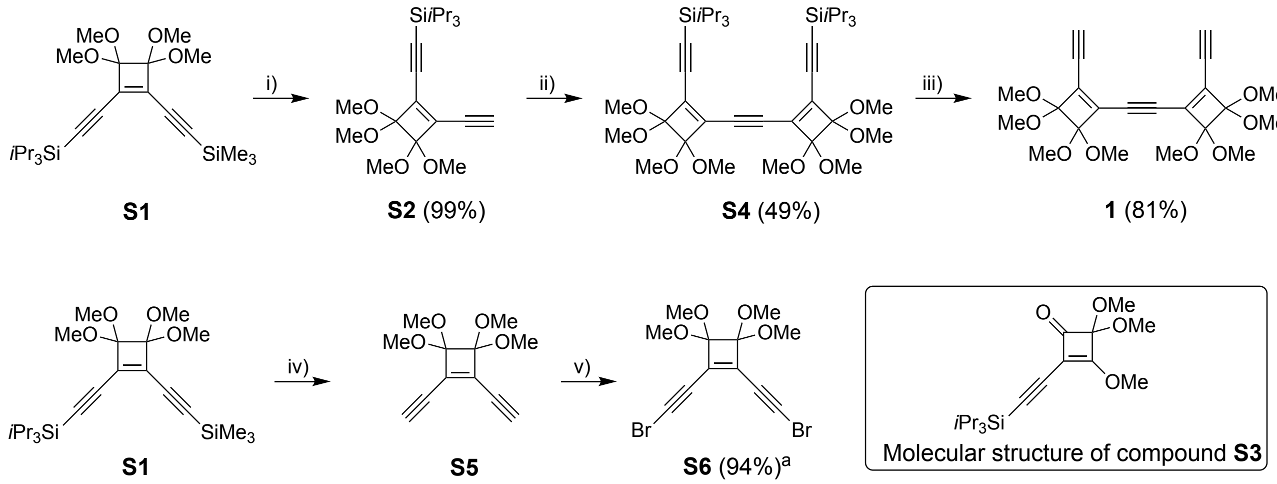

Scheme S1. a) Synthesis of building blocks of **1**, **S5**, and **S6**. Reagents and conditions: i) K_2CO_3, THF/MeOH (1:1), 25 °C. ii) a) *n*-BuLi (1.6 M in hexane), Et_2O, –78 °C; b) compound **S3**, –78 to 0 °C; c) HCl solution (3.0 M in water), 25 °C; d) $SiMe_3OTf$, $SiMe_3OMe$, 25 °C. iii) TBAF (1.0 M in THF), wet THF, 25 °C. iv) TBAF (1.0 M in THF), wet THF, 0 °C. v) NBS, acetone, 25 °C. [a]The yield of compound **S6** is over two steps from compound **S1**. The synthesis of compound **S1**, **S3**, and **S5** has been reported previously[18]. TBAF = tetra-*n*-butylammonium fluoride; NBS = *N*-bromosuccinimide.

General procedure for synthesis of compound S5: The formation of compound **S5** was adapted of a reported procedure[18]. Unless otherwise noted in the individual procedures, to a solution of the compound **S1** (300 mg, 0.666 mmol) in THF (10 mL) and H_2O (0.10 mL) was added tetrabutylammonium fluoride (TBAF, 1.0 M in THF, 1.46 mmol) at 0 °C. The solution was stirred for 1 h at 0 °C. Water (10 mL) and petroleum ether (20 mL) were then added, the layers were separated, and the aqueous phase was extracted with petroleum ether (2 × 10 mL). The solution was concentrated to ca. 2 mL in *vacuo*. Purification by passing through a silica plug (ethyl acetate/petroleum ether 1:4) resulted in a solution containing compound **S5**, which was then concentrated to ca. 2 mL. This resulting solution was used immediately in the next step.

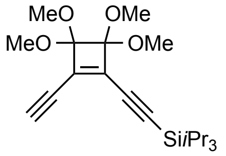

Compound S2: To a solution of compound **S1** (2.00 g, 4.44 mmol) in THF (30 mL) and methanol (30 mL) was added K_2CO_3 (0.920 g, 6.66 mmol) at 25 °C, and this solution was stirred for 30 min. H_2O (30 mL) and CH_2Cl_2 (50 mL) were added, the layers were separated, and the aqueous phase was extracted with CH_2Cl_2 (2 × 20 mL). The organic phases were combined, washed with brine (20 mL), dried ($MgSO_4$), and filtered. Solvent removal and purification by column chromatography (silica gel, petroleum ether/CH_2Cl_2 1:1) afforded compound **S2** (1.67 g, 99%) as a yellow oil.
IR (ATR): 624, 661, 678, 715, 821, 883, 904, 984, 1038, 1080, 1179, 1203, 1257, 1367, 1384, 1463, 2094, 2137, 2361, 2837, 2866, 2894, 2943, 3249 (broad) cm^{-1}. **^{1}H NMR** (400 MHz, $CDCl_3$): δ 3.67 (s, 1H; CCH), 3.52 (s, 6H; OCH_3), 3.51 (s, 6H; OCH_3), 1.08–1.07 (m, 21H; *i*Pr_3). **^{13}C NMR** (101 MHz): δ 137.2, 134.5, 108.1, 107.9, 107.8, 97.5, 90.4, 75.2, 52.0, 51.9, 18.5, 11.1. **ESI HRMS *m/z*:** calcd for $C_{21}H_{34}O_4NaSi^+$ ($[M + Na]^+$) 401.2119, found 401.2123.

Compound S4: *n*-BuLi (3.01 mL, 1.6 M in hexanes, 4.81 mmol) was added to a solution of compound **S2** (1.69 g, 4.46 mmol) in Et_2O (20 mL) at –78 °C. The solution was stirred at –78 °C for 1 h. A solution of compound **S3** (1.48 g, 4.37 mmol) in Et_2O (20 mL) was added at –78 °C, and this resulting solution was stirred for 10 min. The cooling bath was removed, and the solution was stirred at 0 °C for 1 h. HCl solution (3.0 M in H_2O, 20 mL) was then added, and the mixture stirred vigorously for 1 h. H_2O (30 mL) was then added, the layers were separated, and the aqueous phase was extracted with Et_2O (2 × 10 mL). The organic phases were combined, washed with sat. $NaHCO_3$ (20 mL), and the layers were separated. The resultant organic phase was collected and dried over $MgSO_4$. Solvent removal gave a crude product, which was carried on to the next step without further purification. To the solution of the crude product in methoxytrimethylsilane (TMSOMe, 15 mL) was added trimethylsilyl trifluoromethanesulfonate (TMSOTf, 0.51 mL, 2.85 mmol) over 10 min under a N_2 atmosphere, and this solution was stirred for 88 h. Sat. aqueous solution of $NaHCO_3$ (20 mL) and Et_2O (30 mL) were added, the layers were separated, and the aqueous phase was extracted with Et_2O (2 × 20 mL). The organic phases were combined, washed with brine (20 mL), dried ($MgSO_4$), and filtered. Solvent removal and purification by column chromatography (silica gel, petroleum ether/ethyl acetate 100:1 to 10:1) afforded compound **S4** (1.57 g, 49%) as an off-white solid.

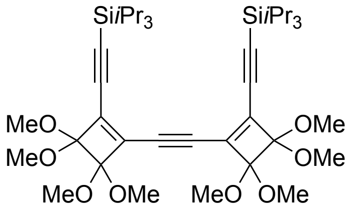

Mp 107–108 °C. **IR (ATR):** 621, 675, 719, 883, 941, 985, 1041, 1080, 1180, 1255, 1341, 1463, 2133, 2367, 2836, 2866, 2943 cm^{-1}. **^{1}H NMR** (500 MHz, $CDCl_3$): δ 3.52 (s, 12H; OCH_3), 3.48 (s, 12H; OCH_3), 1.07–1.05 (m, 42H; *i*Pr_3). **^{13}C NMR** (126 MHz, $CDCl_3$): δ 136.4, 133.8, 108.9, 108.3, 108.0, 97.9, 93.1, 52.04, 52.01, 18.5, 11.1. **ESI HRMS *m/z*:** calcd for $C_{40}H_{66}O_8NaSi_2^+$ ($[M + Na]^+$) 753.4188, found 753.4185.

Compound 1: To a solution of compound **S4** (260 mg, 0.356 mmol) in THF/H_2O (25 mL/0.2 mL) was added tetrabutylammonium fluoride (TBAF, 1.0 M in THF, 0.78 mL, 0.78 mmol) at 25 °C, and the solution was stirred for 30 min. H_2O (20 mL) and CH_2Cl_2 (30 mL) were added, the layers were separated, and the aqueous phase was extracted with CH_2Cl_2 (2 × 20 mL). The organic phases were combined, washed with brine (20 mL), dried ($MgSO_4$), and filtered. Solvent removal and purification by column chromatography (silica gel, petroleum ether/ethyl acetate 10:1) afforded compound **1** (121 mg, 81%) as an off-white solid.

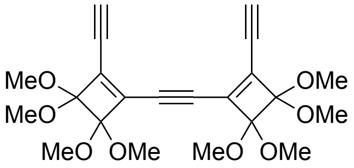

Mp 135 °C (decomp). **IR (ATR):** 671, 872, 933, 983, 1040, 1079, 1180, 1195, 1253, 1333, 1457, 1457, 2093, 2360, 2360, 2838, 2943, 3238 cm^{-1}. **^{1}H NMR** (500 MHz, $CDCl_3$): δ 3.76 (s, 2H, CCH), 3.51 (s, 12H; OCH_3), 3.49 (s, 12H; OCH_3). **^{13}C NMR** (126 MHz, $CDCl_3$): δ 136.1, 135.4, 108.4, 107.9, 92.8, 92.1, 75.1, 52.1, 52.0. **ESI HRMS *m/z*:** calcd for $C_{22}H_{27}O_8^+$ ($[M + H]^+$) 419.1700, found 419.1692.

Compound S6: Compound **S1** (215 mg, 0.477 mmol) in THF/H_2O (10 mL/0.1 mL) was subjected to desilylation according to the **general procedure** using TBAF (1.0 M in THF, 1.05 mL, 1.05 mmol). To this resulting solution containing compound **S5** in acetone (10 mL) was added *N*-bromosuccinimide (NBS, 212 mg, 1.19 mmol) and $AgNO_3$ (16 mg, 0.095 mmol). The solution was stirred for 1 h at 25 °C, with the flask wrapped in aluminum foil to avoid exposure to light. H_2O (20 mL) and CH_2Cl_2 (30 mL) were added, the layers were separated, and the aqueous phase was extracted with CH_2Cl_2 (2 × 20 mL). The organic phases were combined, washed with brine (20 mL), dried ($MgSO_4$), and filtered. Solvent removal and purification by column chromatography (silica gel, petroleum ether/ CH_2Cl_2 1:1) afforded compound **S6** (170 mg, 94%) as a yellowish oil. Dissolving this compound in petroleum ether and standing the solution in a freezer gave a white crystalline solid.

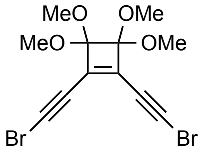

^{1}H NMR (500 MHz, $CDCl_3$): δ 3.48 (s, 12H, OCH_3). **^{13}C NMR** (126 MHz, $CDCl_3$): δ 135.8, 108.1, 72.2, 65.2, 52.0. **ESI HRMS *m/z*:** calcd for $C_{12}H_{12}Br_2O_4Na^+$ ($[M + Na]^+$) 402.8974, found 402.8973.

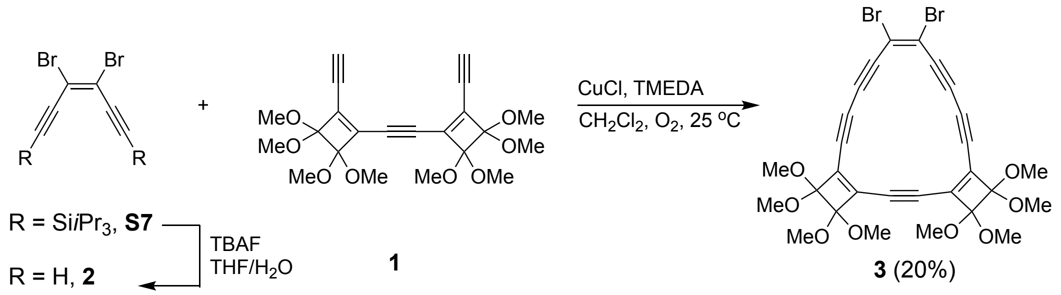

Compound 3: To a solution of compound **S7** (60 mg, 0.11 mmol) in THF/H_2O (3.0 mL/0.030 mL) was added tetrabutylammonium fluoride (TBAF, 1.0 M in THF, 0.27 mL, 0.27 mmol) at 0 °C, and the solution was stirred for 30 min. H_2O (10 mL) and CH_2Cl_2 (20 mL) were added, the layers were separated, and the aqueous phase was extracted with CH_2Cl_2 (2 × 10 mL). The organic phases were combined, washed with brine (20 mL), dried ($MgSO_4$), and filtered. The solution was concentrated to ca. 5 mL and went through a short silica plug (CH_2Cl_2). This resulting solution was concentrated to ca. 25 mL. To this resulting solution containing compound **2** in CH_2Cl_2 (25 mL) was added compound **1** (40 mg, 0.096 mmol). Then the Glaser-Hay catalyst was prepared by adding CuCl (19 mg, 0.019 mmol) to a solution of TMEDA (89 mg, 0.77 mmol) in CH_2Cl_2 (300 mL), and the solution was stirred for 5 min at 25 °C. To this resulting solution containing Glaser-Hay catalyst was added the solution of compound **1** and **2** in CH_2Cl_2 over 4 h via a syringe pump. Then the mixture was stirred for another 18 h. H_2O (50 mL) was added, the layers were separated, and the aqueous phase was extracted with CH_2Cl_2 (2 × 20 mL). The organic phases were combined, washed with brine (20 mL), dried ($MgSO_4$), and filtered. Solvent removal and purification by column chromatography (silica gel, ethyl acetate/CH_2Cl_2/petroleum ether 1:5:30 to 1:5:10) afforded compound **3** (12.5 mg, 20%) as a wine-red solid. A crystal of **compound 3** suitable for X-ray crystallographic analysis was grown at room temperature, by slow evaporation from a CH_2Cl_2 solution layered with MeOH.
R_f = 0.83 (ethyl acetate/petroleum ether 1:1). **IR (ATR):** 2936 (br), 2836 (w), 2184 (w), 2121 (w), 1251 (m), 1083 (s) cm^{-1}. **UV/Vis** ($CHCl_3$) λ_{max} (ε): 274 (24300), 291 (34400), 307 (41400), 337 (sh, 6140), 361 (sh, 3470), 389 (1500), 433 (707), 474 (593), 511 (542), 568 (318). **^{1}H NMR** (500 MHz, $CDCl_3$) δ 3.26 (s, 12H; OCH_3), 3.24 (s, 12H; OCH_3). **^{13}C NMR** (126 MHz, $CDCl_3$) δ 142.5, 139.8, 116.3, 107.6, 107.5, 96.6, 88.8, 86.6, 84.2, 80.3, 52.0, 51.8. **ESI HRMS *m/z*:** calcd for $C_{28}H_{24}{}^{79}Br^{81}BrO_8Na^+$ ($[M + Na]^+$) 670.9711, found 670.9705.

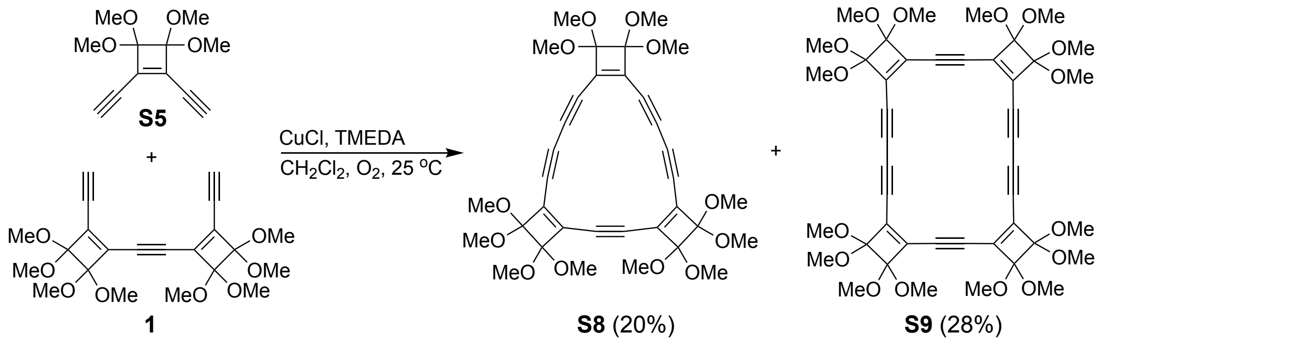

Compound S8: (Glaser-Hay Method) Compound **S1** (40 mg, 0.089 mmol) in THF/H_2O (4 mL/0.05 mL) was subjected to desilylation according to the **general procedure** using TBAF (1.0 M in THF, 0.195 mL, 0.195 mmol). To this resulting solution containing compound **S5** in CH_2Cl_2 (25 mL) was added compound **1** (30 mg, 0.072 mmol). Then the Glaser-Hay catalyst was prepared by adding CuCl (7.1 mg, 0.072 mmol) to a solution of TMEDA (41.7 mg, 0.358 mmol) in CH_2Cl_2 (200 mL), and the solution was stirred for 5 min at 25 °C. To this resulting solution containing Glaser-Hay catalyst was added the solution of compound **1** and **S5** in CH_2Cl_2 over 3.5 h via a syringe pump. Then the mixture was stirred for another 16 h. H_2O (50 mL) was added, the layers were separated, and the aqueous phase was extracted with CH_2Cl_2 (2 × 20 mL). The organic phases were combined, washed with brine (20 mL), dried ($MgSO_4$), and filtered. Solvent removal and purification by column chromatography (silica gel, ethyl acetate/CH_2Cl_2/petroleum ether 1:5:30 to 1:5:10) afforded compound **S8** (9 mg, 20%) as a pink-red solid and compound **S9** (8.5 mg, 28%) as a red solid.

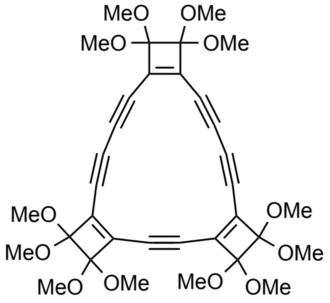

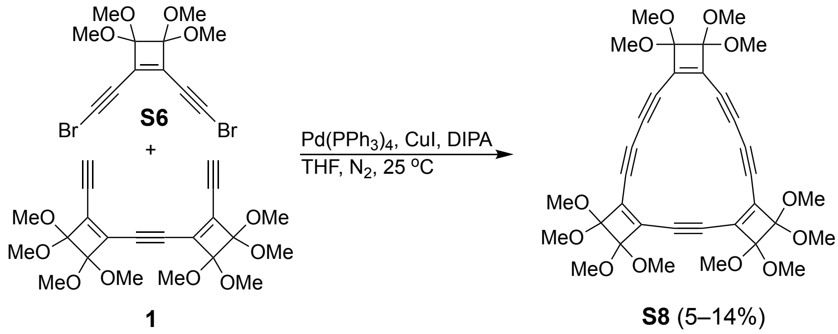

Pd-assisted Cadiot-Chodkiewicz Method: Compound **1** (25 mg, 0.060 mmol) and $Pd(PPh_3)_4$ (5.5 mg, 0.0048 mmol) were added to a solution of compound **S6** (27 mg, 0.72 mmol) in THF (25 mL), and the solution was deoxygenated under a flow of N_2 for 30 min. A solution of CuI (22.8 mg, 0.119 mmol) and $Pd(PPh_3)_4$ (15.2 mg, 0.013 mmol) in THF (35 mL) was deoxygenated under a flow of N_2 for 30 min, and a freshly deoxygenated diisopropylamine (DIPA, 0.84 mL, 6.0 mmol) was added. Then to this resulting solution containing CuI, $Pd(PPh_3)_4$, and DIPA was added the solution containing compound **1**, compound **S6**, and $Pd(PPh_3)_4$ in THF over 3 h, and the solution was stirred for another 20 h at 25 °C. H_2O (50 mL) was added, the layers were separated, and the aqueous phase was extracted with CH_2Cl_2 (2 × 20 mL). The organic phases were combined, washed with brine (20 mL), dried ($MgSO_4$), and filtered. Solvent removal and purification by column chromatography (silica gel, ethyl acetate/CH_2Cl_2/petroleum ether 1:5:30 to 1:5:10) afforded compound **S8** (3 mg, 8%) as a pink-red solid. (Note that the yield varied 5–14%.)

R_f = 0.73 (ethyl acetate/petroleum ether 1:1). **IR (ATR):** 2941 (br), 2837 (w), 1726 (w), 1254 (m), 1083 (s) cm^{-1}. **UV/Vis** ($CHCl_3$) λ_{max} (ε): 269 (21100), 285 (36700), 299 (sh, 34700), 303 (37000), 341 (sh, 4800), 360 (3020), 394 (1000), 401 (984), 454 (sh, 555), 510 (482), 564 (269). **^{1}H NMR** (500 MHz, $CDCl_3$) δ 3.23 (s, 24H; OCH_3), 3.22 (s, 12H; OCH_3). **^{13}C NMR** (126 MHz, $CDCl_3$) δ

146.7, 143.1, 139.5, 107.7, 107.3, 107.1, 96.9, 93.1, 88.8, 83.2, 82.1, 52.0, 51.9, 51.8. **ESI HRMS *m/z*:** calcd for $C_{34}H_{36}O_{12}Na^+$ ($[M + Na]^+$) 659.2099, found 659.2093.

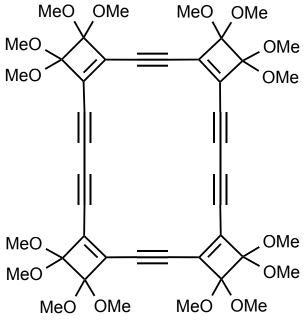

Compound S9: The titled compound **S9** (8.5 mg, 28%) was obtained as a red solid using the procedure described above: **Glaser-Hay Method**.
***R**$_f$* = 0.53 (ethyl acetate/petrol ether 1:1). **IR (ATR):** 2942 (br), 2835 (w), 2161 (w), 2032 (w), 1254 (m) cm^{-1}. **UV/Vis** ($CHCl_3$) λ_{max} (ε): 295 (sh, 35600), 316 (62000), 336 (98500), 365 (sh, 10000), 391 (3090), 434 (1870), 489 (1270), 545 (714). **^{1}H NMR** (400 MHz, $CDCl_3$) δ 3.35 (s, 24H; OCH_3), 3.32 (s, 24H; OCH_3). **^{13}C NMR** (100 MHz, $CDCl_3$) δ 139.1, 137.6, 107.9, 107.7, 96.2, 88.7, 79.5, 52.1, 51.9. **ESI HRMS *m/z*:** calcd for $C_{44}H_{48}O_{16}Na^+$ ($[M + Na]^+$) 855.2834, found 855.2835.

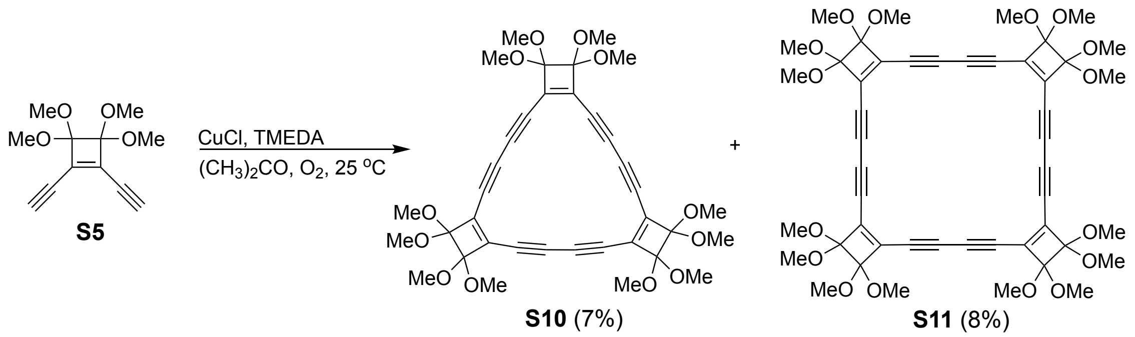

Compound S10: The titled compound **S10** was synthesized as described in the literature[18]. Freshly prepared CuCl (5.0 g, 51 mmol) was stirred in acetone (50 mL) under N_2. TMEDA (2.7 mL, 18 mmol) was added to this suspension and the resulting opaque, pale blue solution was stirred for 30 minutes. The solid was allowed to settle and the supernatant solution (15.0 mL, 5.29 mmol) was withdrawn and immediately added to a vigorously stirred solution of compound **2** (1.15 g, 5.17 mmol) in acetone (300 mL) at 25 °C with a $CaCl_2$ drying tube and under an O_2 atmosphere for 2 h. Upon completion, the reaction was diluted with $CHCl_3$ (300 mL) and the organic phase was washed with water (2 x 600 mL). The organic extracts were combined and dried over $MgSO_4$, and the solvent was removed in *vacuo* at 30 °C. The crude was purified by column chromatography, SiO_2 (petroleum ether/ethyl acetate 8:2) to yield compound **S10** (76 mg, 7%) as a yellow solid and compound **S11** (96 mg, 8%) as a red solid.

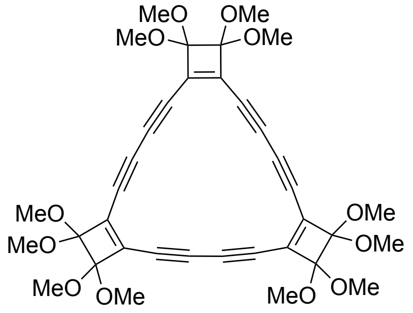

***R**$_f$* = 0.77 (ethyl acetate/petrol ether 1:1). **IR (ATR):** 2940 (m), 2836 (w), 1742 (w), 1252 (s), 1083 (s) cm^{-1}. **UV/Vis** ($CHCl_3$) λ_{max} (ε): 328 (42200), 345 (78100), 393 (12900), 403 (16100). **^{1}H NMR** (400 MHz, $CDCl_3$): δ 3.71 (s, 36H, OCH_3). **^{13}C NMR** (100 MHz, $CDCl_3$): δ 139.5, 109.8, 90.1, 81.9, 52.5. **MALDI-TOF *m/z*:** calcd for $C_{35}H_{33}O_{11}^+$ ($[M – OMe]^+$), 629.202 found 629.308.

Compound S11: The titled compound **S11** (96 mg, 8%) was obtained as a red solid as described in the above procedure.

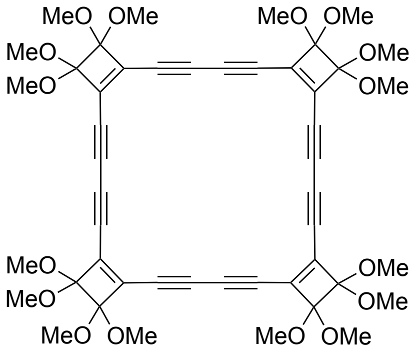

***R**$_f$* = 0.60 (ethyl acetate/petrol ether 1:1). **IR (ATR):** 2942 (m), 2837 (w), 1252 (s), 1084 (s) cm^{-1}. **UV/vis** ($CHCl_3$): λ_{max} (ε): 299 (27300), 313 (sh, 36900), 317 (37600), 335 (85000), 342 (sh, 69400), 361 (184600), 408 (sh, 5850), 436 (sh, 4080), 480 (2860), 525 nm (1510). **^{1}H NMR** (400 MHz, $CDCl_3$): 3.37 (s, 48H, OCH_3) ppm. **^{13}C NMR** (100 MHz, $CDCl_3$): 138.3, 108.2, 88.5, 79.6, 52.1 ppm. **MALDI-TOF *m/z*:** calcd for $C_{47}H_{45}O_{15}^+$ ([M – OMe]$^+$), 849.28 found 849.50.

Compound 4: Trifluoroacetic acid (TFA, 1.00 mL) and H_2O (26 μL, 1.5 mmol) were added to **3** (8.0 mg, 0.012 mmol) in a vial. The solution was stirred for 3.5 h at 25 °C, with the vial wrapped in aluminum foil to avoid exposure to light. Solvent removal under a flow of N_2 gas and then under high vacuum afforded compound **4** (5.7 mg, 94%) as a wine-red to grey solid. The solid form of **4** was light sensitive and slowly decomposed, therefore the compound was stored at –20 °C as a dilute $CHCl_3$ solution and shielded from light.

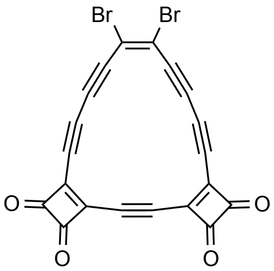

IR (ATR): 2172 (w), 2123 (w), 1793 (s), 1781 (s) cm^{-1}. **UV/Vis** ($CHCl_3$) λ_{max} (ε): 330 (46100), 350 (58300). **^{13}C NMR** (126 MHz, $CDCl_3$) δ 190.4 (C=O), 189.7 (C=O), 186.9 (C=C), 182.4 (C=C), 129.9 (C=CBr), 118.7 (C≡C), 107.9 (C≡C), 98.0 (C≡C), 82.9 (C≡C), 74.8 (C≡C).

Compound S12: Trifluoroacetic acid (TFA, 1.00 mL) and H_2O (26 μL, 1.45 mmol) were added to compound **S11** (8.0 mg, 0.013 mmol) in a vial. The solution was stirred for 3.5 h at 25 °C with the vial wrapped in aluminum foil to avoid exposure to light. Solvent removal under a flow of N_2 afforded a deep red solid, which was quickly dissolved in $CDCl_3$ (ca. 0.5 mL) for NMR measurement. The solid form of compound **S12** was light sensitive and decomposed to a black solid within an hour, therefore the compound was stored at –20 °C as a dilute $CHCl_3$ solution shielded from light.

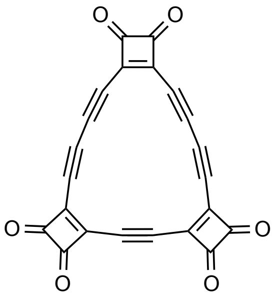

IR (ATR): 1783 (s) cm^{-1}. **UV/Vis** ($CHCl_3$) λ_{max} (ε): 290 (13900), 329 (36700), 336 (sh, 35100), 350 (31800), 377 (sh, 8120), 403 (sh, 3540), 440 (sh, 2120), 523 (sh, 1440), 583 (sh, 902). **^{13}C NMR** (126 MHz, $CDCl_3$) δ 191.3, 189.4, 188.44, 188.39, 184.4, 184.2, 110.0, 108.6, 108.5, 85.1, 84.6.

Compound S13: Trifluoroacetic acid (TFA, 1.00 mL) and H_2O (25.0 μL, 1.38 mmol) were added to compound **S9** (10 mg, 0.012 mmol) in a vial. The solution was stirred for 4 h at 25 °C with wrapped in aluminum foil to avoid light. Solvent removal by flushing N_2 gas afforded a deep red solid, which was quickly added $CDCl_3$ (ca. 0.5 mL) for NMR measurement. The solid form of compound **S13** was light sensitive and decomposed to a black solid within hours, the compound was stored in a diluted $CHCl_3$ solution shielding from light at –20 °C.

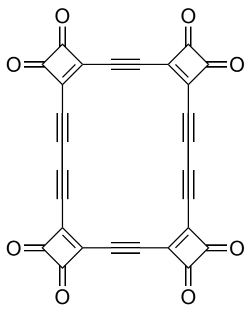

IR (ATR): 1784 (s) cm^{-1}. **UV/Vis** ($CHCl_3$) λ_{max} (ε): 276 (5734), 285 (5258), 343 (10602), 350 (9980), 380 (9040), 477 nm (707). **^{13}C NMR** (126 MHz, $CDCl_3$) δ 189.6, 189.3, 181.5, 178.6, 108.5, 107.8, 83.0.

Compound S14: The synthesis was adapted from a reported synthetic procedure[20]. All reaction vessels were kept under nitrogen and wrapped with aluminium foil to exclude light. Concentrated H_2SO_4 (0.2 mL) was dropped onto compound **S11** (10 mg, 0.011 mmol) to form a dark red emulsion which was stirred for 5 minutes at 21 °C. Then, $(CH_2Cl)_2$ (10 mL) was added, and the resulting solution was stirred vigorously for 10 minutes. The clear orange organic phase containing traces of acid was transferred *via* a syringe to a flask containing $CaCO_3$ (0.4 g). The extraction with $(CH_2Cl)_2$ was repeated until the organic extracts were colourless. The combined organic phases were filtered with 0.45 µm PTFE syringe filters to yield a clear orange solution. The solvent was removed *in vacuo* and the crude material was dried under a high vacuum in the dark to yield compound **S14** (1.2 mg, 2.0 µmol, 21%) as an unstable, light-sensitive red solid which was stored in the dark at –20 °C and observed to decompose over a couple of days under these conditions.
IR (ATR): 1785 (s) cm^{-1}. **UV/Vis** ($CHCl_3$) λ_{max} (ε): 269 nm (6205), 296 (4301), 352 (6524), 363 (7178), 380 (6205). **^{13}C NMR** (101 MHz, $CDCl_3$): 190.4, 180.0, 107.4, 82.6 ppm.

Unsuccessful routes to cyclo[*N*]carbons (*N* = 16, 20, 24):

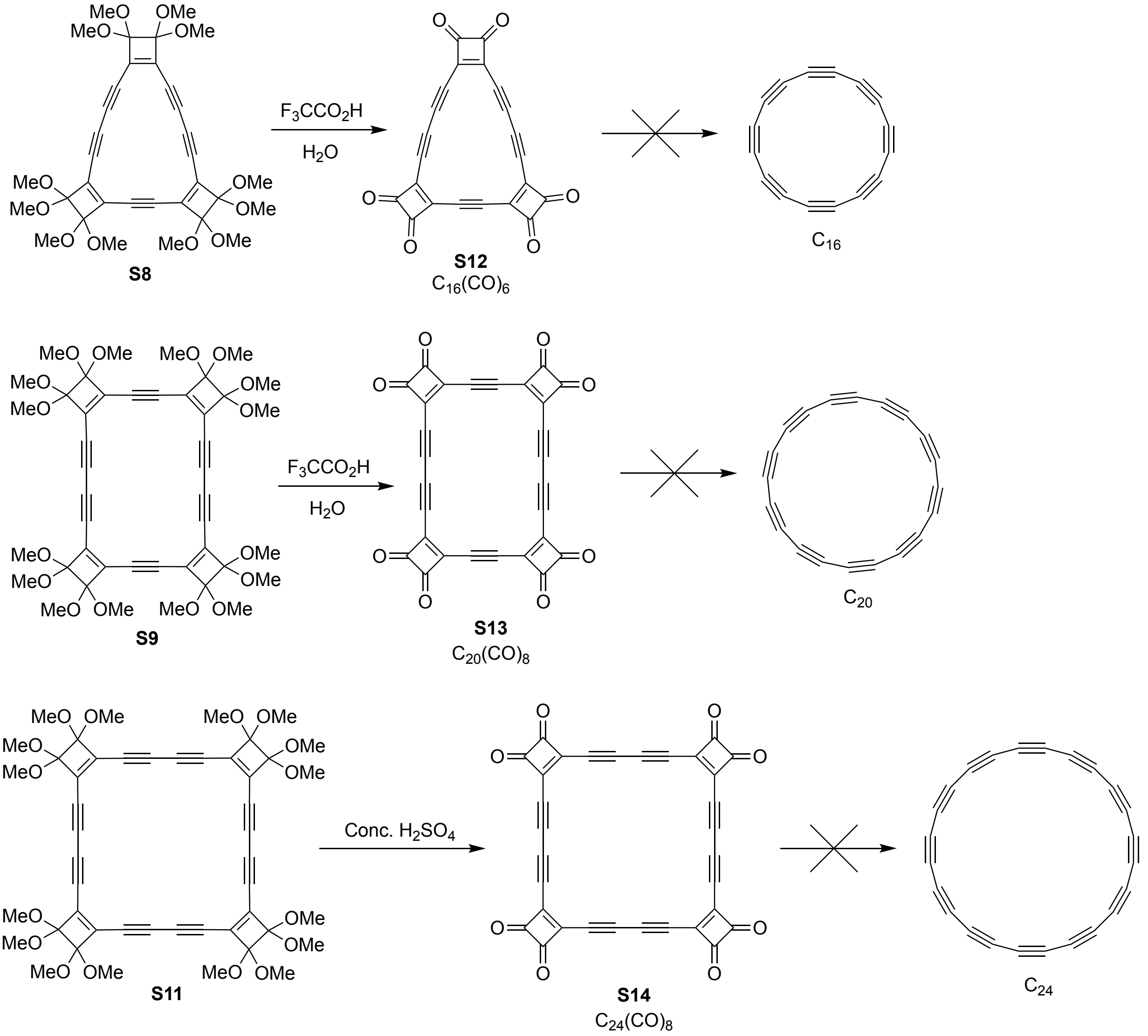

Scheme S2. Unsuccessful routes to cyclo[*N*]carbons (*N* = 16, 20, 24).

We explored two routes to C_{16}, as summarised in Fig. 2 and Scheme S2. Deprotection of **S8** to give **S12** proved difficult because of the high reactivity of these compounds (e.g., using concentrated sulfuric acid, as in case of C_{18}, was not viable). After testing many reaction conditions, we found that **S8** can be converted to **S12** using trifluoracetic acid containing water (2.5% by volume), but

unfortunately the anti-aromatic cyclocarbon oxide **S12** is too unstable for sublimation, preventing further work on this route to C_{16}. We encountered the same problem when attempting to prepare other cyclo[4*n*]carbons, C_{20} and C_{24}, from their anti-aromatic carbon oxide precursors **S13**, $C_{20}(CO)_8$ and **S14**, $C_{24}(CO)_8$ (see Scheme S2).

A5. Comparison of ^{1}H NMR spectra:

Macrocycle **S8** has a circuit of 16 sp^1 or sp^2 hybridised carbon atoms and it is anti-aromatic, in contrast to the corresponding C_{18}-precursor **S10**, which features a ring of 18 sp^1 or sp^2 hybridised carbon atoms and is aromatic. This difference in electronic structure is reflected by the ^{1}H NMR spectra (see Fig. S2). The chemical shifts of the methoxy resonance probe the aromatic ring current in compound **S10** and the antiaromatic ring currents in **S8**, **S9**, **S11**, and **3**, indicating that **S10** is aromatic whereas **S8**, **S9**, **S11**, and **3**are antiaromatic, as predicted by Hückel's rule. Thus the ^{1}H NMR spectrum (Fig. S2) of **S8** reveals that the methyl protons (δ_H 3.23 and 3.22 ppm, in $CDCl_3$) are more shielded than those of **S5** (non-aromatic; δ_H 3.50 ppm) whereas those of **S10** are deshielded (δ_H 3.71 ppm). The presence of paratropic and diatropic ring currents in **S8** and **S10**, respectively, is supported by density functional theory (DFT) calculations (see Fig. S25–S27).

A6. Stability tests:

The thermal decomposition of compounds **S12** and **4** at 25 °C was monitored by UV-vis spectroscopy as measured in $CHCl_3$ (Fig. S5). To perform these experiments, concentrated solutions of **S12** and **4** in $CHCl_3$ were prepared, such that diluting 100 µL of the concentrated solution into a 2.5 mL of $CHCl_3$ in a 10-mm pathlength cuvette gave a UV-vis absorption spectrum with an optical density of ca. 0.5 at λ_{max}. For solution-state measurements, the sample was kept in the dark and recorded by UV-vis spectrometer at a certain time interval, giving results shown in Fig. S5a) and b). After learning the decomposition of compounds **S12** and **4** was minimum as a solution state in a period of 60 min, the solid-state samples were then prepared, such that diluting 100 µL of the concentrated solution into a 2.5 mL of $CHCl_3$ in a 10-mm pathlength cuvette gave a UV-vis absorption spectrum with an optical density of ca. 0.5 at λ_{max} as the initial record at 0 min. Four more samples with the same concentration were prepared. These samples were wrapped with aluminum foil to avoid light exposure. For solid-state measurements, the solvent was quickly evaporated under a flow of N_2 giving a solid sample. After keeping the solid-state sample in the dark for a certain time, $CHCl_3$ (2.6 mL) was added and recorded by UV-vis spectrometer.

Although compound **4** is antiaromatic, like **S12**, it is substantially more stable. At room temperature in the dark, solid samples of precursor **S12** decompose to an extent of about 50% in 5 minutes, whereas under the same conditions, the extent of decomposition of **4** is only about 10% (Fig. S4 and S5). The greater thermal stability of **4** made it possible to deposit this compound on a surface by sublimation under ultra-high vacuum.

A7. Additional STM and AFM data:

Additional STM and AFM data on the precursor **4**, and intermediates **5**, **6** are shown in Figs. S6, S7, S8, respectively.

Additional STM and AFM data on C_{16} are shown in Figs. S9 and S10.

The outcome of an unsuccessful attempt of the on-surface synthesis of C_{16} is shown in Fig. S11. In this case, the carbon ring opened. In 14 attempts, applying voltage pulses at the location of an individual precursor molecule, we generated four C_{16} molecules. We found four additional C_{16} molecules that have formed in the vicinity of applied voltage pulses. Unsuccessful events resulted mostly in molecules being picked up by the tip.

Additional STM and AFM data for the adsorption geometry and adsorption site determination of C_{16} is shown in Figs. S13–S16.

Additional STM and AFM data for the charge-state determination and switching of C_{16} are shown in Figs. S12,S17, S18 and in Tables S2 and S3.

A8. Computational results (gas phase):

Complete active space self-consistent field (CASSCF) calculations on C_{16}^0 with an active space spanning 12 electrons and 12 orbitals reveal a single-reference character of 71%, contrasting the single determinant-dominated (~95%) wavefunction reported previously for 4*n*+2 cyclocarbons[21,22]. Adding dynamic correlation, either via the n-electron valence state perturbation[23] (NEVPT2) or complete active space second-order perturbation (CASPT2) theory results in a significant (0.7–1.5 eV) increase of both the ionization potential (IP) and the electron affinity (EA), as well as small changes in the optimized geometry (0.05 Å increase in the ring radius; Table S4), which was obtained using a grid search at the NEVPT2 level. CASPT, NEVPT2, and equation-of-motion coupled clusters (EOM-CCSD) all give very similar EAs (~2.1 eV) and vertical excitation energies (2.1–2.2 eV), but differ more in the predicted IPs (IP_{NEVPT2} = 8.76 eV; IP_{CASPT2} = 8.11 eV; IP_{CCSD} = 8.27 eV).

Most previous calculations on cyclocarbons were done using density functional theory, with the range-separated ωB97XD[24] hybrid functional being a popular choice[21,22,25]. In the case of C_{16}, ωB97XD reproduces results obtained by correlated methods very well (Table S4 and Fig. S19), confirming it to be a suitable choice for C_{16}. We also note that the double hybrid DSD-PBEP86[26] functional is a very good choice, giving excellent correspondence with the optimized geometry at the NEVPT2 level.

Figs. S19–S22 show molecular orbitals of C_{16}^0, C_{16}^+, C_{16}^-, and C_{16}^{2-}, while Figs. S25–S28 show $NICS(0)_{zz}$ plots for **S8**, **S10**, **3**, the C_{16} |2020> configuration, C_{18} ground state, and the C_{16} |2200> configuration, respectively, all obtained at the ωB97XD/def2-TZVP level of theory. The ring current of C_{16}^0 was calculated to be –25 nA/T at this level using the SYSMOIC code[27].

Ring geometry and deformation. At the ωB97XD/def2-TZVP level of theory, C_{16}^0 and C_{16}^+ optimize to D_{8h} minima with bond-length alternation (BLA) but no bond angle alternation (BAA; see Table S5). The D_{8h} geometry of C_{16}^- has an imaginary vibrational frequency distorting it to C_{8h} (Fig. S29), which shows both BLA and BAA. The dianion C_{16}^{2-} optimizes to D_{8h}, but shows only BAA and no BLA.

Relaxed ring distortion scans along the longest diagonal (connecting C1 and C9) and two smaller ones (C1-C8 and C1-C7) show that distorting the neutral ring is ~10% more difficult than distorting the cation, according to a harmonic fit (Fig. S30). The distortion of the anion is about ~20% easier compared to the neutral ring.

A9. Computational results (surface adsorption):

Using periodic boundary conditions and the PBE-D3BJ density functional, we found three adsorption sites for both C_{16}^0 and C_{16}^-, centered on either Na^+, Cl^-, or a lattice bridge site (Table S1). The adsorption energy of C_{16}^0 is 36–40 meV/atom, very similar to the value obtained using the same level of theory for C_{18}^0 (ref. 28). C_{16}^0 slightly prefers the Cl^--centered site, undergoing a small amount of distortion (0.16 Å) from the gas-phase optimized shape. On the other hand, C_{16}^- prefers the bridge site (~90 meV/atom), undergoing a larger amount of distortion (1.25 Å). Slight bond-angle alternation (11–15°) is evident in the three calculated structures of C_{16}^0, and it becomes more pronounced in the anion (18–26°). These values may be overestimates[24], as the pure PBE functional predicts bond-angle alternation (15°) even in gas-phase C_{16}^0, while a more accurate hybrid functional (ωB97XD) predicts none in the gas phase.

To analyze the redistribution of electron density upon deformation and adsorption, we calculated the Natural Bond Orbital (NBO) charges[29] of (a) gas-phase optimized geometries of C_{16}^0 and C_{16}^-, as well as optimized geometries of C_{16}^0 and C_{16}^- adsorbed at the bridge site without the surface (b), or with a cluster model of the surface containing two layers (100 atoms) (c), at the ωB97XD/def2-TZVP level of theory. In line with previous findings[30,31], our results (Fig. S31) show that the introduction of BAA induces charge separation, which is much more pronounced in case of the anion than the neutral molecule. While the presence of the surface causes the appearance of BAA in both cases, it is worth noting that the effect of the surface on the NBO charges is opposite in C_{16}^0 and C_{16}^- (Figs. S30 b and c). The electrostatic interactions between C_{16}^0 and the surface ions are not strong enough to significantly distort the lattice, so the surface slightly attenuates the charges. In C_{16}^-, the Coulomb interactions between individual carbons and surface ions are much stronger, leading to larger lattice distortion and the increase of charge separation in the cyclocarbon.

C_{16}^0 and C_{16}^- are found to interact strongly with different geometries of NaCl island step-edges. In bays (B1, B2, and B3; Table S1), the adsorption energies of both molecules (~65 and ~150 meV/atom for C_{16}^0 and C_{16}^-, respectively) are nearly twice as large as those for the pristine NaCl surface. Interactions with the edge (E) of both C_{16}^0 (67 meV/atom) and C_{16}^- (128 meV/atom) are slightly larger than with the kink (K) and the side of the wall (W), i.e., nonpolar step-edge. In all cases, we find that bond-length alternation is preserved, although it does get slightly reduced in case of some step-edge geometries. In most cases, appreciable BAA is induced by interactions with the NaCl step edges (Table S1).

B. Supplementary Figures

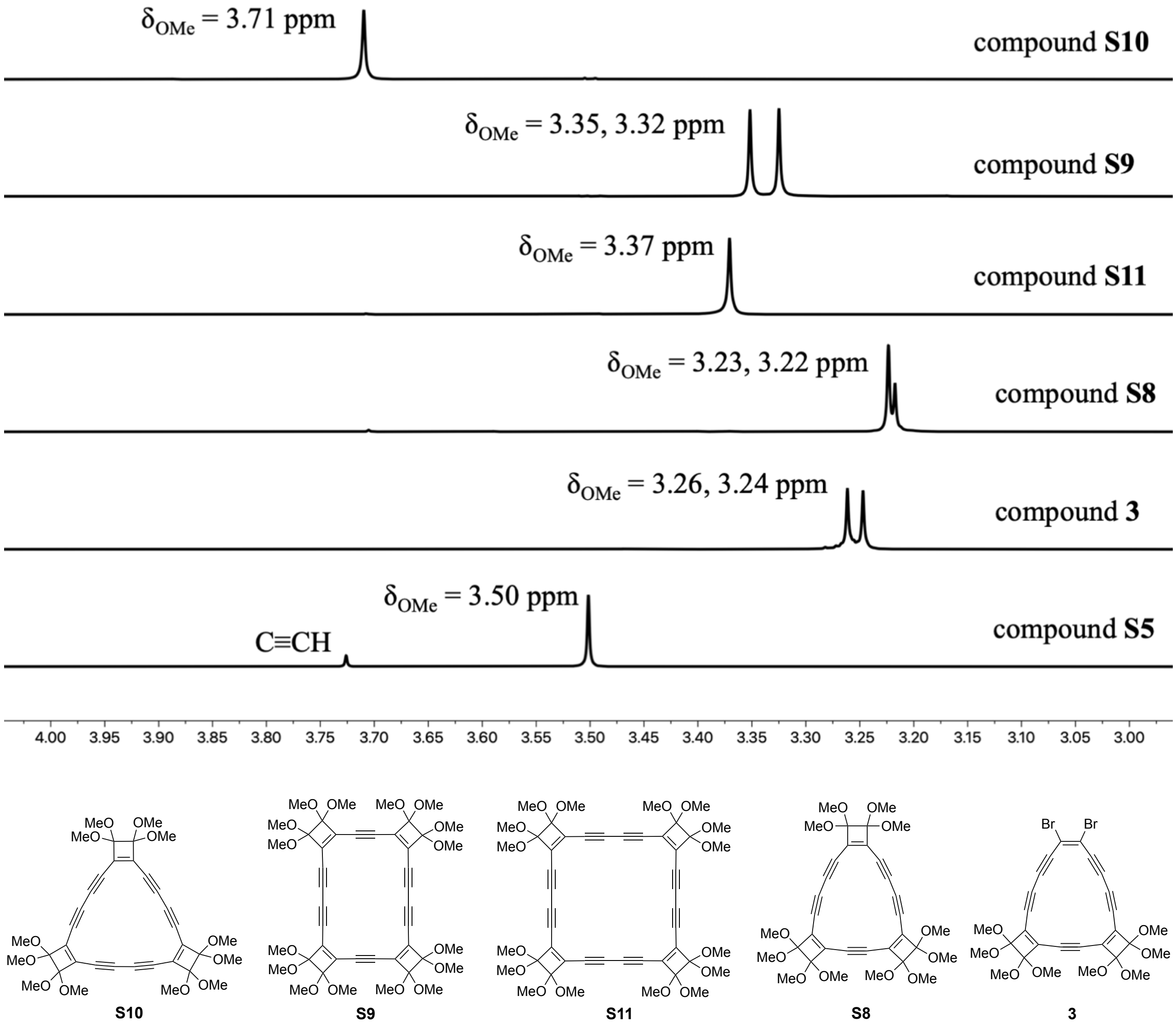

Fig. S2. Partial ^{1}H NMR spectra of compounds **S10**, **S9**, **S11**, **S8**, **3**, and **S5** (400 MHz, $CDCl_3$) showing evidence for an aromatic ring current in compound **S10** and anti-aromatic ring currents in compounds **S9**, **S11**, **S8** and **3**.

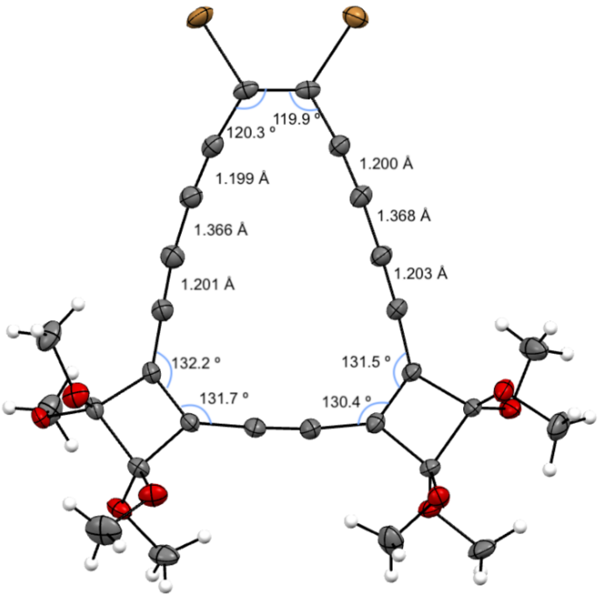

Fig. S3. X-ray crystallographic structure of compound **3** (thermal ellipsoids are drawn at 30% probability). The crystallographic data have been deposited with the Cambridge Crystallographic Data Center (CCDC 2240722).

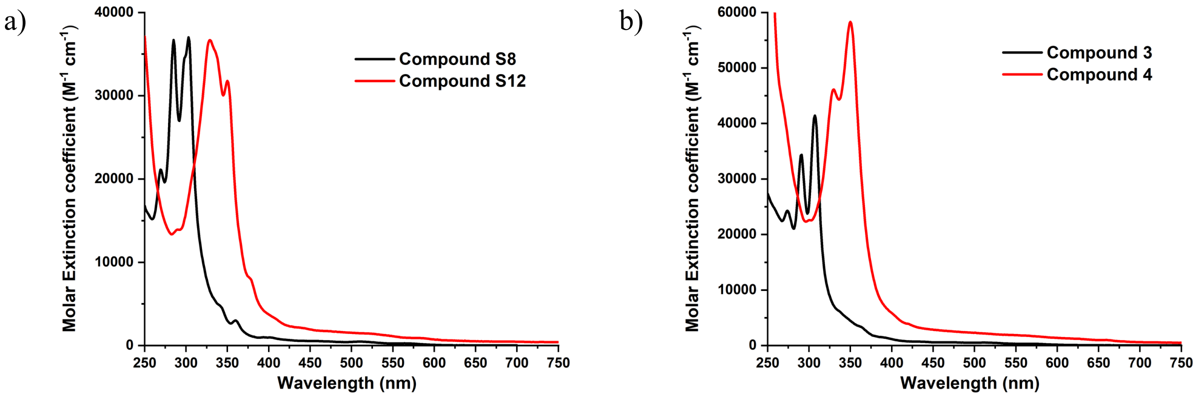

Fig. S4. Quantitative UV-vis spectra of a) compounds **S8** and **S12** and b) **3** and **4** as measured in $CHCl_3$. (Note that only minimum values were estimated for compound **S12** and **4** due to decomposition.)

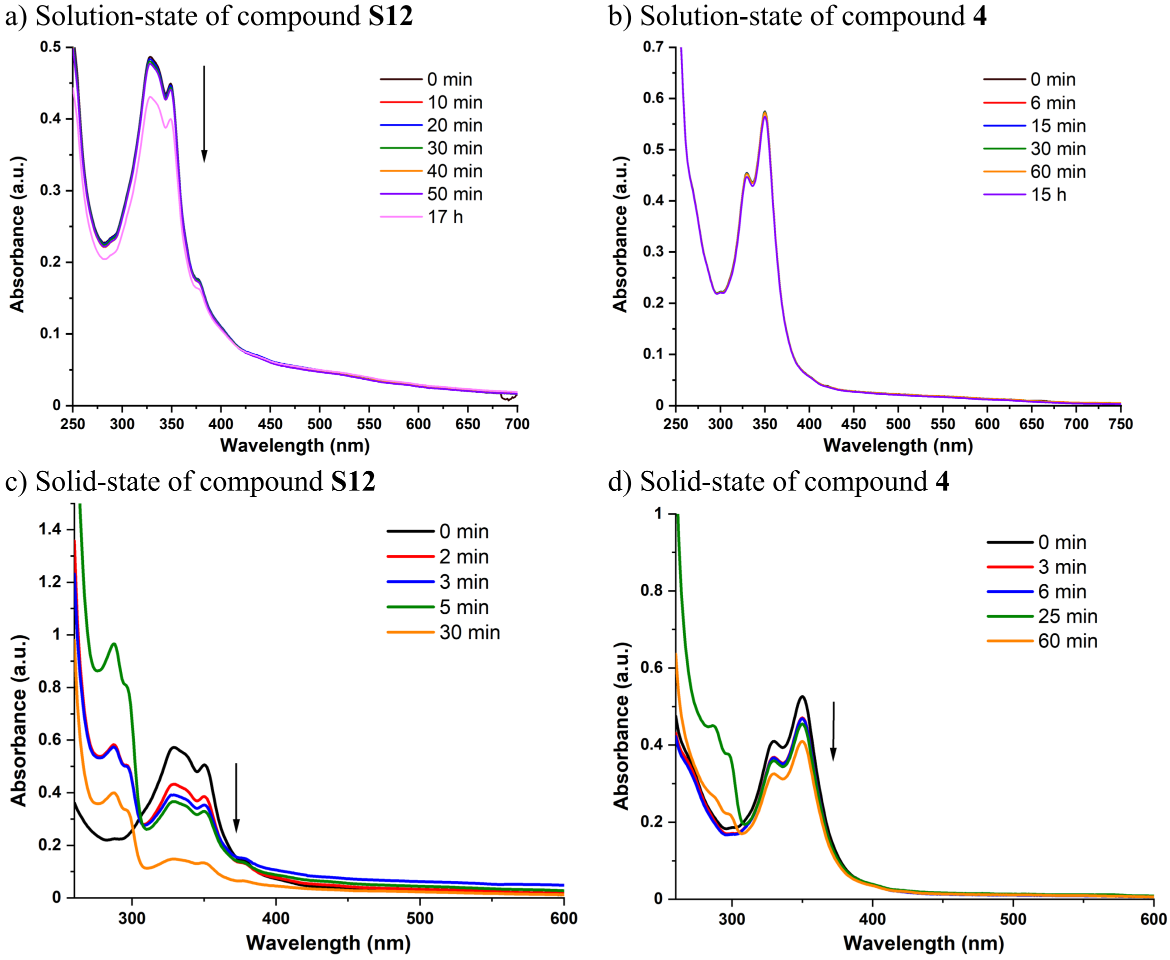

Fig. S5. Stability tests for a) compound **S12** and b) compound **4** in a solution of $CHCl_3$ at 25 °C, c) compound **12** and d) compound **4** in the solid state in the dark at 25 °C; all monitored by UV-vis absorption spectroscopy in $CHCl_3$.

Additional AFM and STM data:

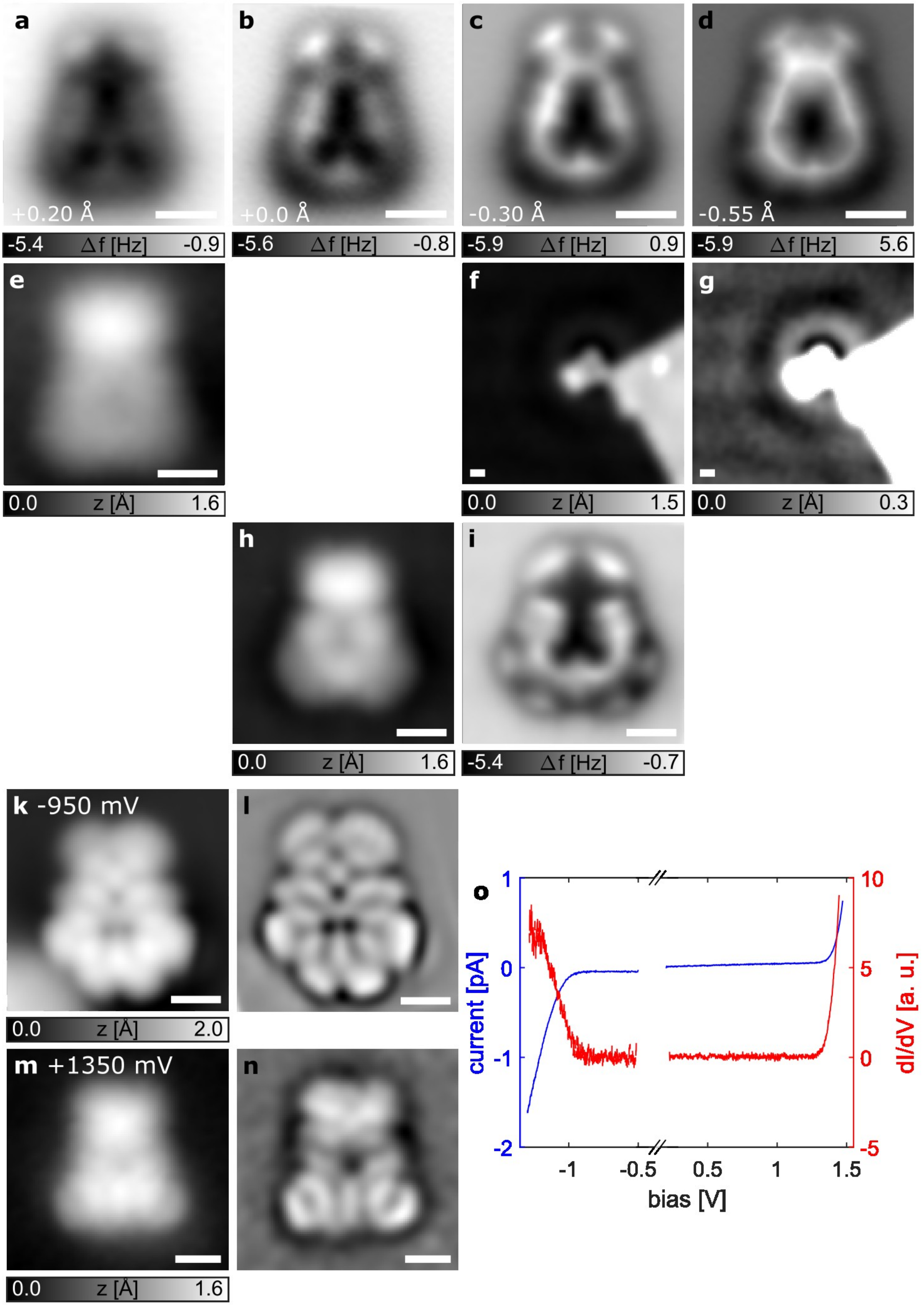

Fig. S6. Characterization of precursor **4**. (a–d) AFM data recorded at the tip-height offsets indicated in the panels. (e) STM image of **4** (I = 0.2 pA, V = 0.2 V). (f) Larger scale topographic image of **4** next to a 3rd layer NaCl island (I = 0.2 pA, V = 0.2 V), with the entire range of tip height shown. (g)

same data as (e) with contrast limitation to highlight the electronic interface state and its scattering pattern around the molecule, indicating **4** to be charged. (h) STM of **4** adsorbed on trilayer NaCl (I = 0.25 pA, V = 0.2 V). (i) AFM signal acquired simultaneously with (h), i. e., Δf recorded along the constant-current STM topography. In this mode, detailed contrast at the carbonyl groups is visible. (k) STM at the onset of the first ionic resonance at negative bias (I = 1.0 pA, V = –0.95 V). (l) The same data as shown in (k) after applying a Laplace filter. (m) STM at the onset of the first ionic resonance at positive bias (I = 0.2 pA, V = 1.35 V). (n) The same data shown in (m) after applying a Laplace filter. (o) $I(V)$ spectrum (blue) and numerically derived differential conductance spectrum (red) of **4** adsorbed on bilayer NaCl. All scale bars correspond to 5 Å.

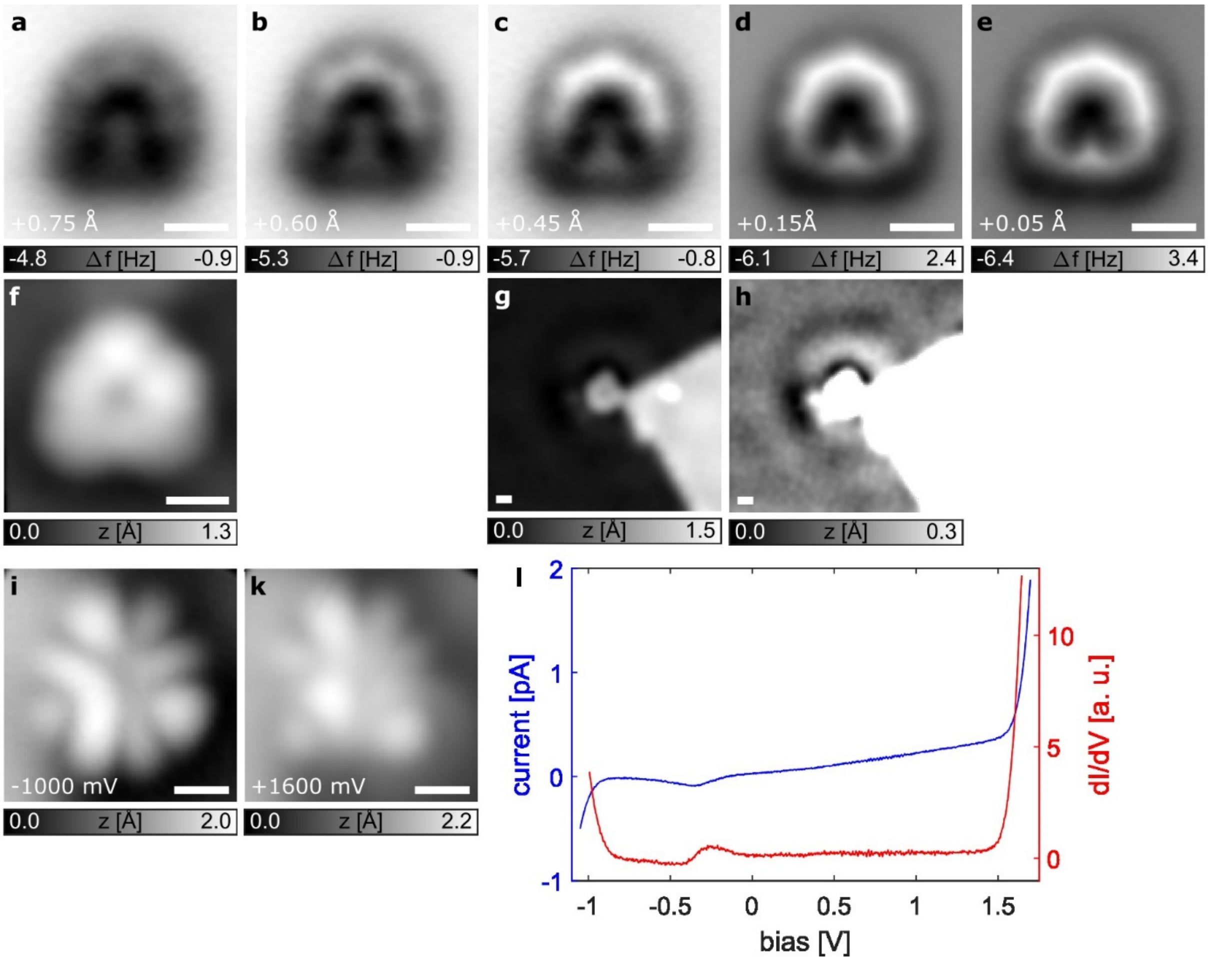

Fig. S7. Characterization of intermediate **5**. (a–e) AFM images recorded at the tip-height offset indicated in the panels with respect to a STM setpoint of $I = 0.5$ pA and $V = 0.2$ V. (f) STM image of **5** ($I = 0.5$ pA, $V = 0.2$ V). (g) Larger scale STM image of **5** next to a 3rd layer NaCl with the entire topographic contrast shown ($I = 0.2$ pA and $V = 0.2$ V). (h) The same data as (g) with contrast limitation to highlight the electronic interface state and its scattering pattern around the molecule, indicating **5** to be charged. (i and k) STM images recorded with a metallic tip, at the onsets of the first ionic resonance at negative and positive bias voltage, respectively ($I = 0.2$ pA). (l) $I(V)$ spectrum (blue) and numerically derived differential conductance spectrum (red) of **5** adsorbed on bilayer NaCl. The feature at –0.3 V relates to the onset of the Cu(111)/NaCl interface state[32]. All scale bars correspond to 5 Å.

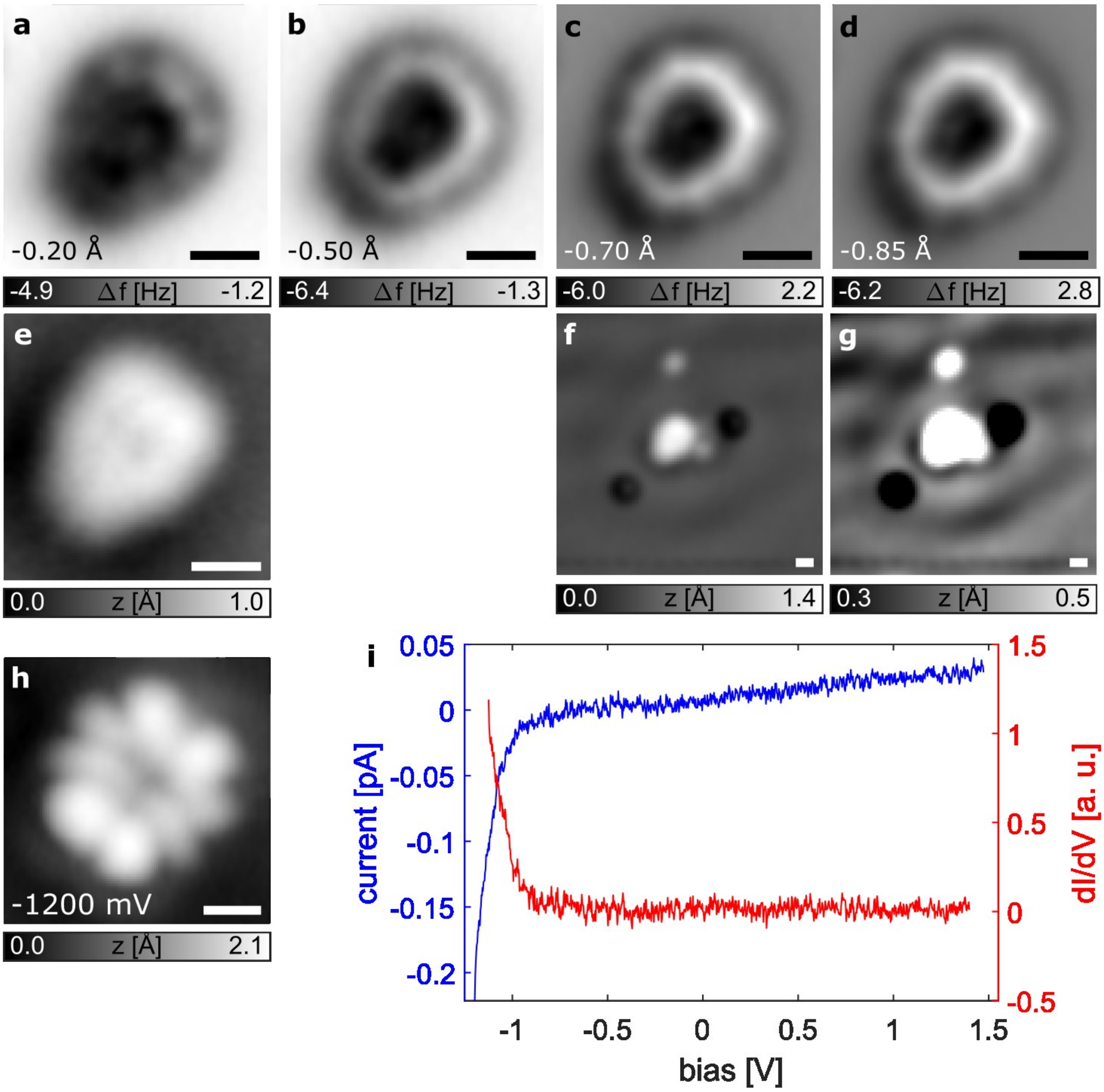

Fig. S8. Characterization of intermediate **6**. (a–d) AFM images at tip-height offset as indicated in the panels. (e) STM of **6** (I = 0.2 pA, V = 0.2 V). (f) Large scale STM image of **6** (I = 0.2 pA, V = 0.1 V) with the entire topographic contrast shown. (g) The same data as shown in (f) but with contrast limitation to highlight the electronic interface state and its scattering pattern around the molecule, indicating **6** to be charged. (h) STM image recorded at the onset of the first ionic resonance at negative bias voltage (I = 0.2 pA, V = –1.2 V). (i) $I(V)$ spectrum (blue) and numerically derived differential conductance spectrum (red) of **6** adsorbed on bilayer NaCl. All scale bars correspond to 5 Å.

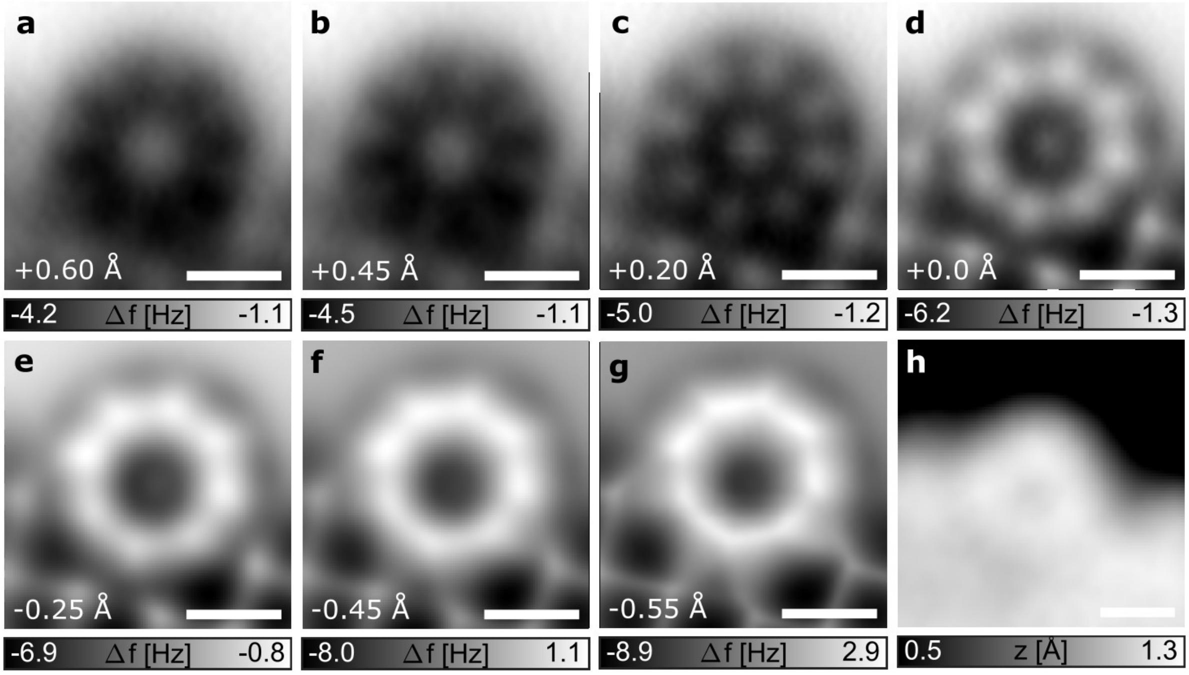

Fig. S9. Additional experimental images of C_{16}^{0} in a bay of 3rd layer NaCl, shown in Fig. 3j-m. (a–g) AFM data at tip-height offsets as indicated in the panels. (h) STM image of the same molecule (I = 0.2 A, V = 0.2 V). All scale bars correspond to 5 Å.

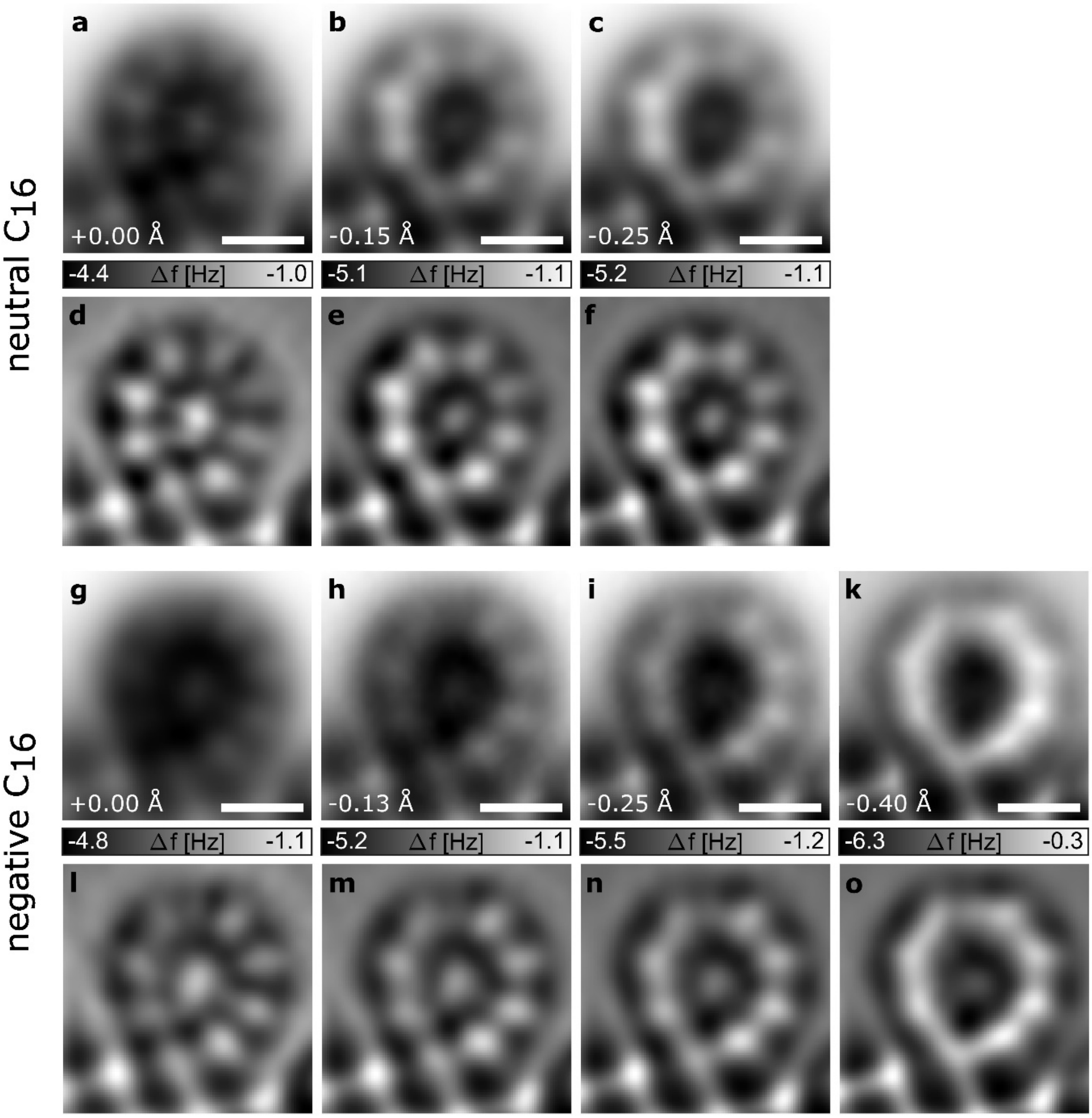

Fig. S10. Additional AFM data of C_{16} shown in Fig. 4; (a–c) AFM images of C_{16}^{0} at tip-height offsets as indicated in the panels. (d–f) The same data as shown in (a–c) after applying a Laplace filter. (g–k) AFM images of C_{16}^{-} at the tip-height offsets indicated in the panels. (l–o) The same data as shown in (g–k) after applying a Laplace filter. All scare bars correspond to 5 Å.

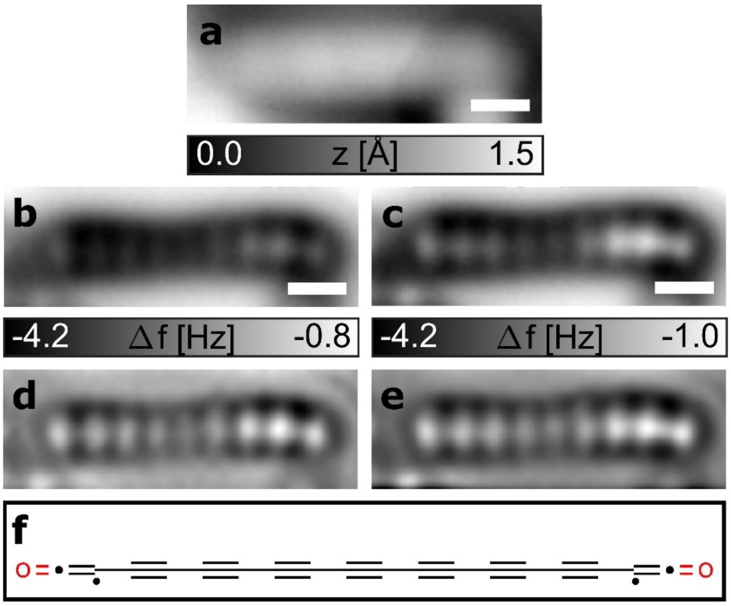

Fig. S11. Linear product after unsuccessful attempted on-surface synthesis of C_{16}. The product was formed by applying voltage pulses of $V = 3.4$ V, $I = 1$ pA above the precursor **4**. (a) STM image ($I = 0.2$ pA, $V = 0.2$ V). (b and c) AFM images of the same molecule recorded at tip-height offsets of +0.2 Å for panel a and 0 Å for c. (d and e) Laplace filtered data of (b) and (c). (f) Tentatively proposed structure of this reaction product. All scale bars correspond to 5 Å.

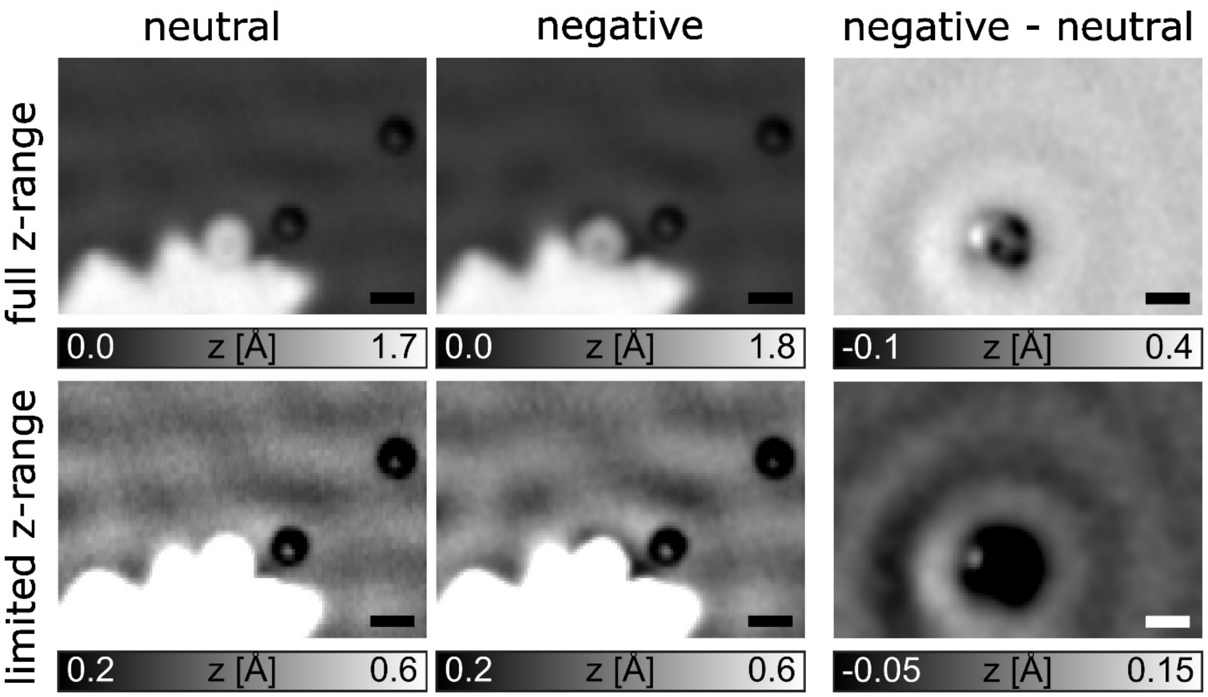

Fig. S12. STM images resolving the effect of charge-state switching of C_{16} by interface-state scattering (same molecule as in Fig. 4 and Fig. S10). Top row: STM images of C_{16}^0 (left) and C_{16}^- (center) molecule (I = 0.2 pA, V = 0.05 V). The charge state was changed by ramping the bias, see Fig. S18b. The right panel shows the difference of the topographic images. Bottom row: Same data as top row with limited contrast to highlight the interface state scattering pattern. The concentric ring-shaped pattern in the difference images (right panels) proves a change of charge state of C_{16} between the left and middle images. All scare bars correspond to 10 Å.

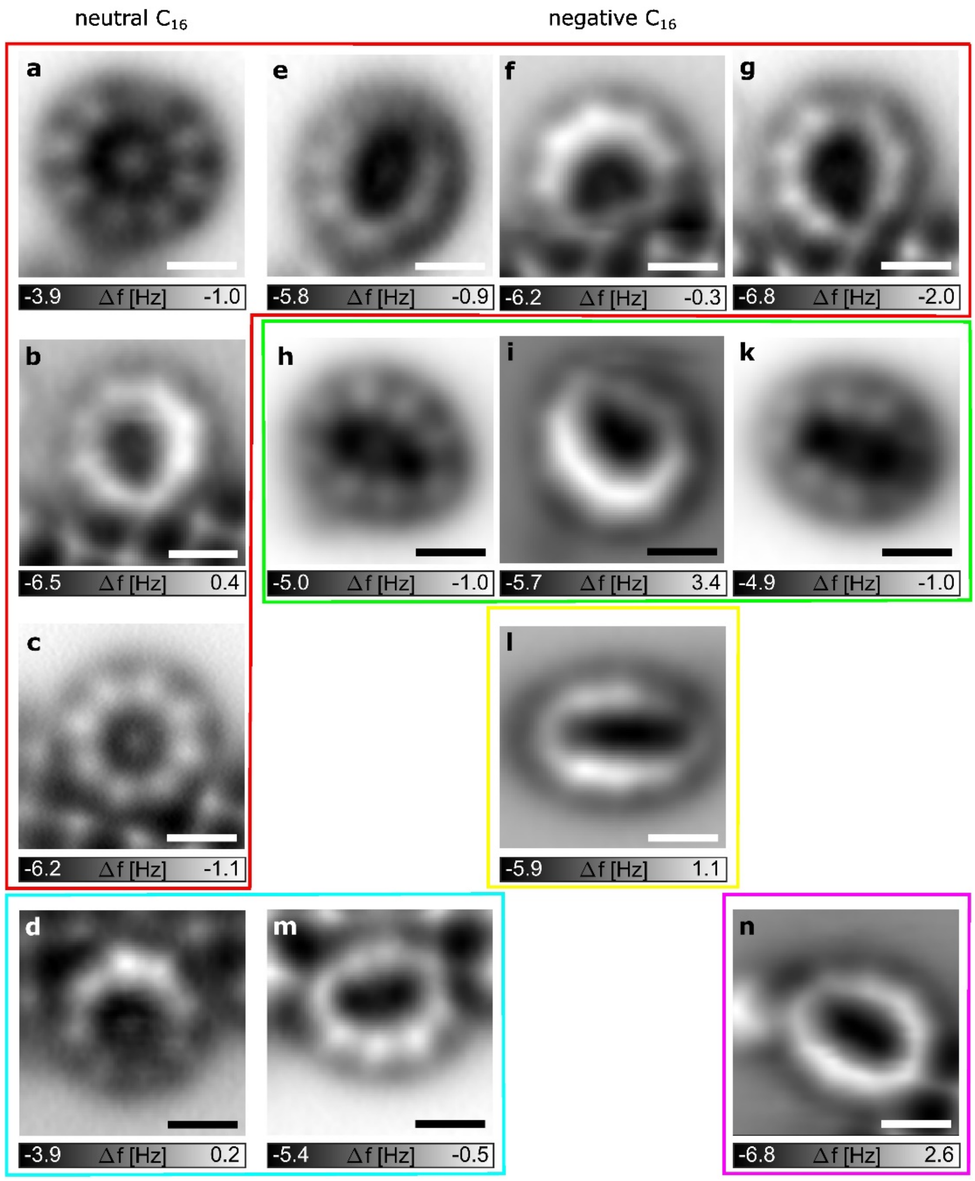

Fig. S13. AFM images of C_{16}^0 (a–d) and C_{16}^- (e–n). Color frames indicate that images show the same individual molecule at different adsorption sites (and in different charge states). Note that overall, C_{16}^- shows more significant deviations from a circular shape than C_{16}^0. Typically, that is, at most adsorption sites, we observed charge state bistability of neutral and anionic charge states of C_{16}. In some cases, the molecule changed its adsorption site when the voltage was ramped up to switch the charge state. The molecule in Fig. 4 did not move when the charge state was switched repeatedly and for many times, nor when the orbital density was mapped, presumably due to a very stable adsorption site at a 3rd layer NaCl island (bay 1, see Fig. S55 and Table S1). Scale bars 5 Å.

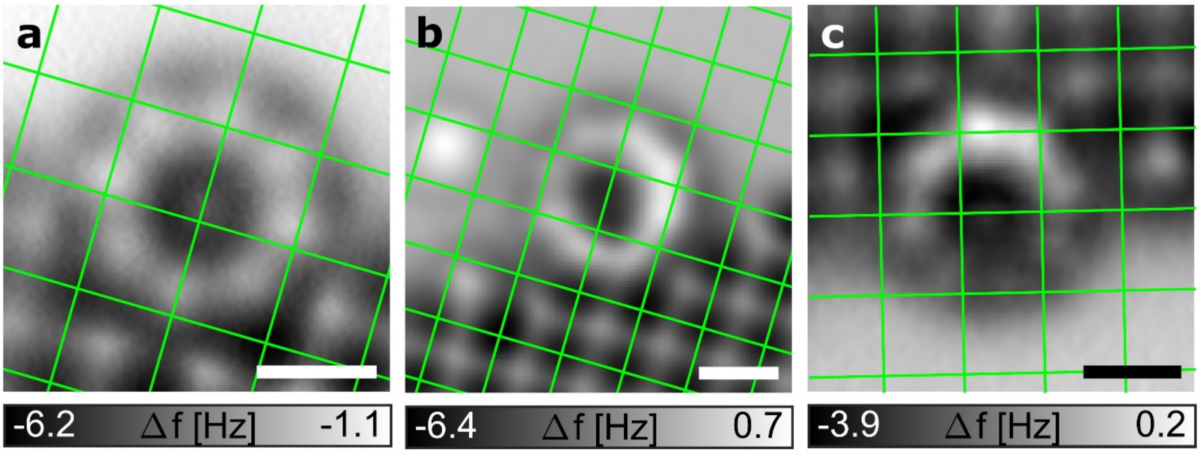

Fig. S14. Experimental adsorption-site determination for C_{16}^0 on bilayer NaCl on Cu(111). We never observed C_{16}^0 stably isolated on the NaCl surface, but always near a 3rd layer NaCl step edge. In all panels the NaCl lattice is indicated, crossings corresponding to sites of Na^+ ions in the 3rd layer and Cl^- ions in the second layer NaCl, i.e., below the molecule. As can be seen, we observe C_{16}^0 adsorbed at various adsorption sites, indicative of a shallow adsorption-energy potential landscape, which is likely modified by the influence of the 3rd layer NaCl island step edge. Scale bars correspond to 5 Å.

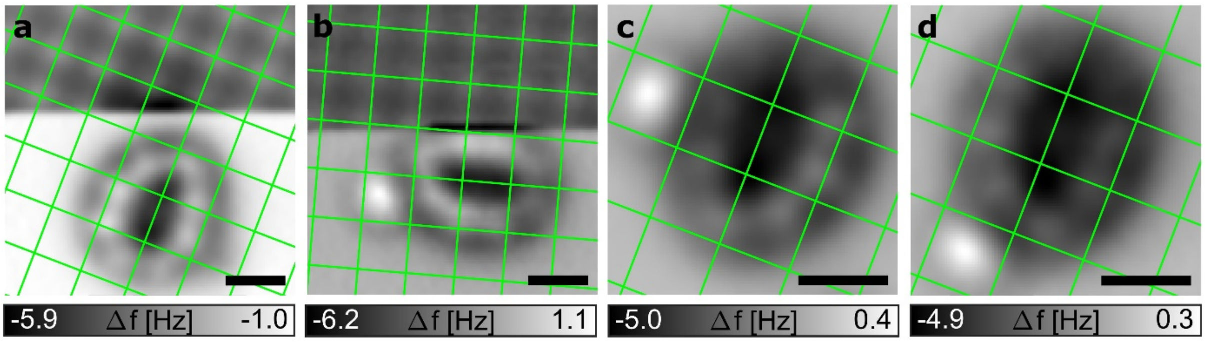

Fig. S15. Experimental adsorption-site determination for C_{16}^- in isolation, i.e., not adjacent to 3rd layer NaCl, on bilayer NaCl. In the upper part of AFM images (a) and (b), the tip was scanned closer to the surface than in the lower parts, where Cl^- ions appear with increased Δf contrast. The green lattice represents the positions of Cl^- ions in the second (top) NaCl layer. For panel c and d, the lattice orientation was determined from separate images and the lattice was aligned to the adsorbed CO molecules (bright features, at Na^+ sites). In all cases, C_{16}^- adsorbs in bridge position with its long axis aligned to rows of Cl^- ions. All scale bars correspond to 5 Å.

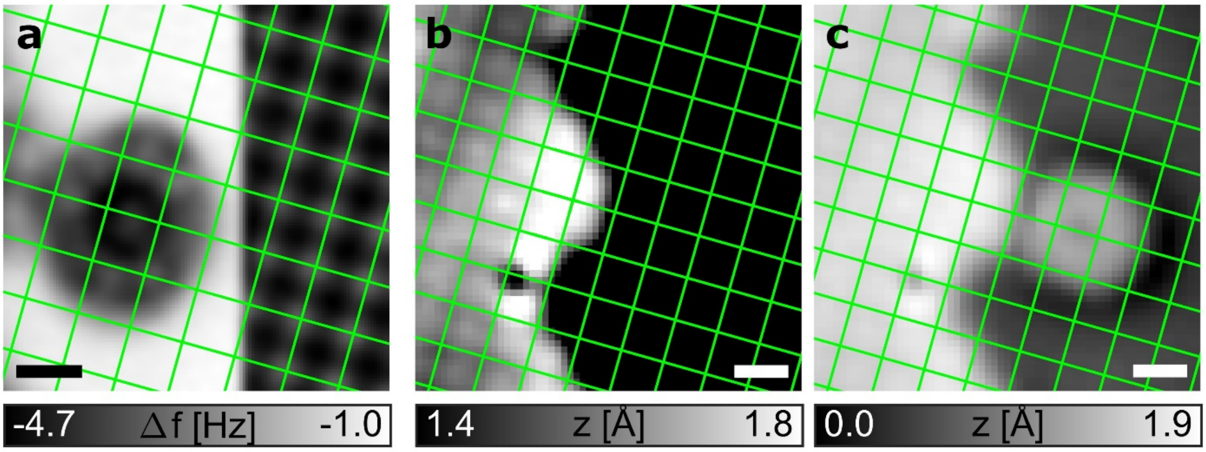

Fig. S16. Experimental adsorption site determination for charge bistable C_{16} shown in Fig. 3f,g. (a) Adsorption site determination for C_{16}^{0} (Fig. 3f) using AFM data. The green lattice represents Cl^{-} anions and was deduced from scanning the tip at a smaller tip-sample distance (right part of the image). Deviating from the most favored adsorption site found in our simulations (see Fig. 3h) this C_{16}^{0} is adsorbed with its center above a bridge position. This might be caused by the 3rd layer NaCl island next to the molecule (at the left of the image). (b) and (c) Adsorption site determination for C_{16}^{-} from STM data. In (b) the z-scale contrast is adjusted to visualize the NaCl lattice on the 3rd layer island. The green lattice represents the positions of Na^{+} ions in the 3rd layer island next to the molecule to the left in the image. These positions correspond to Cl^{-} ions in the NaCl layer below the molecule. (c) The same data and lattice with differently adjusted *z*-scale contrast to visualize the C_{16}^{-} molecule. Here, as in most cases, C_{16}^{-} adsorbed with its long axis along rows of Cl^{-} anions with its center at a bridge position, in agreement with the favored adsorption site and geometry of C_{16}^{-} found by our simulations (see Fig. 3i and Table S1). All scale bars 5 Å.

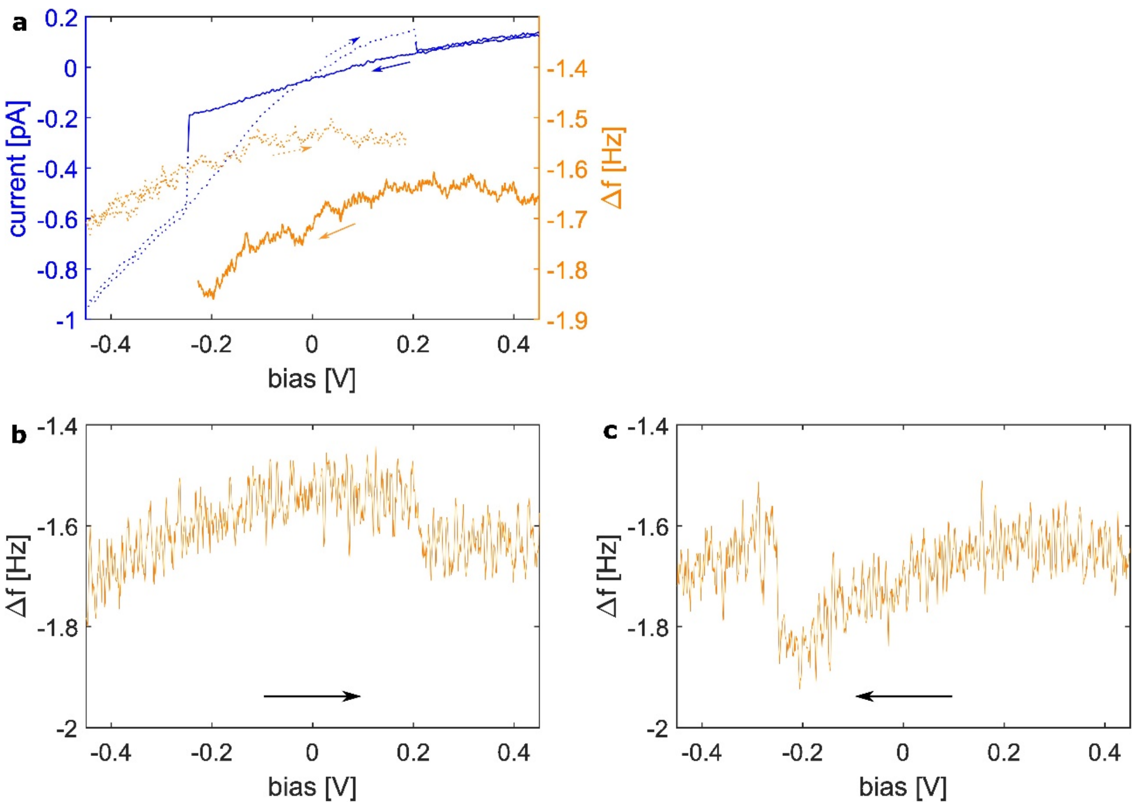

Fig. S17. (a) STS and Kelvin probe force spectroscopy (KPFS) of charge-state switching of C_{16} displayed in Fig. 3f, g. Arrows indicate the direction of the voltage sweep. Blue, the tunnelling current *I*, with the solid (dashed) line indicating the molecule being negatively charged, C_{16}^{-} (neutral, C_{16}^{0}). Orange, the simultaneously recorded KPFS data, i.e., $\Delta f(V)$ spectroscopy, with applied low-pass filter, and only showing the bias region until the charge transition for each graph. The peak of the solid KPFM parabola is shifted to more positive *V* values indicating a more negative charge state of the adsorbate with respect to the dashed KPFS parabola. (b, c) Raw KPFM data of the upward (b) and downward (c) bias sweep, that are shown low-pass filtered and only for the region until the charge transitions in (a).

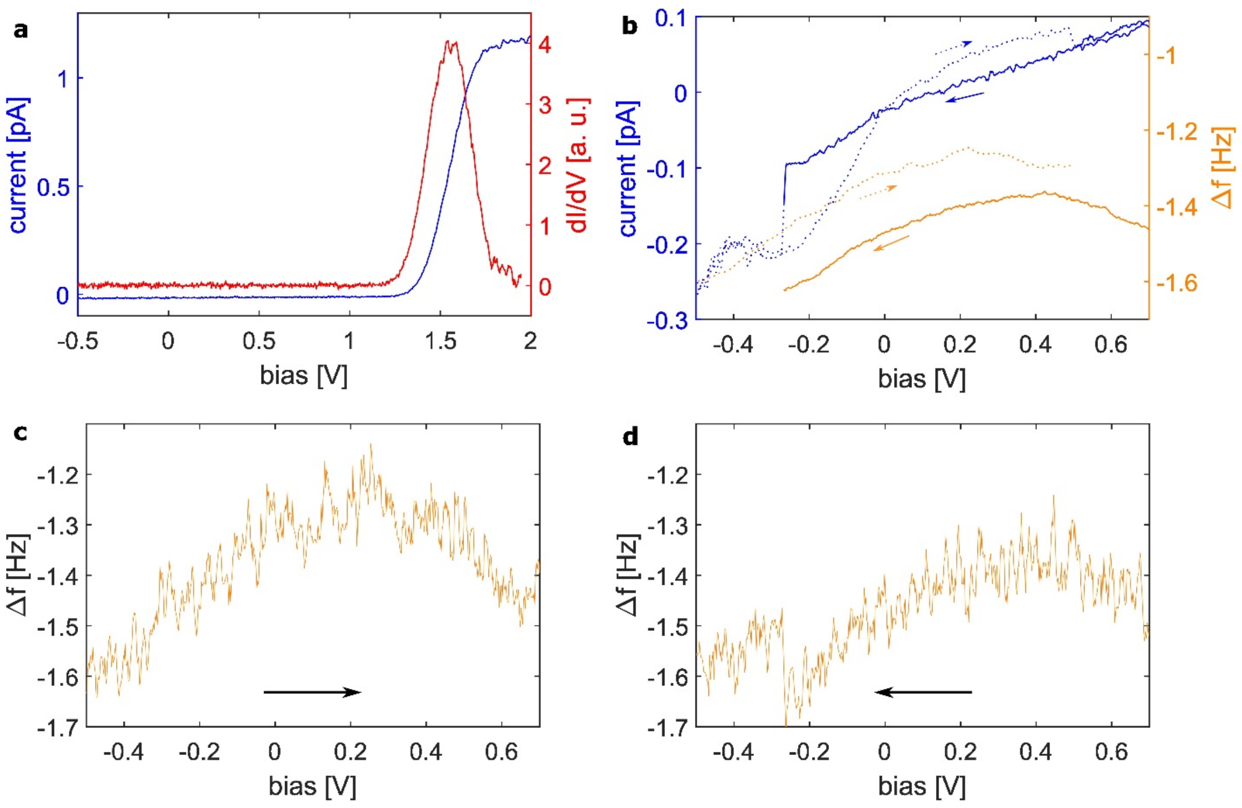

Fig. S18. Spectroscopy data on C_{16}. On the molecule shown in Fig. 4, Fig. S10 and Fig. S12. (a) Scanning tunnelling spectroscopy (STS), $I(V)$ spectrum (blue) recorded on top of C_{16} and numerically derived differential conductance spectrum (red). The dI/dV peak centered at $V = +1.5$ V indicates an electronic resonance. (b) STS (blue) and KPFS (orange) in the region of reversible charge switching. The direction of the voltage sweep is indicated. Blue, the tunnelling current, with the solid (dashed) line indicating the molecule being negatively charged (neutral). Orange, the simultaneously recorded KPFS data, i.e., $\Delta f(V)$ spectroscopy with applied low-pass filter, and only showing the bias region until the charge transition for each graph. The peak of the solid KPFM parabola is shifted to more positive V values indicating a more negative charge state of the adsorbate with respect to the dashed KPFS parabola. (c, d) Raw KPFM data of the upward (c) and downward (d) bias sweep, that are shown low-pass filtered and only for the region until the charge transitions in (b).

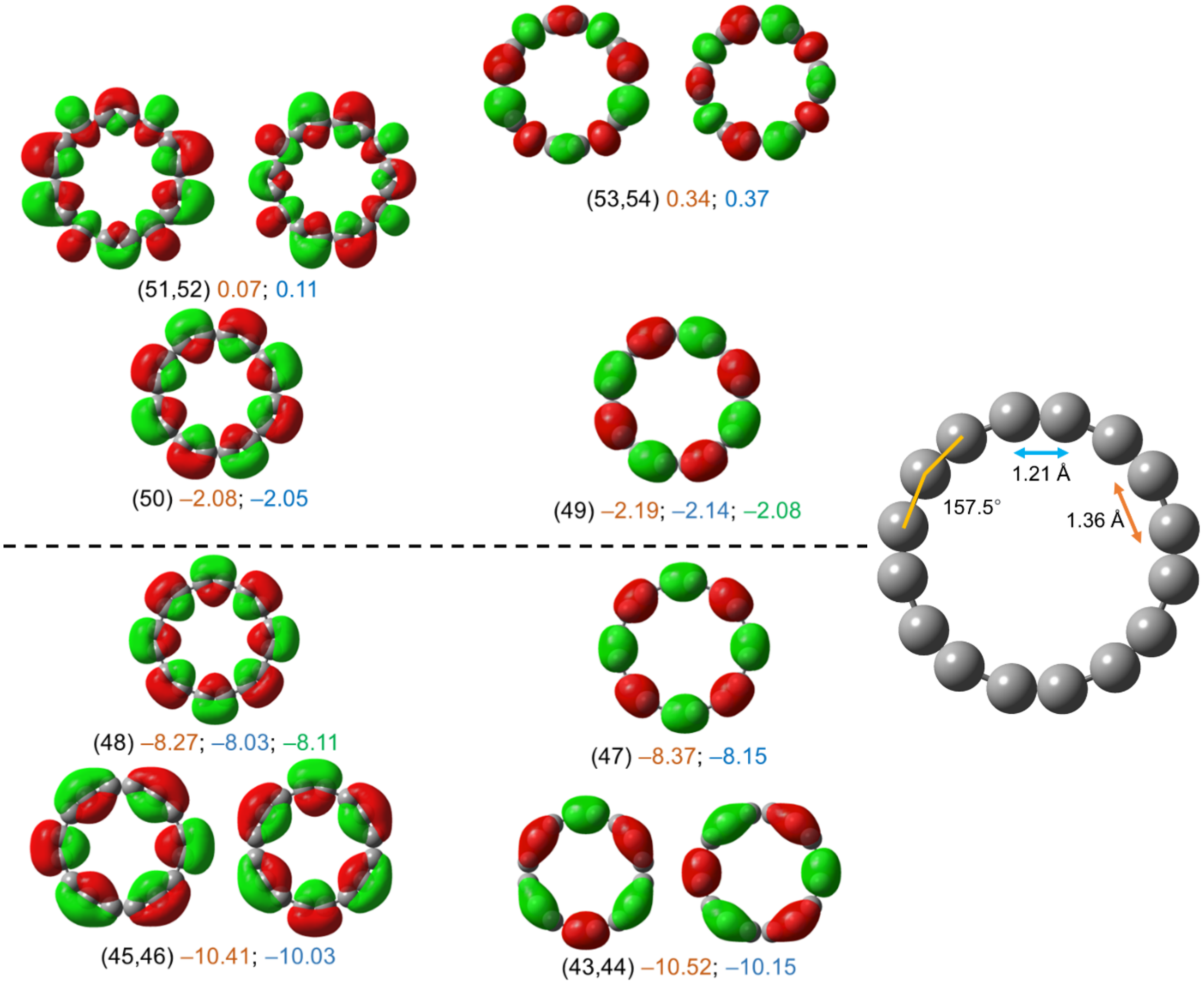

Fig. S19. Left: Molecular orbitals of C_{16}^{0} obtained using ωB97XD/def2-QZVP, with orbital energies (in eV) shown in blue. Electron addition and electron removal energies (in eV) using EOM-CCSD/def2-QZVPP and CASPT2/ANO-L-VTZP are show in orange and green, respectively. The dashed line divides the occupied and unoccupied orbitals. In-plane orbitals including A', B' are shown on the left, and out-of-plane orbitals including A'', B'' (shown in Fig. 1) on the right. Orbital numbers are in parentheses. Right: Geometry of C_{16}^{0} optimized at ωB97XD/def2-TZVP, with bond lengths (blue and orange) and bond angles (yellow) shown.

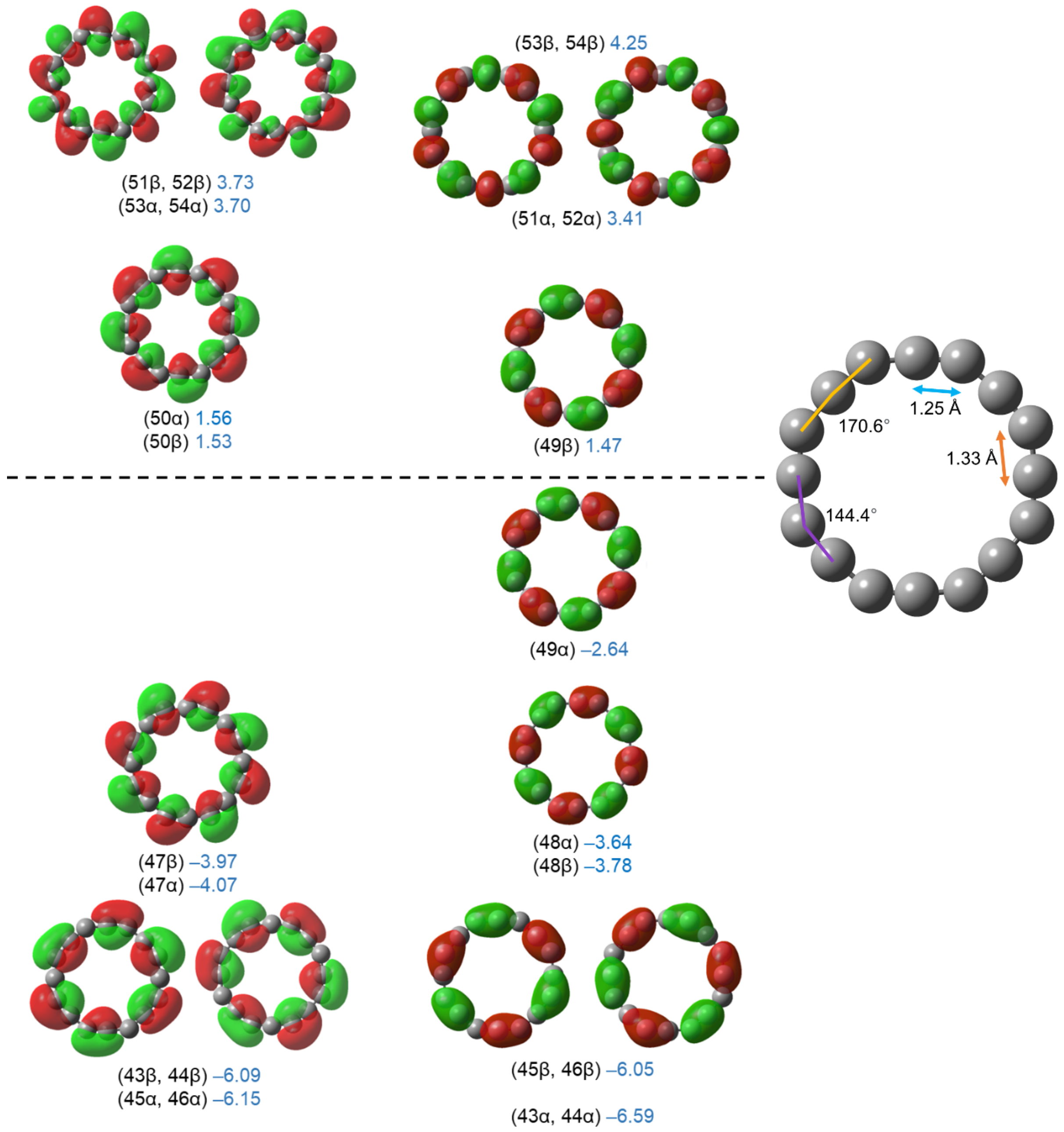

Fig. S20. Left: Orbital energies (in eV) for C_{16}^{-} obtained using ωB97XD/def2-QZVP (blue). The dashed line divides the occupied and unoccupied orbitals. In-plane orbitals are shown on the left, and out-of-plane orbitals on the right. Orbital numbers are in parentheses. Right: Geometry of C_{16}^{-} optimized at ωB97XD/def2-TZVP, with bond lengths (blue and orange) and bond angles (yellow and purple) shown.

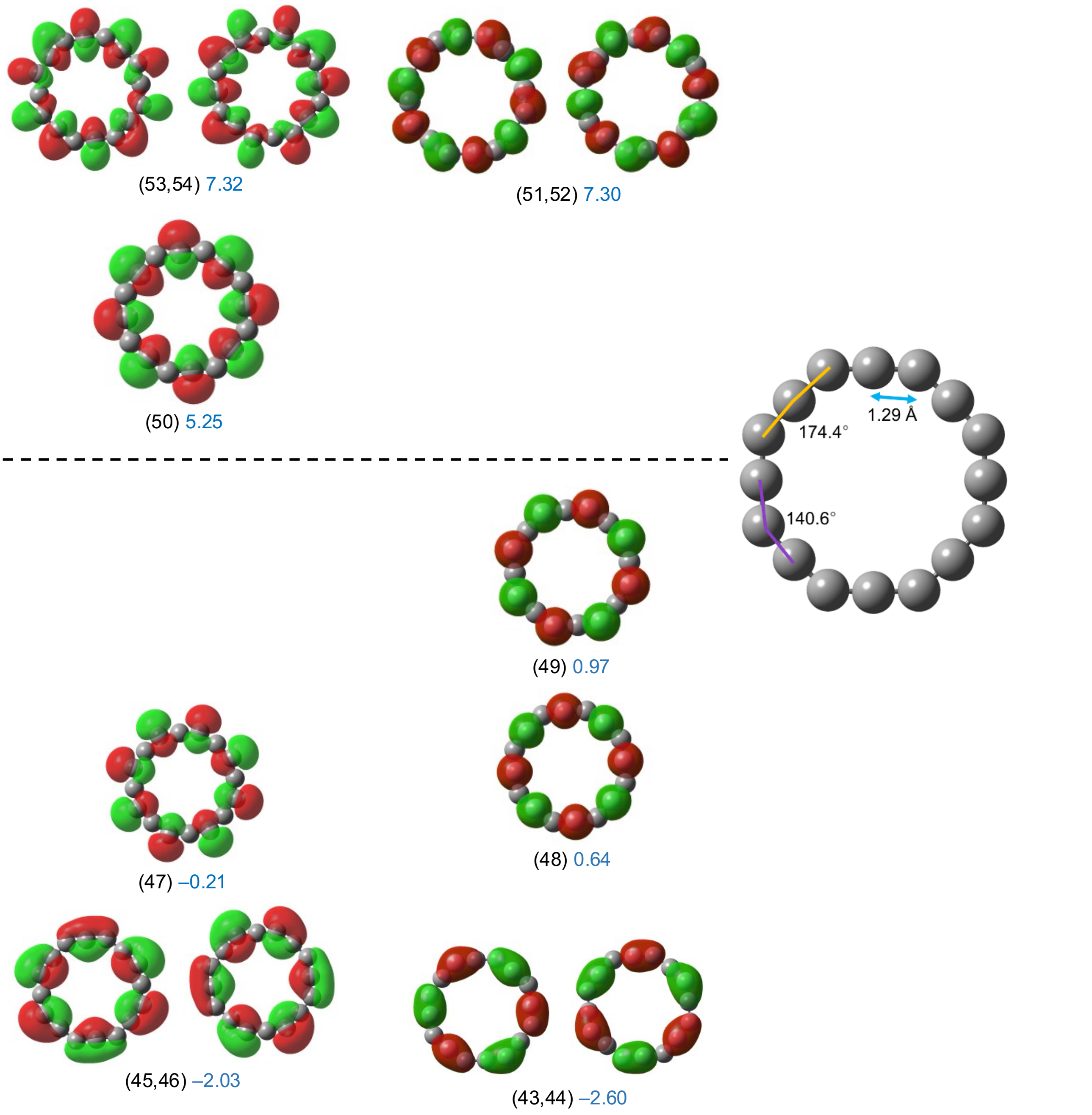

Fig. S21. Left: Orbital energies (in eV) for C_{16}^{2-} obtained using ωB97XD/def2-QZVP (blue). The dashed line divides the occupied and unoccupied orbitals. In-plane orbitals are shown on the left, and out-of-plane orbitals on the right. Orbital numbers are in parentheses. Right: Geometry of C_{16}^{2-} optimized at ωB97XD/def2-TZVP, with bond lengths (blue) and bond angles (yellow and purple) shown.

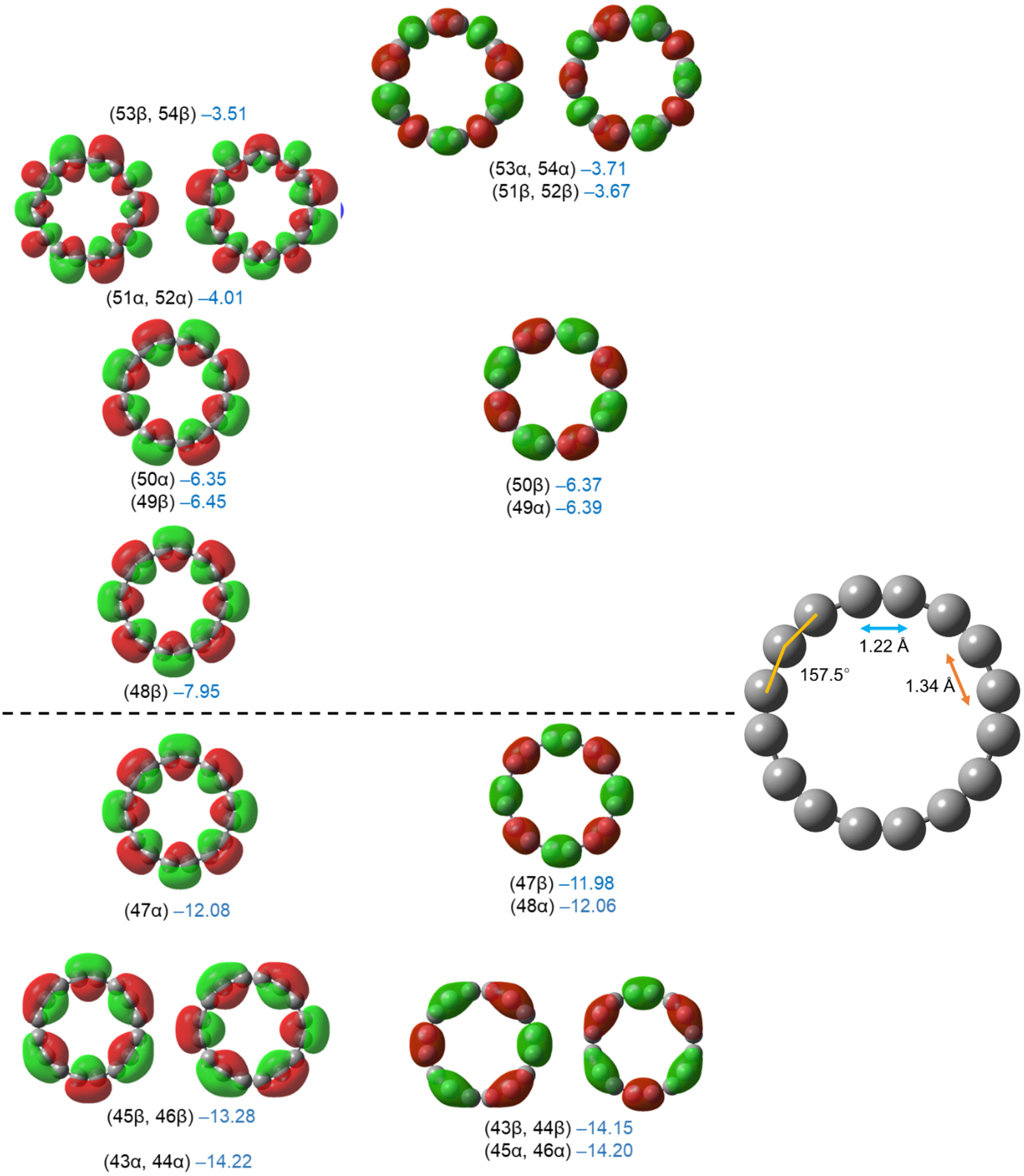

Fig. S22. Left: Orbital energies (in eV) for C_{16}^{+} obtained using ωB97XD/def2-QZVP (blue). The dashed line divides the occupied and unoccupied orbitals. In-plane orbitals are shown on the left, and out-of-plane orbitals on the right. Orbital numbers are in parentheses. Right: Geometry of C_{16}^{+} optimized at ωB97XD/def2-TZVP, with bond lengths (blue and orange) and bond angles (yellow) shown.

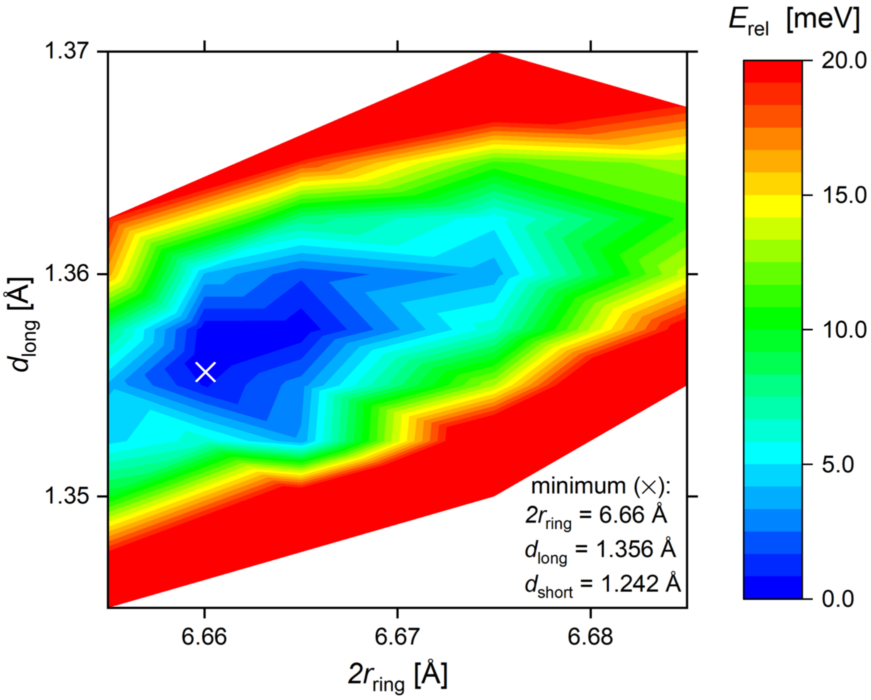

Fig. S23. Grid optimization of C_{16}^{0} at the NEVPT2 level of theory, with the longer C-C bond (d_{long}) and ring diameter ($2r_{ring}$) as variables. The minimum geometry position is marked with a white cross.

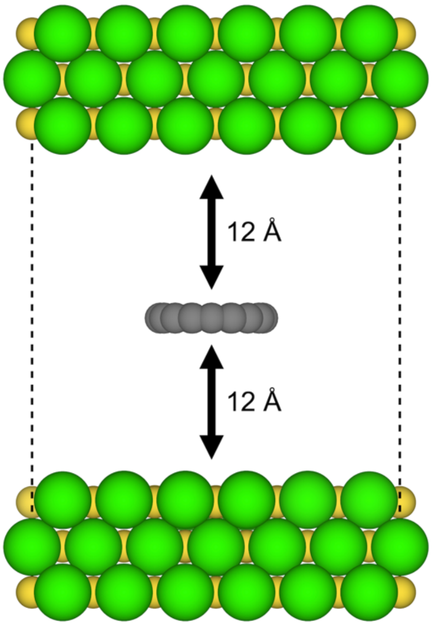

Fig. S24. The reference geometry used for determining the C_{16}@NaCl adsorption energy.

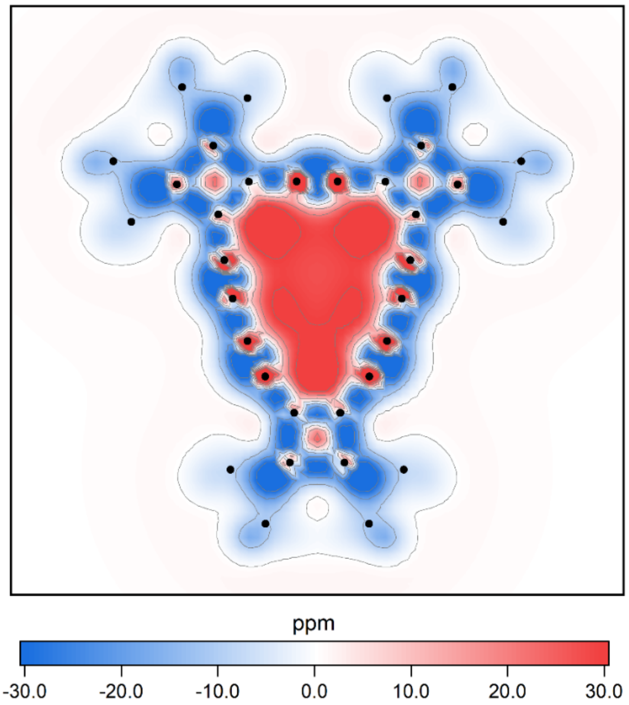

Fig. S25. NICS(0)$_{zz}$ values for **S8**, calculated at the ωB97XD/def2-TZVP level of theory. Carbon atoms are shown as black dots.

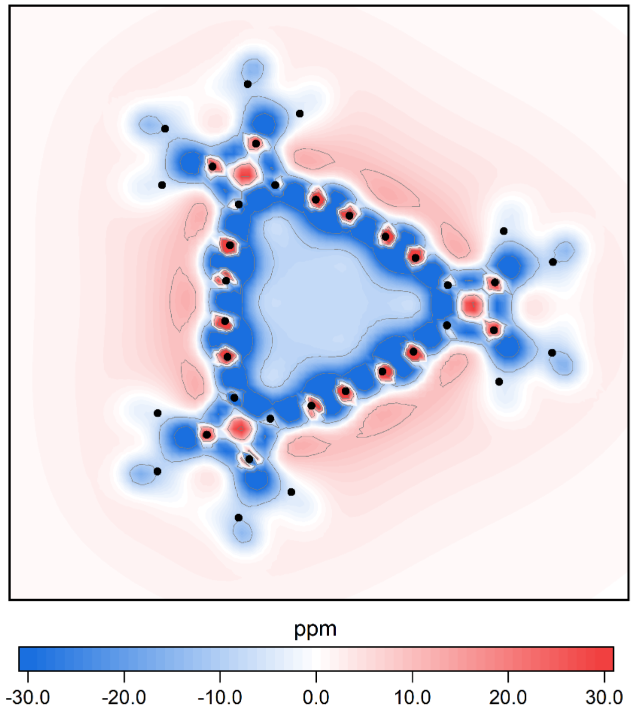

Fig. S26. NICS(0)$_{zz}$ values for **S10**, calculated at the ωB97XD/def2-TZVP level of theory. Carbon atoms are shown as black dots.

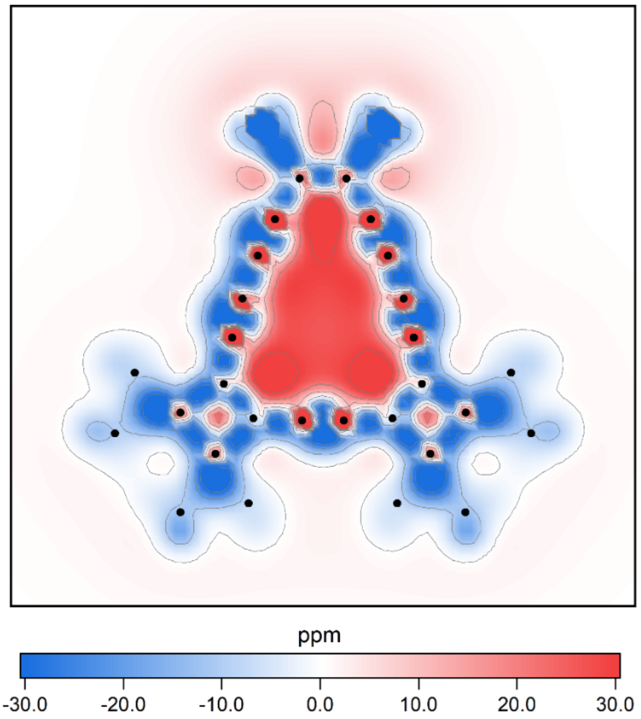

Fig. S27. NICS(0)$_{zz}$ values for **3**, calculated at the ωB97XD/def2-TZVP level of theory. Carbon atoms are shown as black dots.

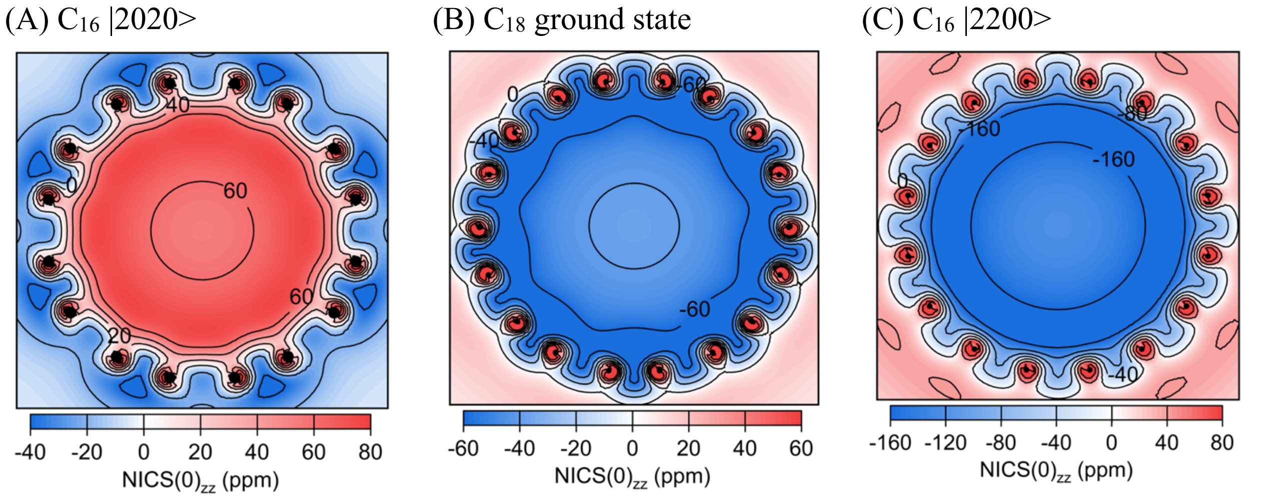

Fig. S28. NICS(0)$_{zz}$ plots for the (A) C_{16} |2020> configuration, (B) C_{18}, and (C) C_{16} |2200> configuration, calculated at the ωB97XD/def2-TZVP level of theory. Carbon atoms are shown as black dots. In the C_{16} |1111> configuration, the paratropic current induced by the coupling between the singly occupied A_4 and B_4 orbitals is not well-defined, as it is (within first-order perturbation theory and a single-reference framework) dependent on the orbital energy difference, which approaches zero at BLA = 0.[33]

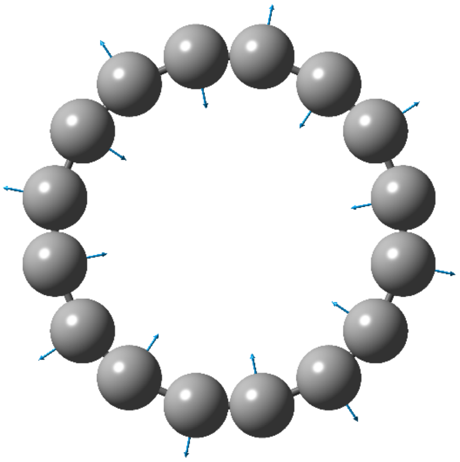

Fig. S29. The unstable ($k < 0$) vibrational frequency of C_{16}^{-} in D_{8h} symmetry, leading to its distortion into C_{8h}.

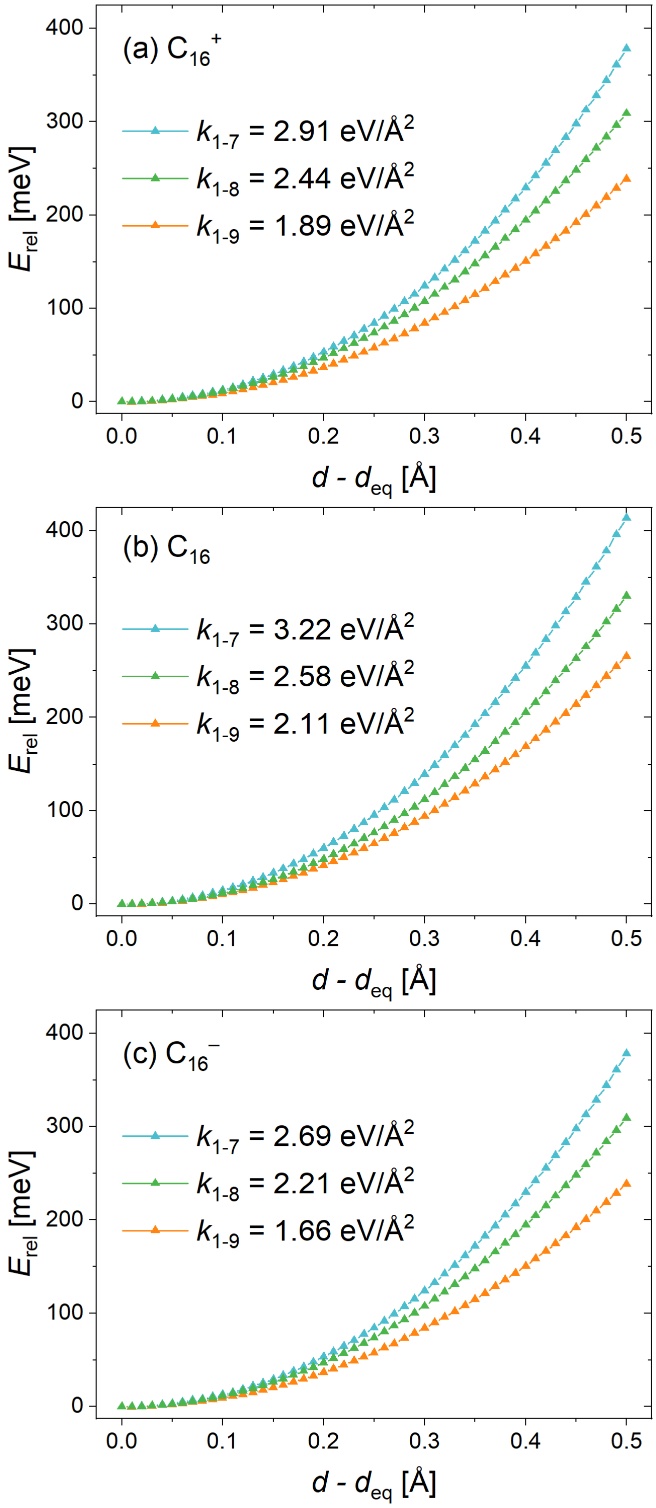

Fig. S30. Ring deformation curves for small displacements and their associated force constants *k* (fit to $E_{\mathrm{rel}} = \frac{1}{2}\, k\, (\Delta x)^2$) for C_{16}^{+} (a), C_{16}^{0} (b), and C_{16}^{-} (c) along the three longest ring diagonals (denoted 1-9, 1-8, and 1-7), obtained at the ωB97XD/def2-TZVP level of theory by performing a relaxed scan.

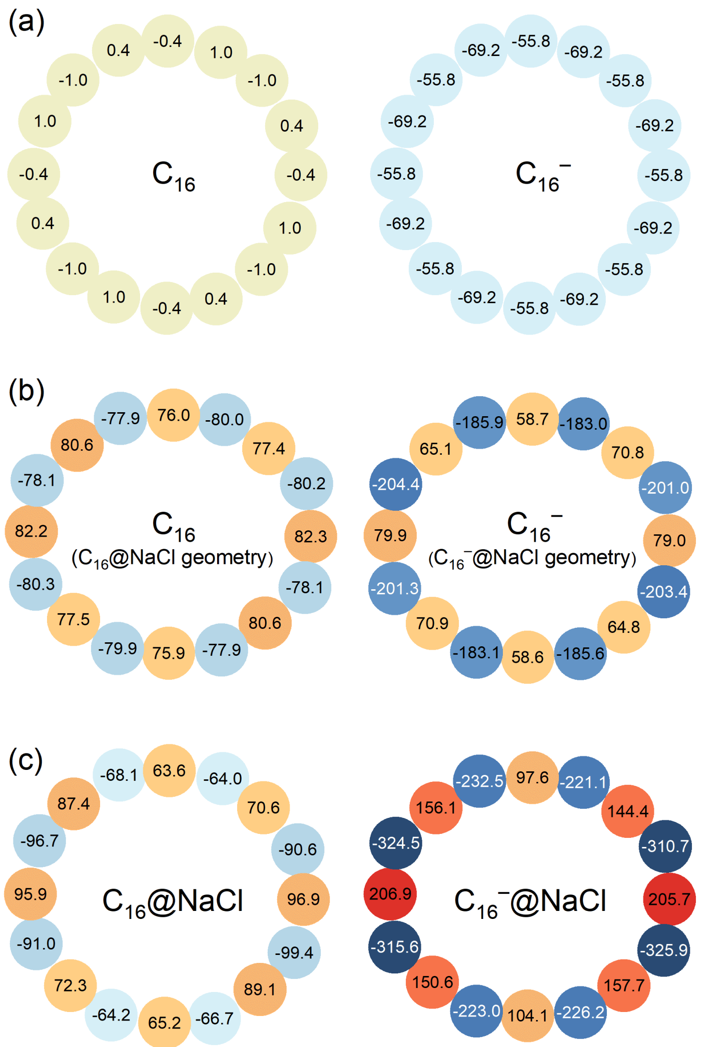

Fig. S31. NBO charges (in 10^{-3} *e*) of (a) gas-phase PBE-optimized C_{16}^{0} and C_{16}^{-}. (b) PBE-optimized geometries of C_{16}^{0} and C_{16}^{-} adsorbed at the bridge site, and (c) PBE-optimized geometries of C_{16}^{0} and C_{16}^{-} adsorbed at the bridge site with a cluster model of NaCl.

NMR Spectra:

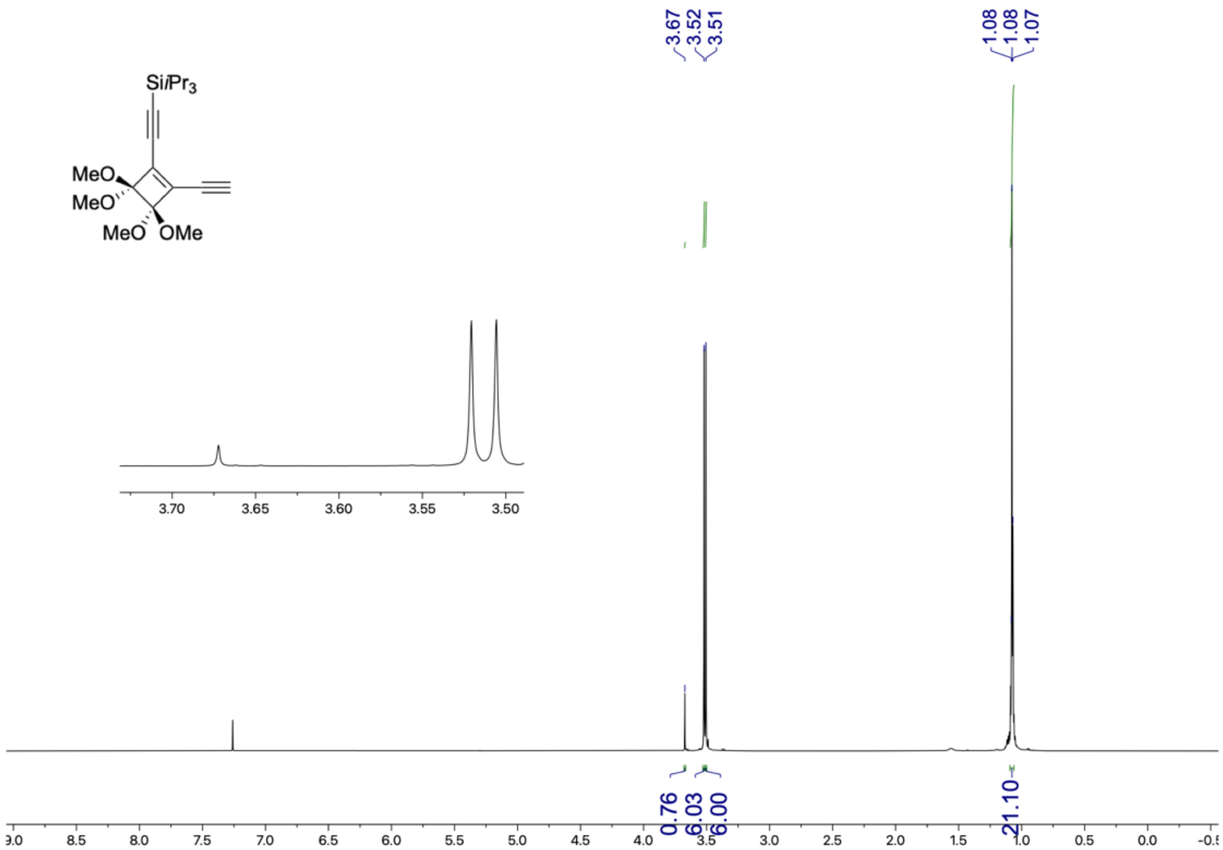

Fig. S32. ^{1}H NMR (400 MHz) spectrum of compound **S2** in $CDCl_3$.

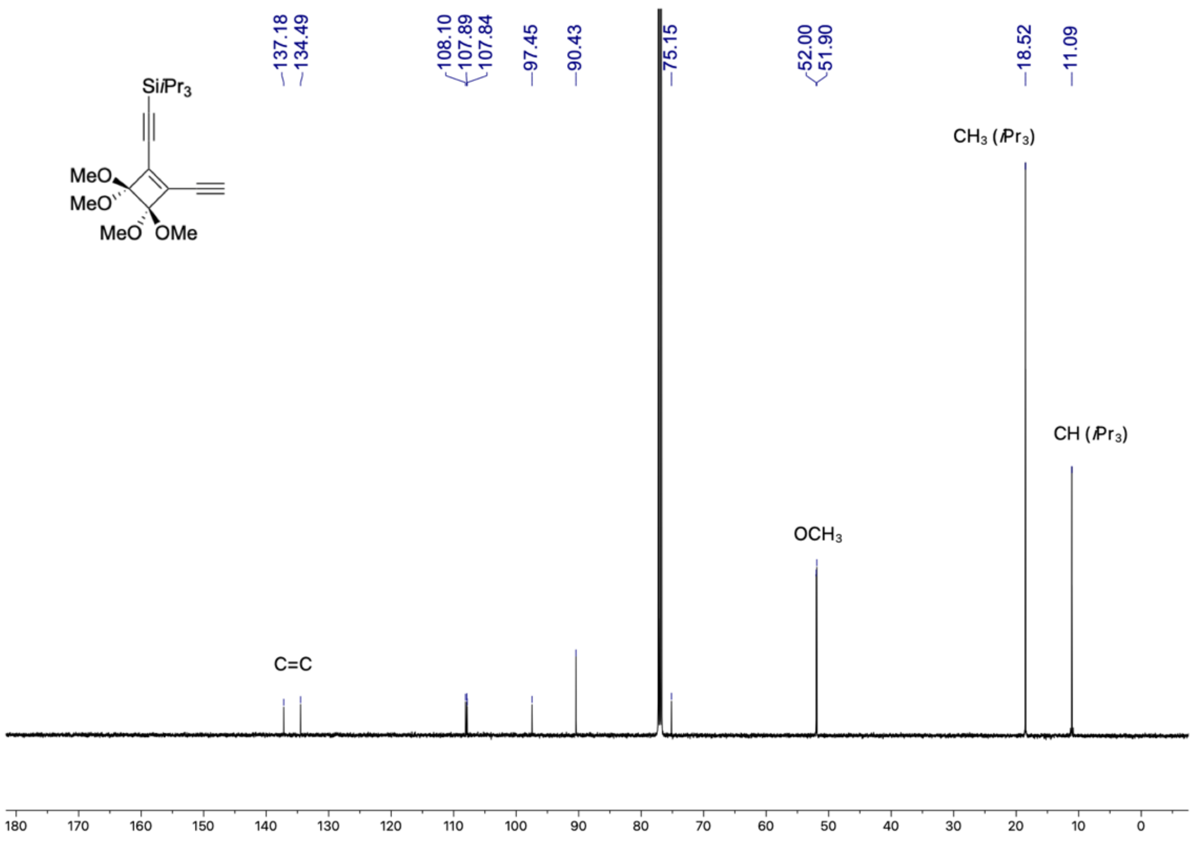

Fig. S33. ^{13}C NMR (101 MHz) spectrum of compound **S2** in $CDCl_3$.

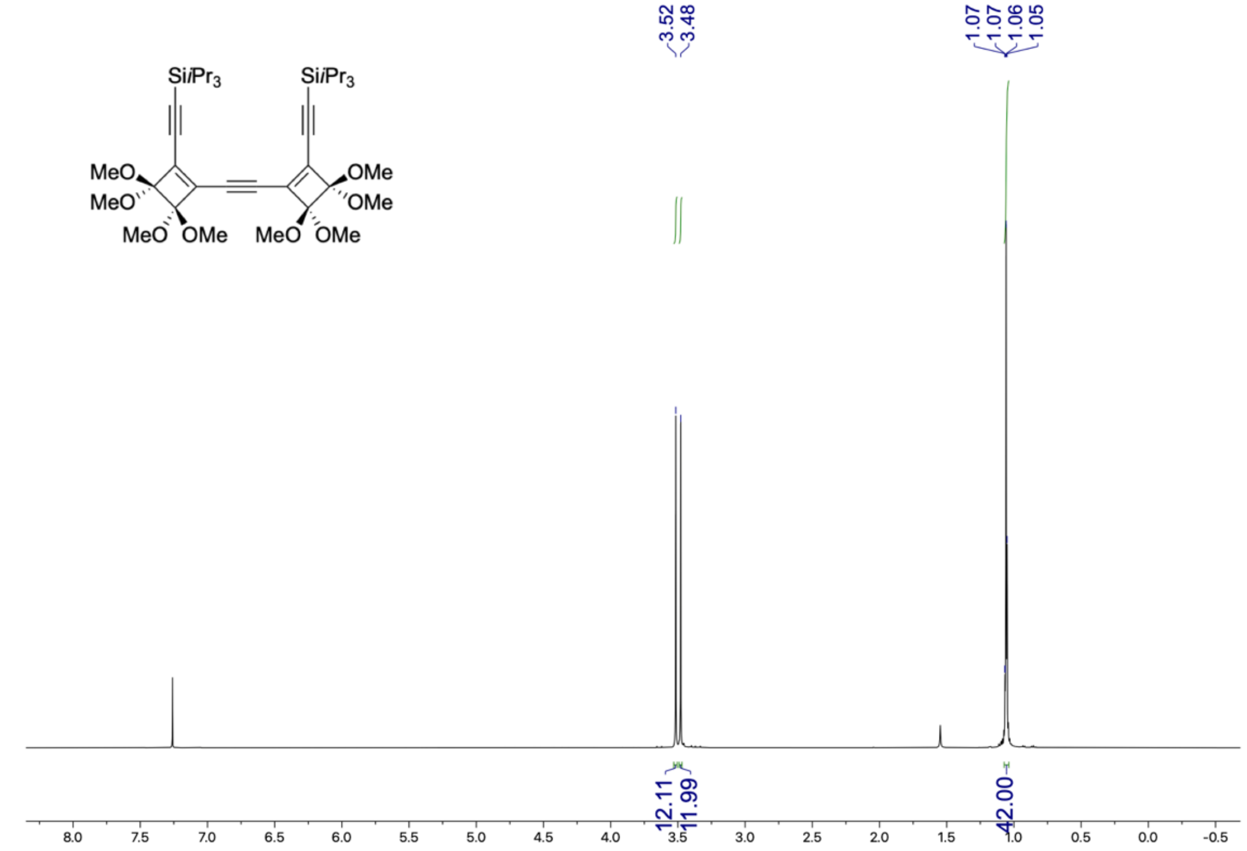

Fig. S34. ^{1}H NMR (500 MHz) spectrum of compound **S4** in $CDCl_3$.

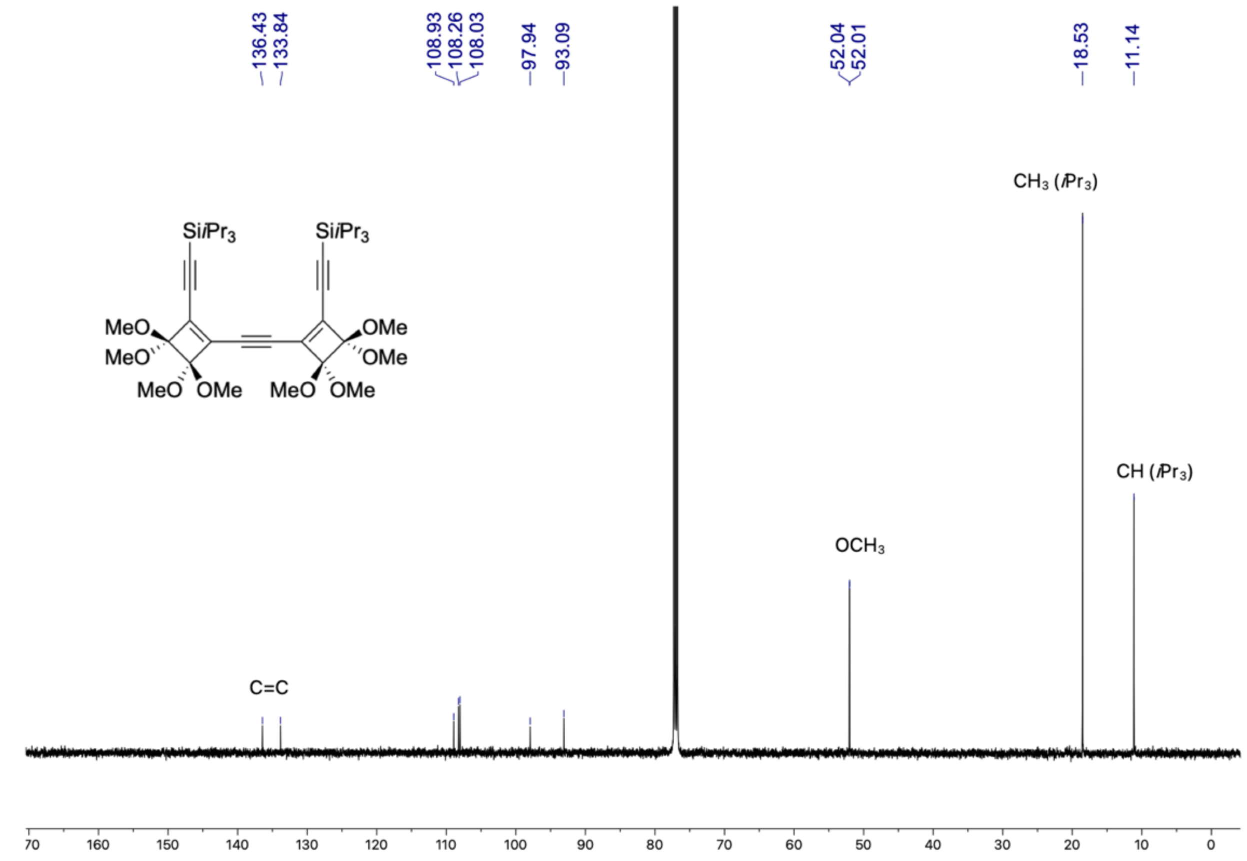

Fig. S35. ^{13}C NMR (126 MHz) spectrum of compound **S4** in $CDCl_3$.

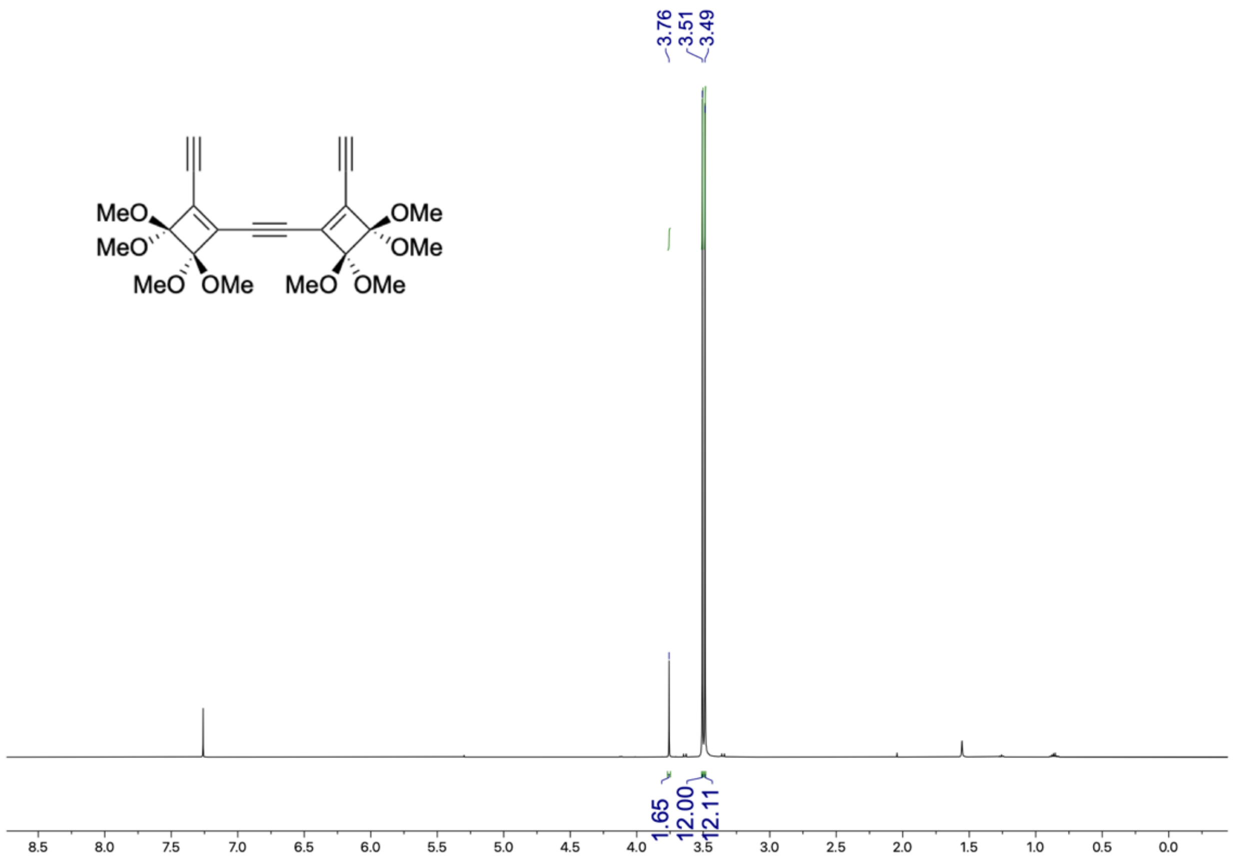

Fig. S36. ^{1}H NMR (500 MHz) spectrum of compound **1** in $CDCl_3$.

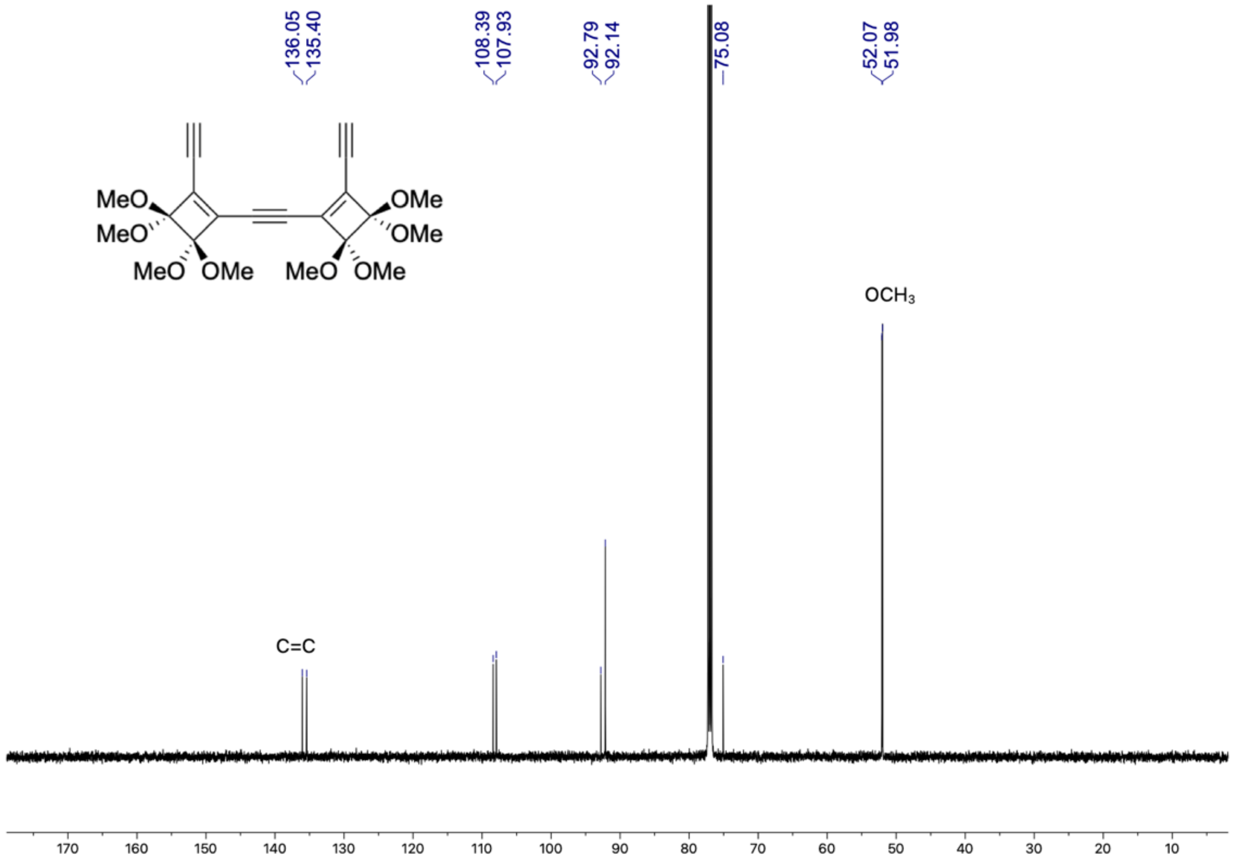

Fig. S37. ^{13}C NMR (126 MHz) spectrum of compound **1** in $CDCl_3$.

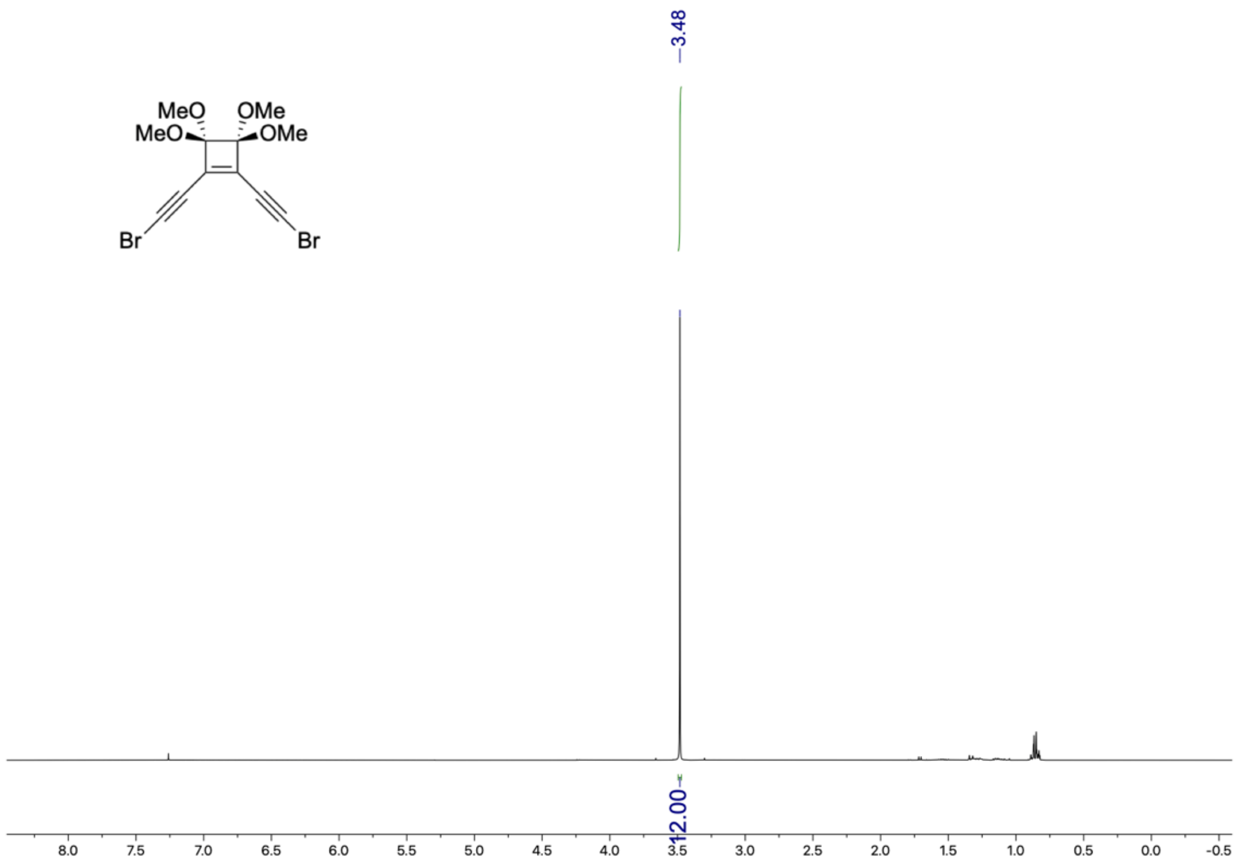

Fig. S38. ^{1}H NMR (500 MHz) spectrum of compound **S6** in $CDCl_3$.

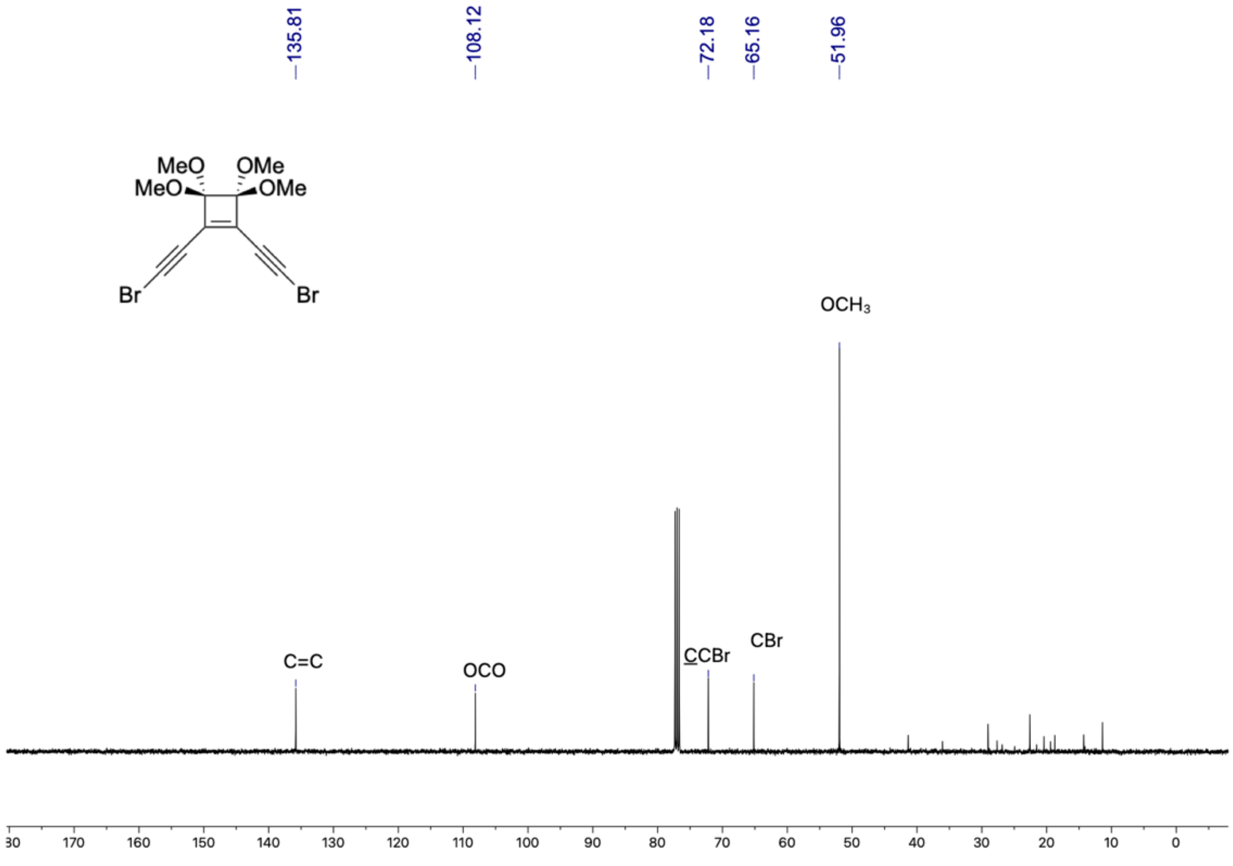

Fig. S39. ^{13}C NMR (126 MHz) spectrum of compound **S6** in $CDCl_3$.

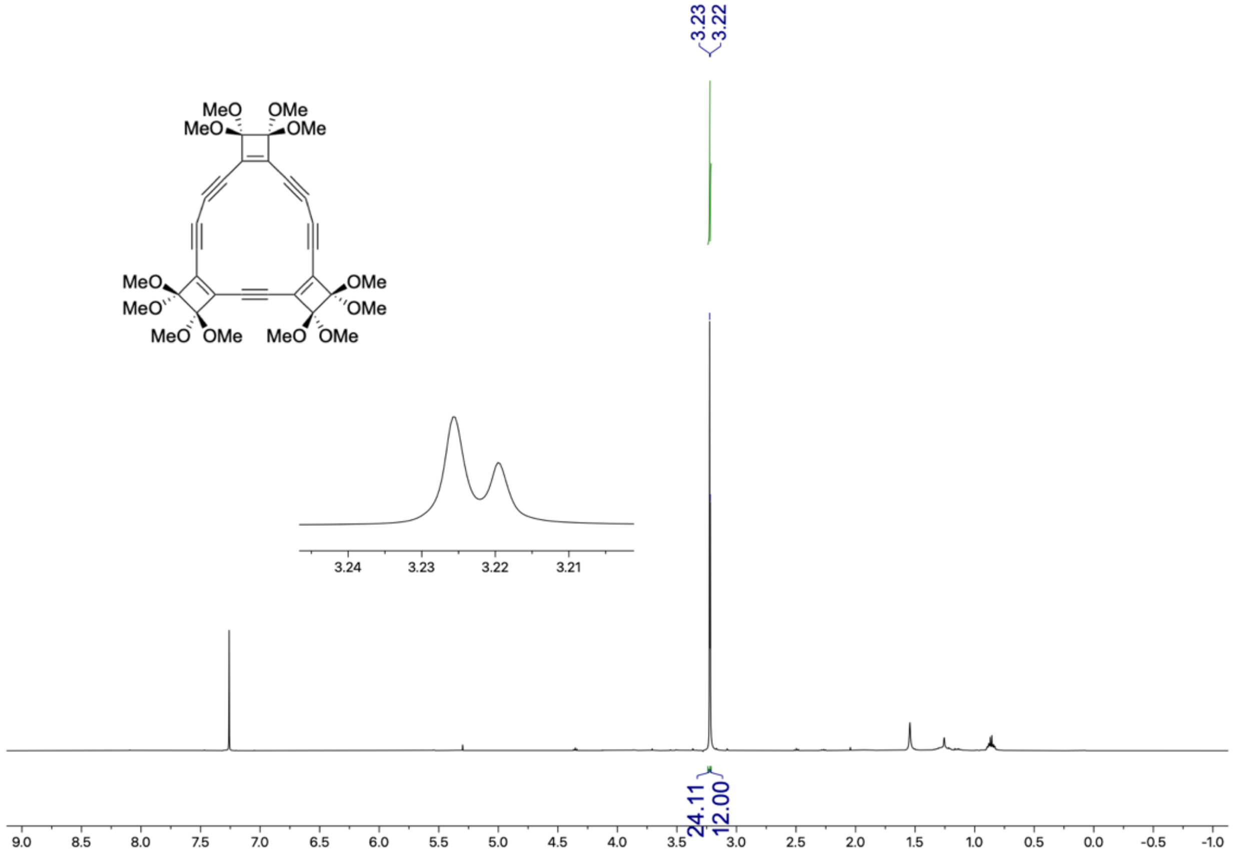

Fig. S40. ^{1}H NMR (500 MHz) spectrum of compound **S8** in $CDCl_3$.

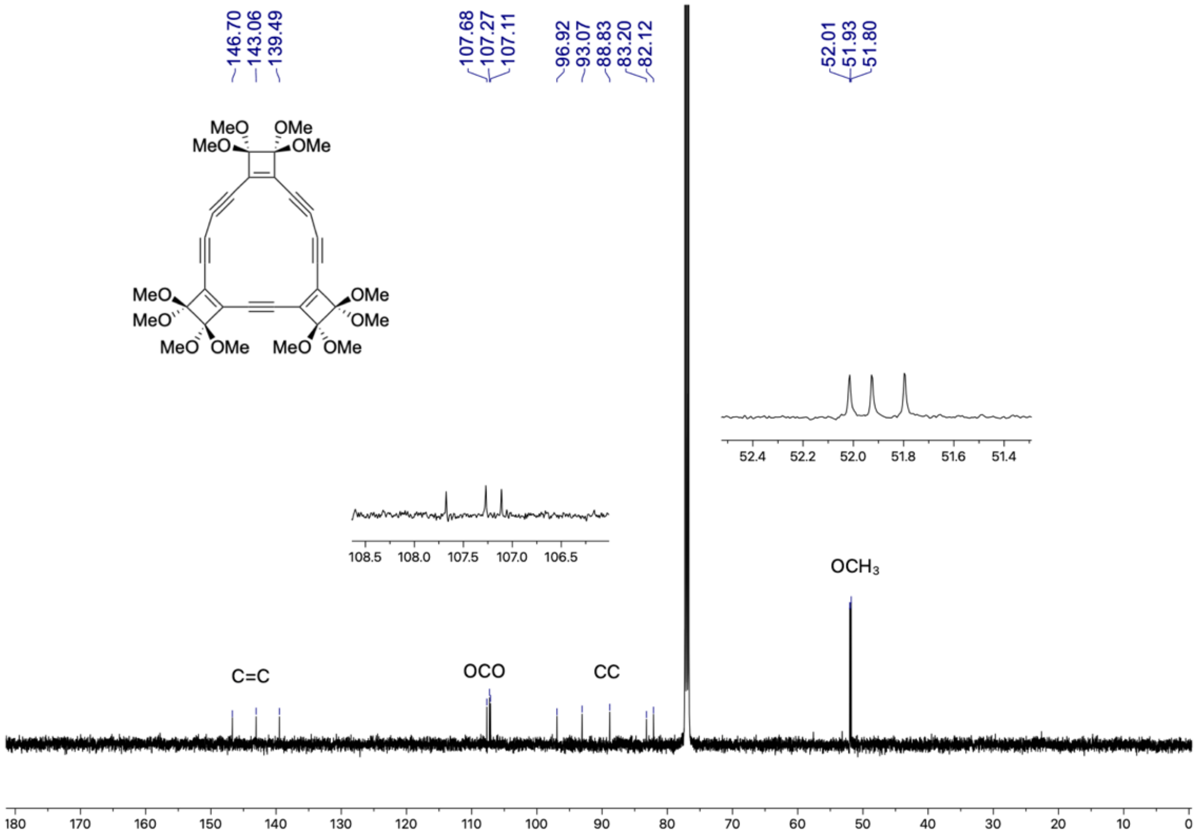

Fig. S41. ^{13}C NMR (126 MHz) spectrum of compound **S8** in $CDCl_3$.

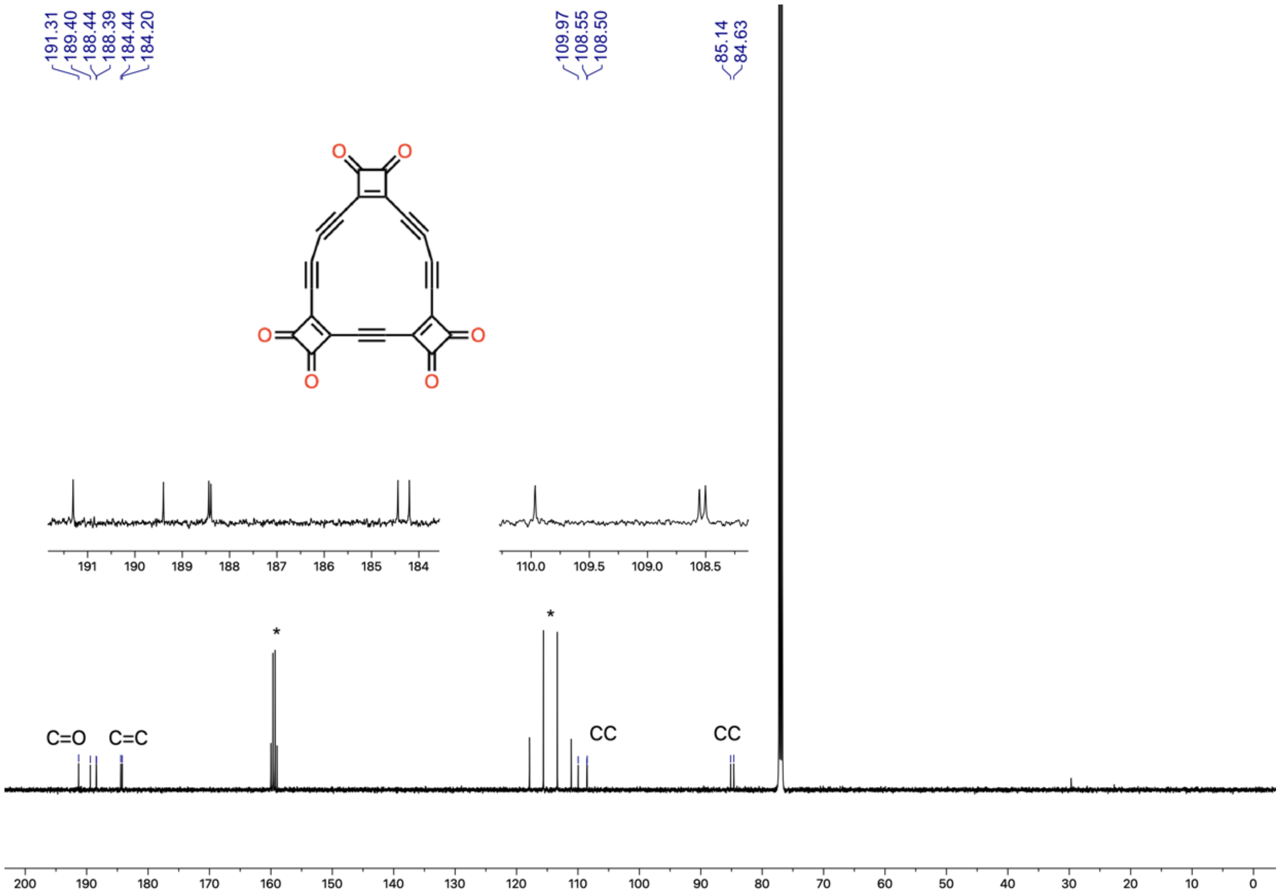

Fig. S42. ^{13}C NMR (126 MHz, 6500 scans) spectrum of compound **S12** in $CDCl_3$. *Denotes the residual TFA solvent.

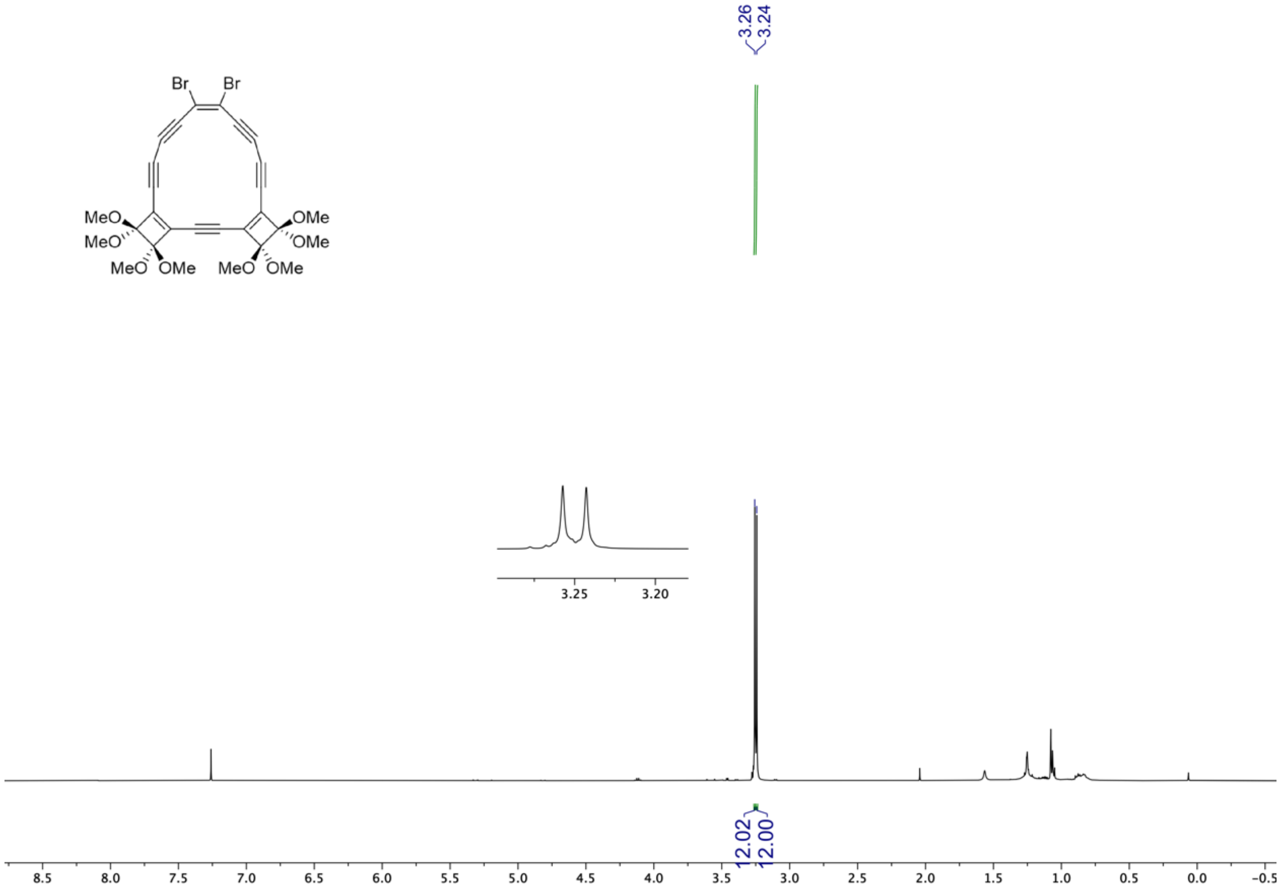

Fig. S43. ^{1}H NMR (500 MHz) spectrum of compound **3** in $CDCl_3$.

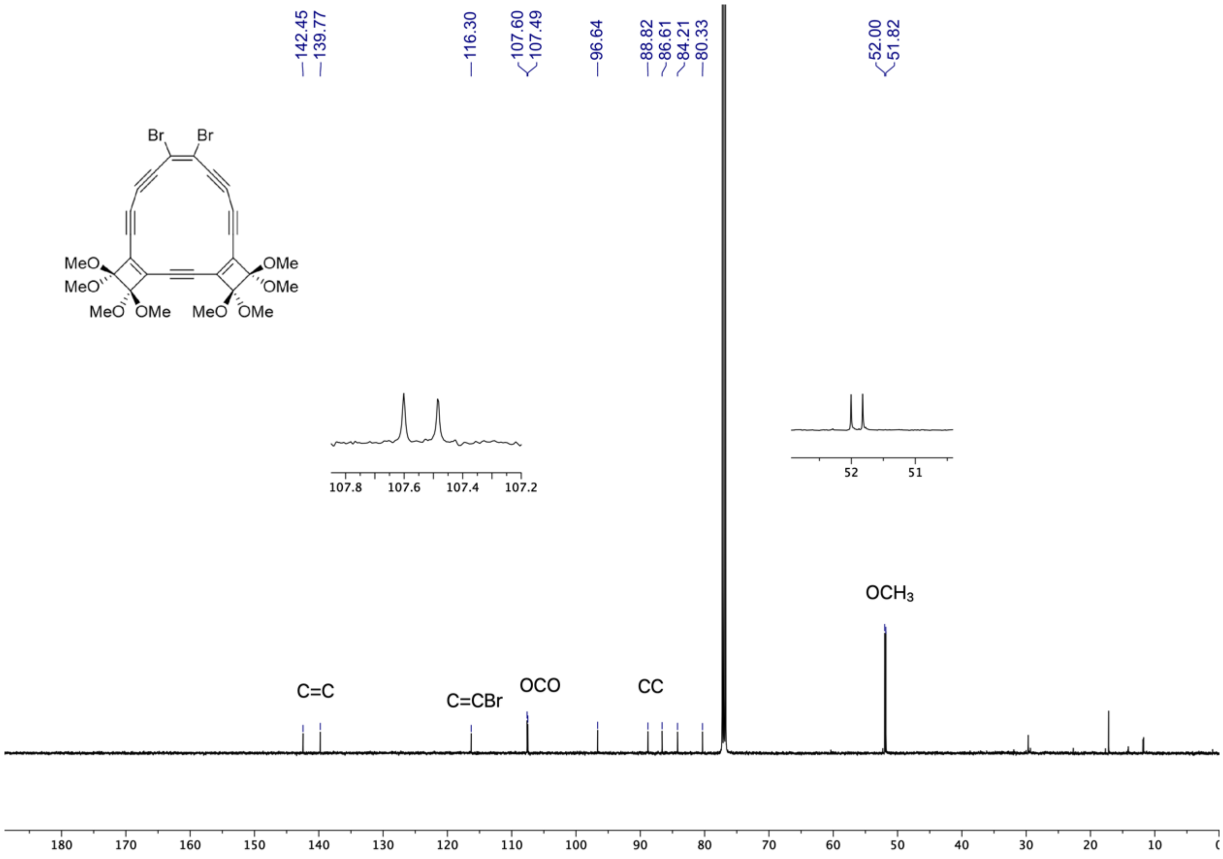

Fig. S44. ^{13}C NMR (126 MHz) spectrum of compound **3** in $CDCl_3$.

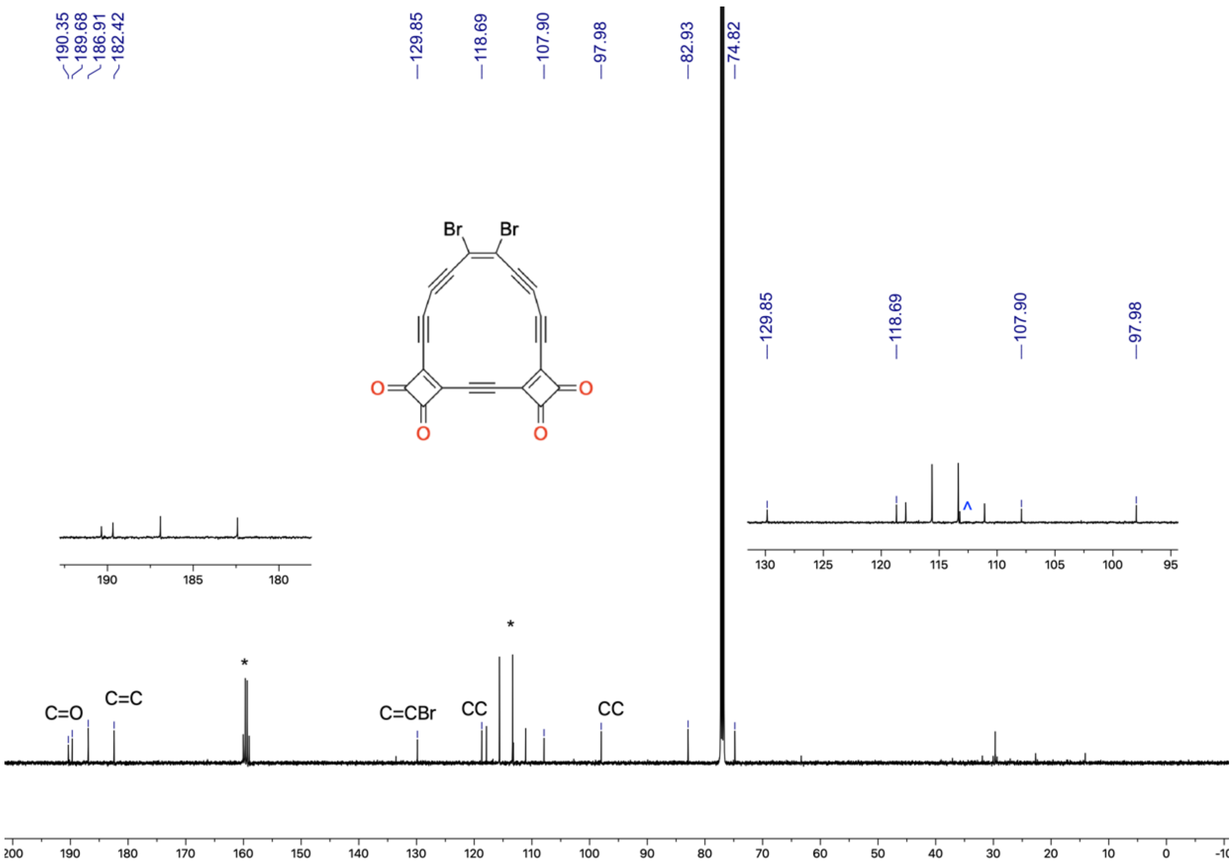

Fig. S45. ^{13}C NMR (126 MHz, 6500 scans) spectrum of compound **4** in $CDCl_3$. * and ^ denote the residual TFA solvent and an unidentified signal, respectively.

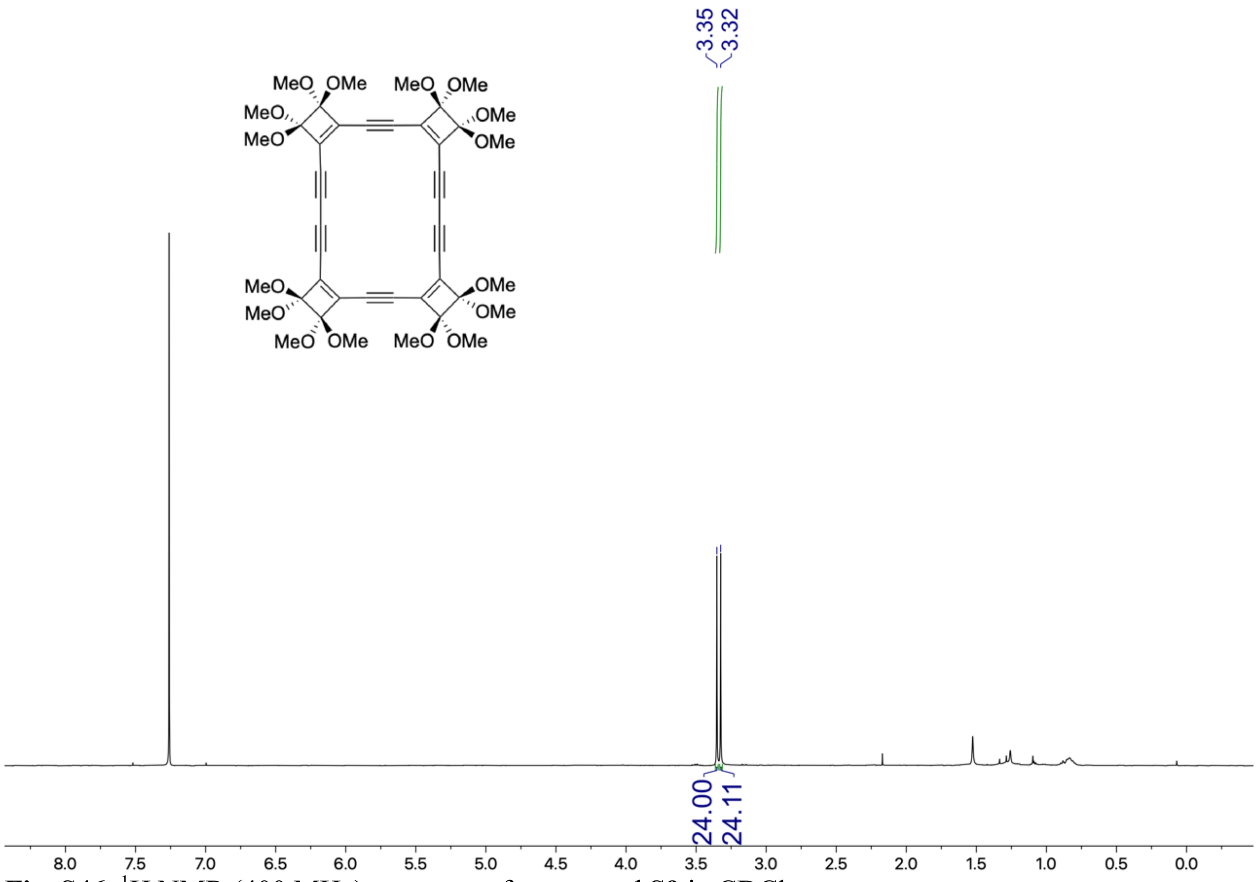

Fig. S46. ^{1}H NMR (400 MHz) spectrum of compound **S9** in $CDCl_3$.

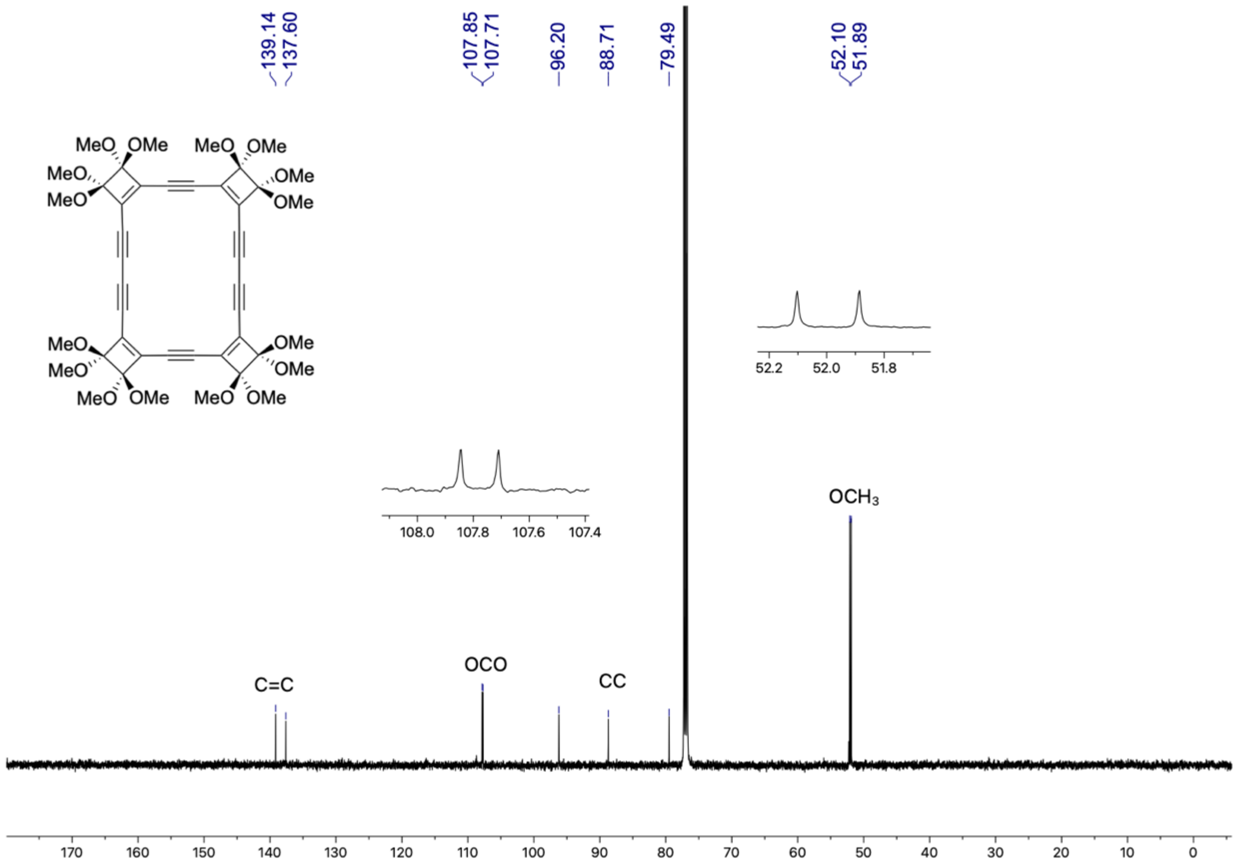

Fig. S47. ^{13}C NMR (101 MHz) spectrum of compound **S9** in $CDCl_3$.

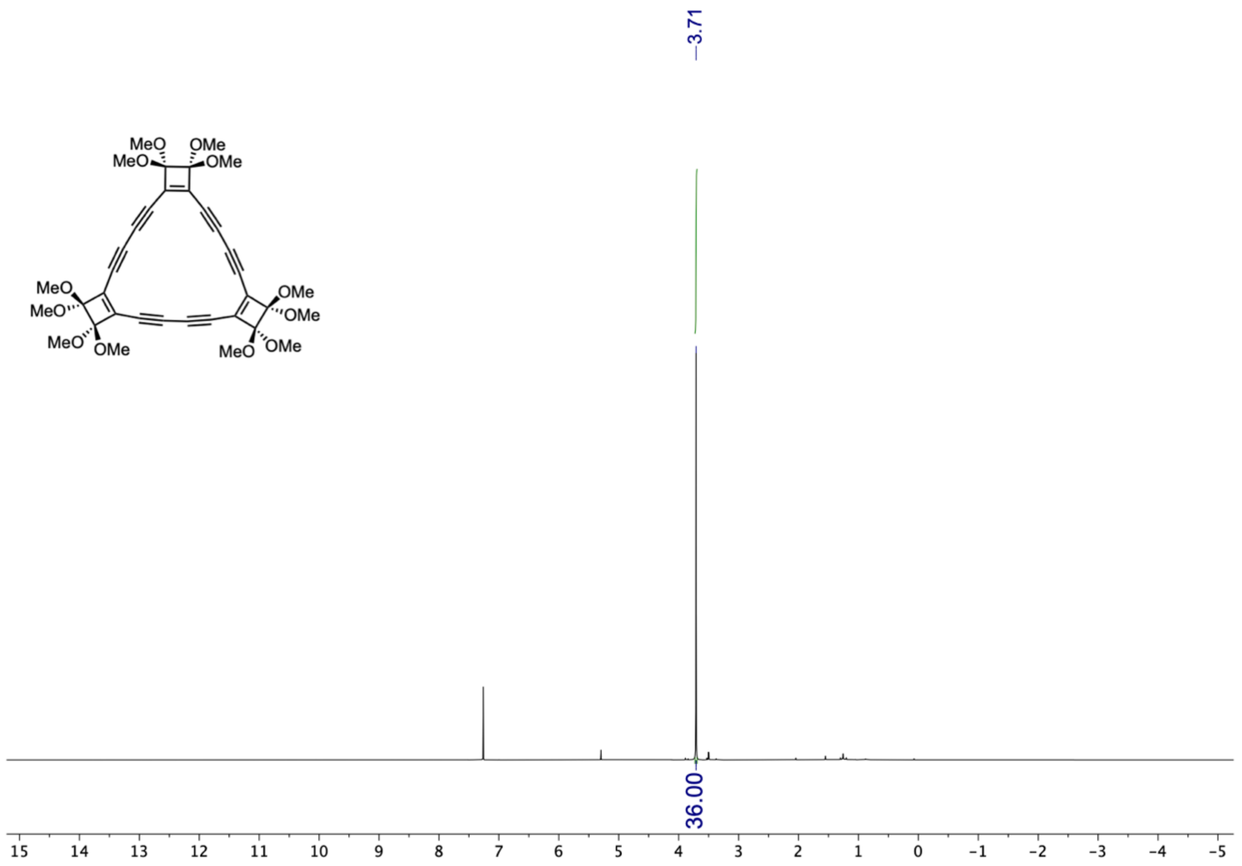

Fig. S48. ^{1}H NMR (400 MHz) spectrum of compound **S10** in $CDCl_3$.

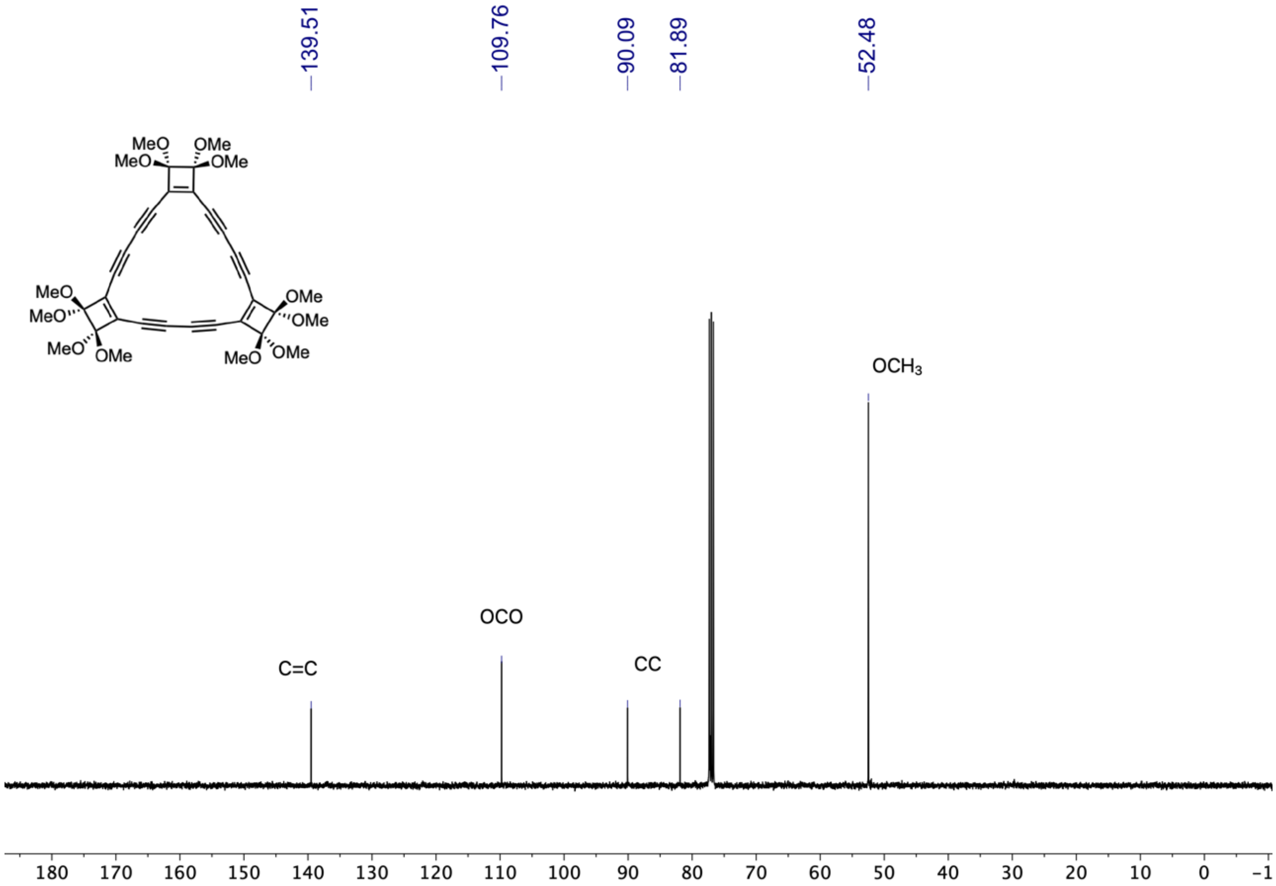

Fig. S49. ^{13}C NMR (126 MHz) spectrum of compound **S10** in $CDCl_3$.

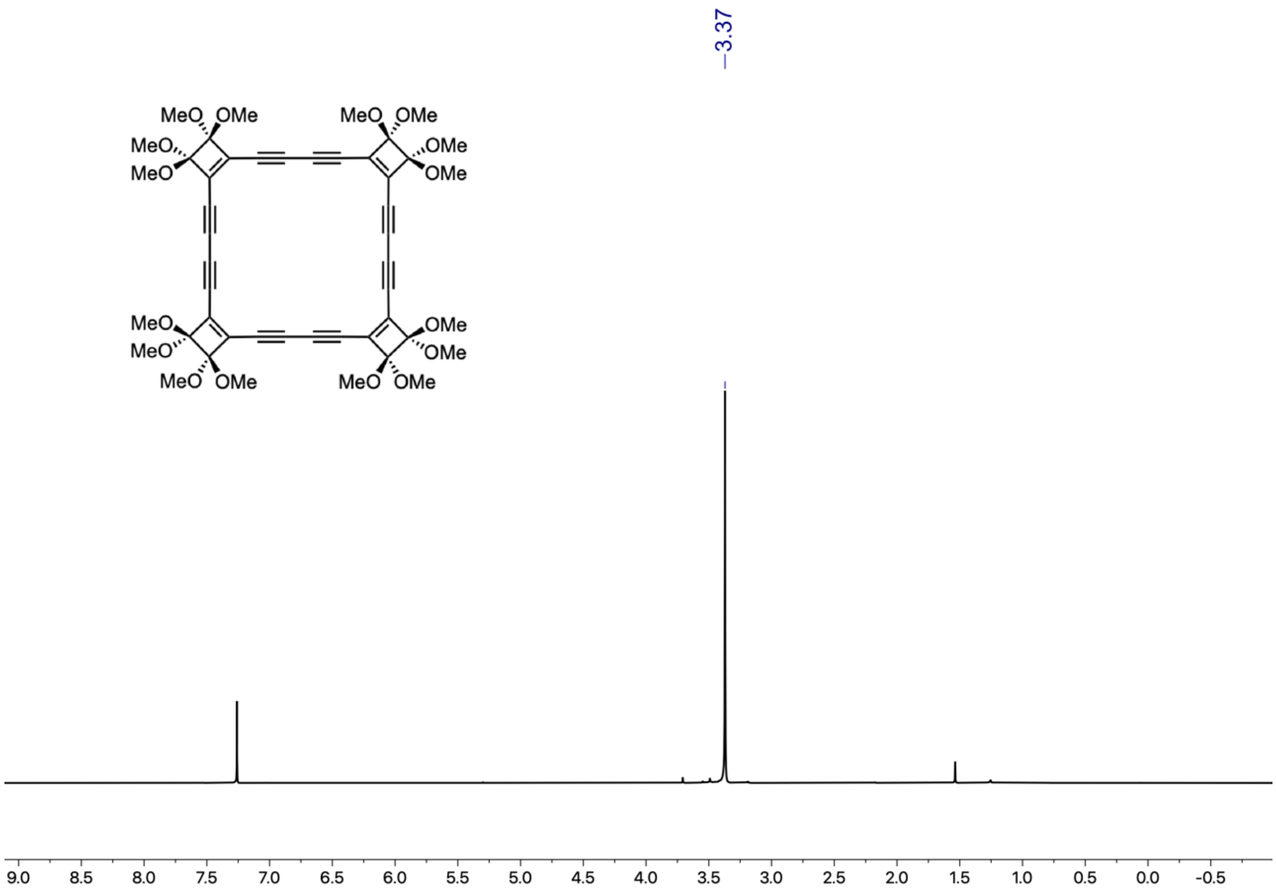

Fig. S50. ^{1}H NMR (400 MHz) spectrum of compound **S11**in $CDCl_3$.

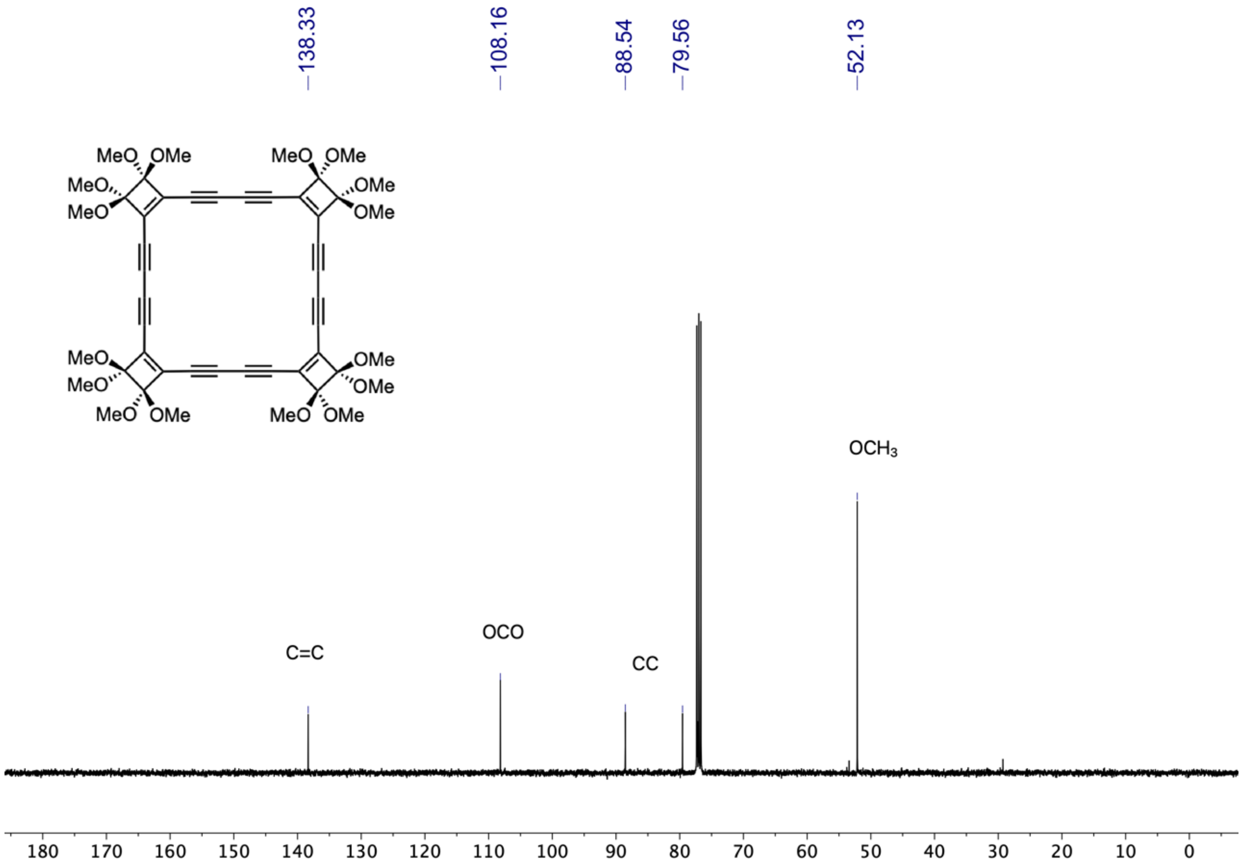

Fig. S51. ^{13}C NMR (101 MHz) spectrum of compound **S11** in $CDCl_3$.

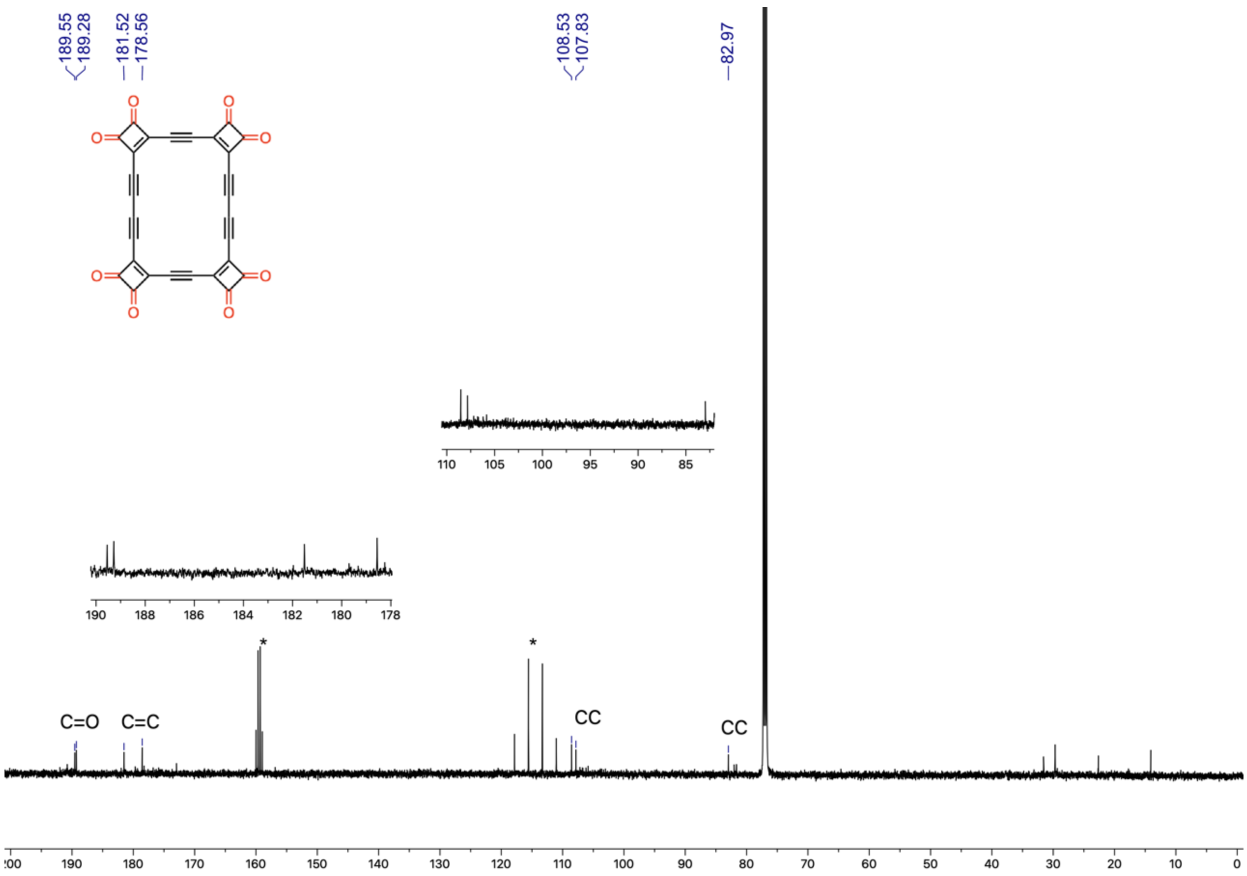

Fig. S52. ^{13}C NMR (126 MHz, 6500 scans) spectrum of compound **S13** in $CDCl_3$. *Denotes the residual TFA solvent.

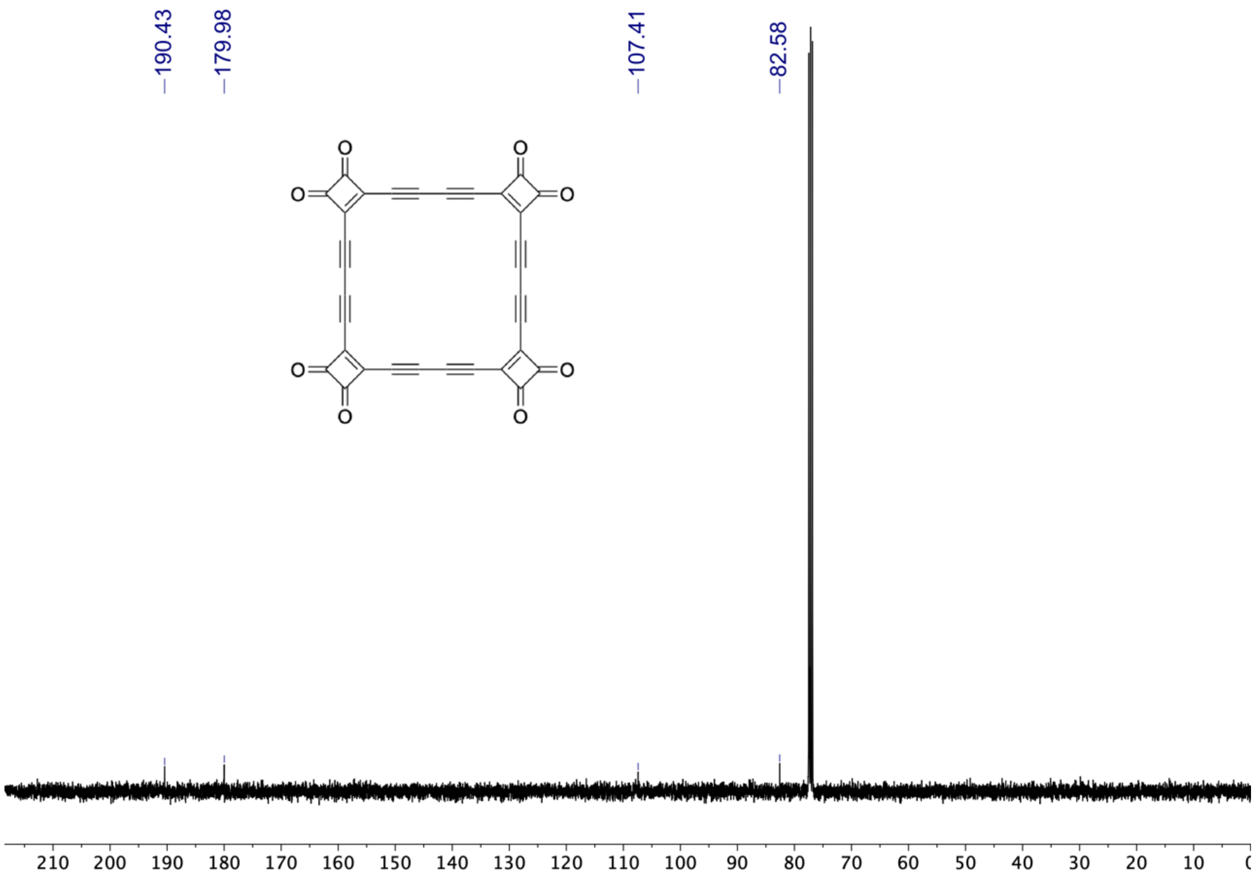

Fig. S53. ^{13}C NMR (101 MHz) spectrum of compound **4S14** in $CDCl_3$.

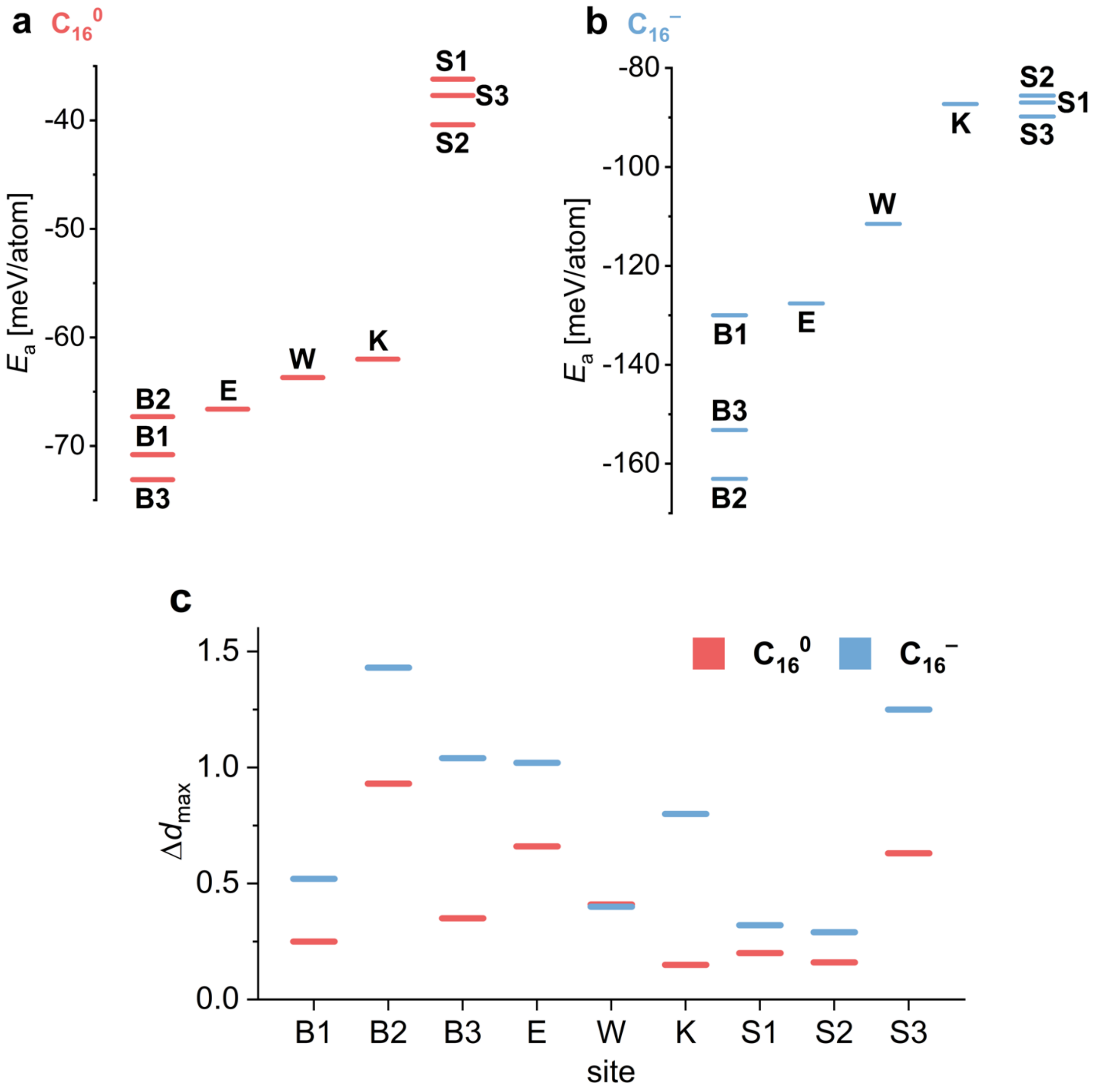

Fig. S54. Adsorption energies E_a of neutral C_{16} (a) and the C_{16} anion (b) for various sites on the (100) NaCl surface. See Table S1 for details. (c) Maximum difference in the ring diameter ($\Delta d_{max} = d_{max} - d_{min}$) for optimized geometries of neutral C_{16} (orange) and its anion (blue) at different sites, in Å.

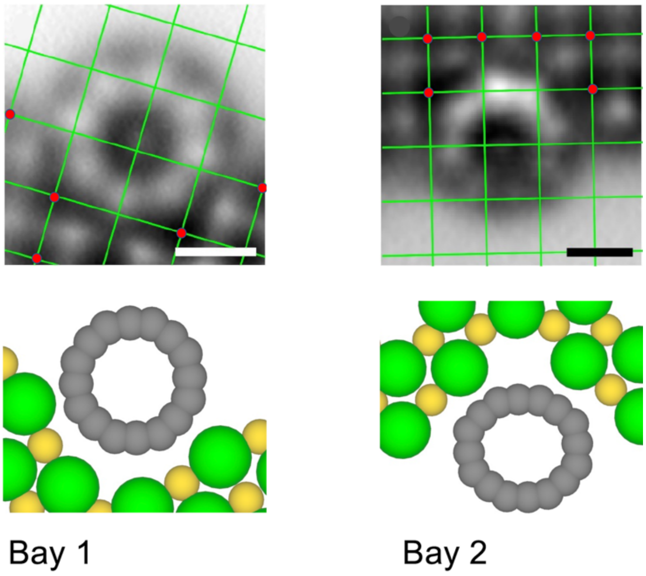

Fig. S55. Two examples of neutral C_{16} molecules at a bay of a NaCl island . Top: Experimental adsorption sites for C_{16}^{0} on bilayer NaCl on Cu(111). Crossings (and red dots) corresponding to sites of Na^{+} ions in the 3rd layer, from Fig. S14a,c (scale bars: 5 Å). Bottom: Optimized geometries of C_{16}^{0} on reconstructed NaCl surfaces with bay 1 and bay 2 geometry, from Table S1.

C. Supplementary Tables

Table S1. Optimized geometries of C_{16}^{0} and C_{16}^{-} on a reconstructed thin NaCl surface and at different third layer step edges, with adsorption energies (E_a, in meV per carbon atom), range of BAA values (in degrees), the average value of BLA and its standard deviation (in Å, with the latter in parentheses), and the difference between the largest and smallest ring diameter (Δd_{max}, in Å), as calculated by PBE-D3BJ. Na ions in yellow, Cl ions in green and C atoms in gray. For the adsorption sites near third layer step edges, the NaCl ions of the layers below the molecules are not plotted for clarity. Comparison of the molecular adsorption site at edge (E) and kink (K) to the experiment shown in Fig. S16, indicates that in that experiment the molecule was adsorbed at an step edge with edge geometry (E).

	**neutral**		
**vacuum**	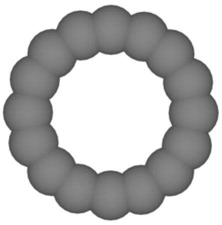		
BAA (°)	0–2		
BLA (Å)	0.10 (± 0.00)		
Δd_{max} (Å)	0.01		
	**S1 (Na^+)**	**S2 (Cl^-)**	**S3 (bridge)**
**defect-free Surface**	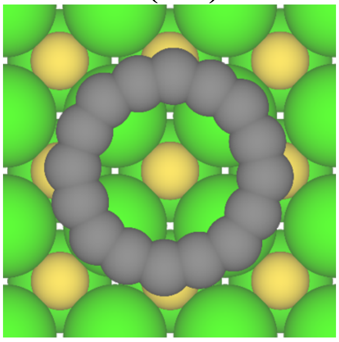	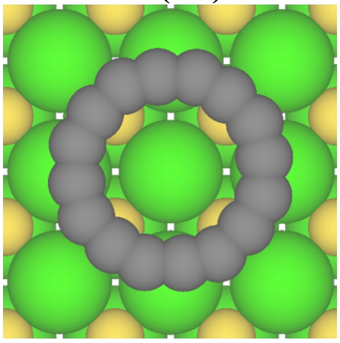	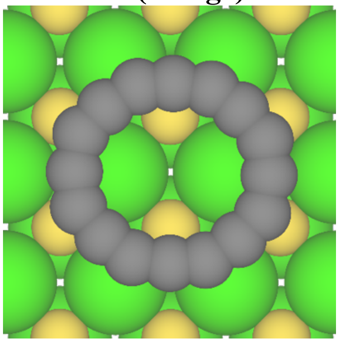
E_a (meV)	–36.2	–40.4	–37.7
BAA (°)	15	11–12	14–18
BLA (Å)	0.10 (± 0.00)	0.10 (± 0.00)	0.10 (± 0.00)
Δd_{max} (Å)	0.20	0.16	0.63
	**B1**	**B2**	**B3**
**bay**	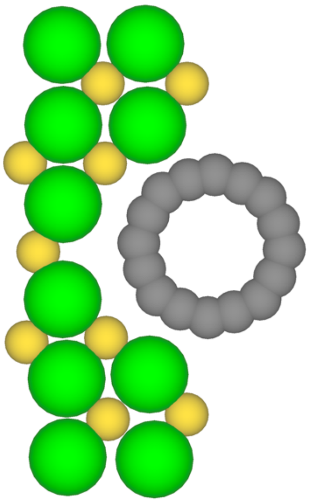	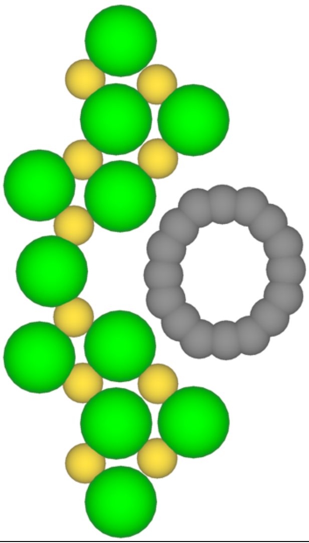	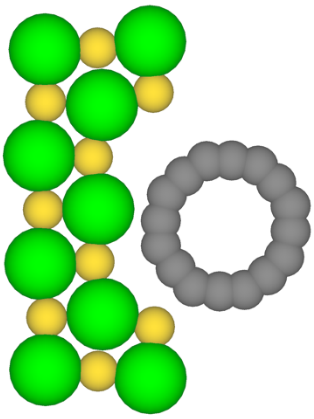

E_a (meV)	–70.8	–67.3	–73.1
BAA (°)	16–18	12–25	16–28
BLA (Å)	0.10 (± 0.00)	0.09 (± 0.00)	0.09 (± 0.01)
Δd_{max} (Å)	0.25	0.93	0.35
**neutral (cont.)**			
	**E**	**K**	**W**
**Edge, Kink, Wall**	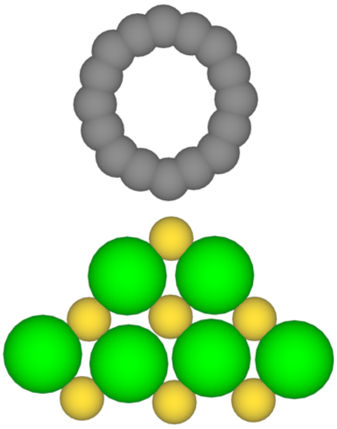	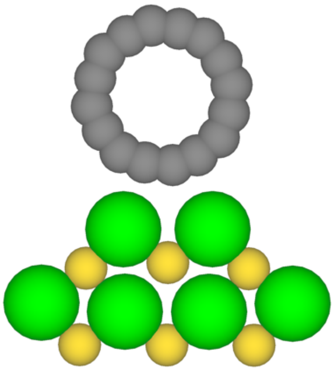	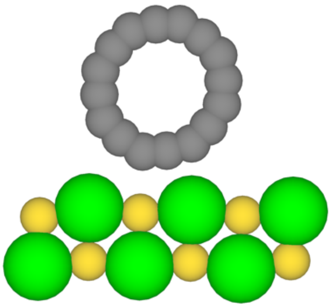
E_a (meV)	–66.6	–62.0	–63.7
BAA (°)	17–33	9–17	15–21
BLA (Å)	0.08 (± 0.01)	0.10 (± 0.00)	0.09 (± 0.00)
Δd_{max} (Å)	0.66	0.15	0.41
**anion**			
**vacuum**	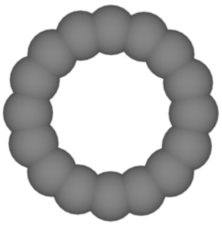		
BAA (°)	17–33		
BLA (Å)	0.08 (± 0.00)		
Δd_{max} (Å)	0.1		
	**S1 (Na^+)**	**S2 (Cl^-)**	**S3 (bridge)**
**defect-free Surface**	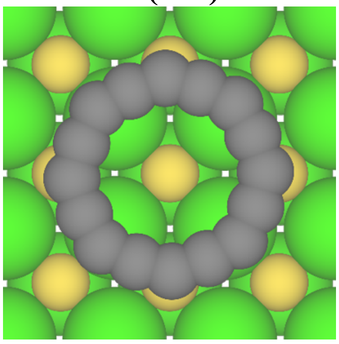	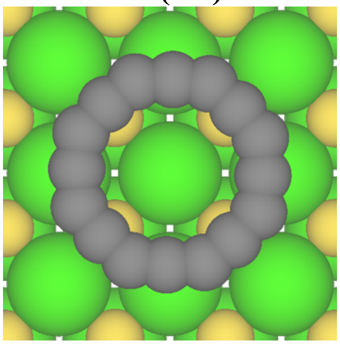	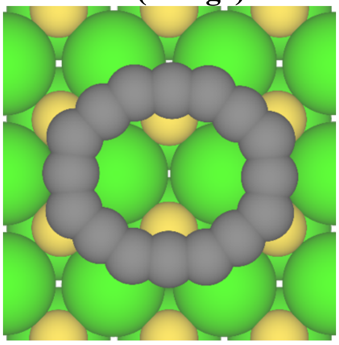
E_a (meV)	–87.0	–85.6	–89.8
BAA (°)	25	23–24	18–33
BLA (Å)	0.05 (± 0.00)	0.05 (± 0.00)	0.05 (± 0.01)
Δd_{max} (Å)	0.32	0.29	1.25

		**anion (cont.)**	
	**B1**	**B2**	**B3**
**bay**	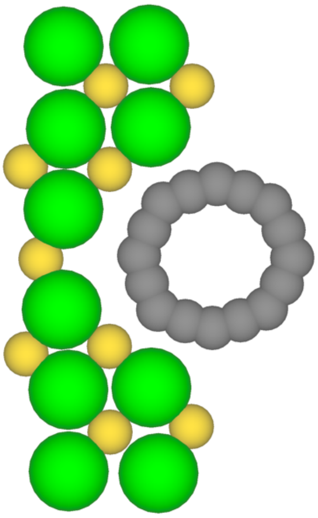	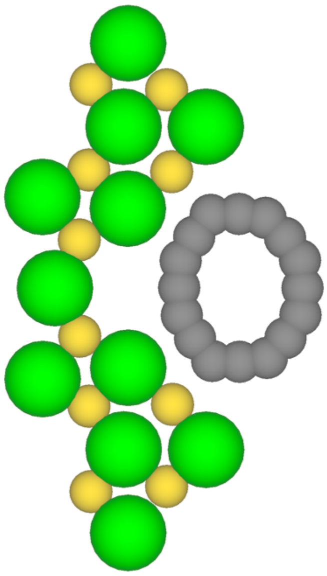	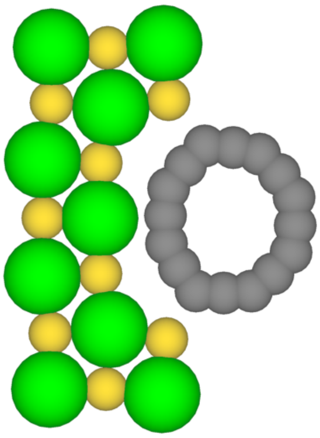
E_a (meV)	–130.0	–163.03	–153.2
BAA (°)	20–29	18–42	19–43
BLA (Å)	0.04 (± 0.02)	0.04 (± 0.01)	0.04 (± 0.02)
Δd_{max} (Å)	0.52	1.43	1.04
	**E**	**K**	**W**
**Edge, Kink, Wall**	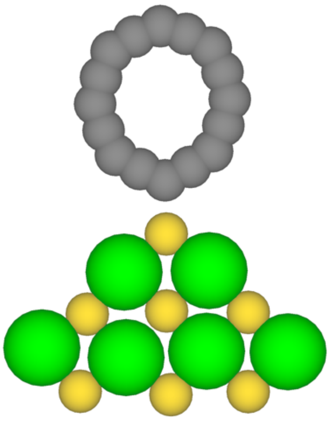	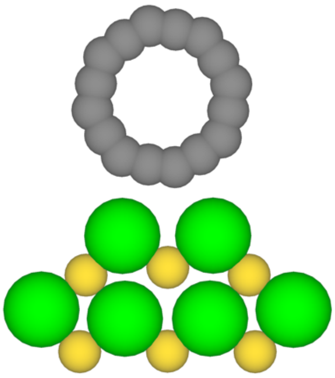	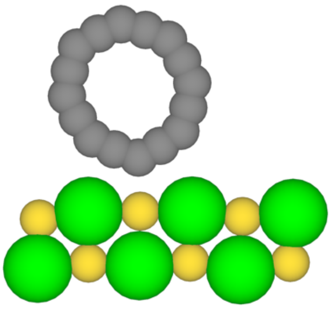
E_a (meV)	–127.6	–87.3	–111.5
BAA (°)	24–47	23–28	19–39
BLA (Å)	0.03 (± 0.01)	0.05 (± 0.00)	0.04 (± 0.01)
Δd_{max} (Å)	1.02	0.40	0.80

Table S2. Kelvin probe force spectroscopy (KPFS) on C_{16} shown in Fig. 3j–m and Fig. S8. Local contact potential difference (LCPD) values obtained by KPFS. The LCPD values recorded on top the molecule (2nd column) are similar to the values on bare bilayer NaCl (3rd column, recorded at a lateral distance of 50 Å from the molecule), i.e., they differ less than 50 mV. There is little change of LCPD values when approaching the tip towards the sample across a *z*-range of 2.5 Å, and the change is very similar above the C_{16} and the reference measurement on the bare surface. These findings indicate the circular cyclo[16]carbon, shown in Fig. 3j-m and Fig. S9, is neutral. Note that for a negatively charged adsorbate a shift of the LCPD towards more positive values upon approaching the tip would be expected, in contrast to the observed direction, that is to more negative LCPD values upon tip approach (cf. Table S3). At this particular adsorption site, we found the molecule to be only stable in the neutral charge state, which is probably related to the local electric field stemming from the 3rd layer NaCl island. That is, at this adsorption site the molecule could not be switched to the negative charge state. Imaging at elevated bias resulted in a dislocation of the molecule away from this adsorption site.

	position	
Tip-height offset [Å]	LCPD above C_{16} [V]	LCPD above bare bilayer NaCl/Cu(111) [V]
3.0	0.007	0.052
2.0	0.005	0.043
1.5	-0.007	0.026
1.0	-0.014	0.022
0.5	-0.014	0.021

Table S3. Kelvin probe force spectroscopy (KPFS) on C_{16} shown in Fig. 3f, g. Local contact potential difference (LCPD) values obtained by KPFS. The LCPD values recorded on top C_{16}^0 (2nd column) are similar to the values on bare bilayer NaCl (4th column, recorded at a lateral distance of 35 Å from the molecule), i.e., they differ by less than 55 mV. There is little change of LCPD values when approaching the tip towards the bare NaCl/Cu(111) sample across a *z*-range of 2.0 Å and the change is very similar above C_{16}^0. In contrast, the LCPD values recorded above C_{16}^- (3rd column) differ towards larger values from the reference values on bare bilayer NaCl. In addition, the LCPD values increase with the tip approaching the sample.

	Lateral tip position		
Tip-height offset [Å]	LCPD above C_{16}^0 [V]	LCPD above C_{16}^- [V]	LCPD above bare bilayer NaCl/Cu(111) [V]
3.0	0.048	0.183	0.072
2.0	0.038	0.190	0.066
1.5	0.013	0.243	0.066
1.0	0.012	0.257	0.042

Table S4. Calculated electron affinities (EA), ionization potentials (IP), vertical excitation energy (to S1) state in eV, ring radii (r_{ring}), and bond lengths (a_{long}, a_{short}) in Å for C_{16}^0 (gas phase).

	CASSCF	NEVPT2	CASPT2	EOM-CCSD	DSD-PBEP86	ωB97XD
EA	1.41	2.06	2.08	2.19	2.29	2.09
IP	7.24	8.76	8.11	8.27	8.03	8.12
S1	1.97	2.01	2.08	2.06	2.17	1.94
r_{ring}	3.27	3.33			3.32	3.30
a_{long}	1.33	1.36			1.35	1.36
a_{short}	1.22	1.24			1.23	1.21

Table S5. Bond lengths (a_{long} and a_{short}, in Å), bond angles (θ_1 and θ_2, in degrees), and point groups of optimized geometries of C_{16} and its ions (gas phase), obtained using the ωB97XD/def2-TZVP level of theory.

molecule	a_{long}	a_{short}	θ_1	θ_2	point group
C_{16}^+	1.34	1.22	157.5		D_{8h}
C_{16}^0	1.36	1.21	157.5		D_{8h}
C_{16}^-	1.34	1.23	157.5		[a]D_{8h}
C_{16}^-	1.33	1.25	170.6	144.4	C_{8h}
C_{16}^{2-}	1.29		174.4	140.6	D_{8h}

[a] A first-order saddle point on the potential energy surface. The associated imaginary frequency is shown in Fig. S29, and the energy relative to the C_{8h} minimum is 68 meV.

Section D. **List of Deposited Datasets**

The computational outputs listed here are available from the Zenodo public repository (https://zenodo.org/record/8226451 and https://doi.org/10.5281/zenodo.8226451):

a. xyz format:
   1. Optimised geometry of neutral C_{16}, NEVPT2: c16_0_NEVPT2.xyz (Section A8, Fig. S23)
   2. Optimised geometry of neutral C_{16}, CASSCF: c16_0_CASSCF.xyz (Section A8)
   3. Optimised geometry of neutral C_{16}, DSD-PBEP86: c16_DSD-PBEP86.xyz (Section A8, Fig. S19)
   4. Optimised geometry of C_{16} in different charge states, ωB97XD:
      i. c16_+2_wb97xd_opt_freq.xyz (Section A8)
      ii. c16_+1_wb97xd_opt_freq.xyz (Section A8, Fig. S22)
      iii. c16_+0_wb97xd_opt_freq.xyz (Main text Fig. 4k, Section A8, Fig. S19)
      iv. c16_-1_wb97xd_opt_freq.xyz (Main text Fig. 4l, Section A8, Fig. S20)
      v. c16_-2_wb97xd_opt_freq.xyz (Section A8, Fig. S21)

b. Gaussian output (log) format, ωB97XD optimisations and frequency runs:
   1. c16_+2_wb97xd_opt_freq.log
   2. c16_+1_wb97xd_opt_freq.log (Section A8, Fig. S22)
   3. c16_+0_wb97xd_opt_freq.log (Section A8, Fig. S19)
   4. c16_-1_wb97xd_opt_freq.log (Section A8, Fig. S20)
   5. c16_-2_wb97xd_opt_freq.log (Section A8, Fig. S21)

c. Gaussian formatted checkpoint file:
   1. Doubly aromatic <2200| state: c16_0_wb97xd_sp_2200.fchk (Main text Fig. 1, Section A8)

d. VASP output (POSCAR) files, pristine surface:
   1. CONTCAR_1_neutral (Section A9, Table S1, C_{16}^{0}, S1 (Na^{+}))
   2. CONTCAR_2_neutral (Main text Fig. 3h, Section A9, Table S1, C_{16}^{0}, S2 (Cl^{-}))
   3. CONTCAR_3_neutral (Section A9, Table S1, C_{16}^{0}, S3 (bridge))
   4. CONTCAR_1_anion (Section A9, Table S1, C_{16}^{-}, S1 (Na^{+}))
   5. CONTCAR_2_anion (Section A9, Table S1, C_{16}^{-}, S2 (Cl^{-}))
   6. CONTCAR_3_anion (Main text Fig. 3i, Section A9, Table S1, C_{16}^{-}, S3 (bridge))

e. VASP output (POSCAR) files, defects:
   1. CONTCAR_neutral_B1 (Section A9, Table S1, C_{16}^{0}, B1)
   2. CONTCAR_neutral_B2 (Section A9, Table S1, C_{16}^{0}, B2)
   3. CONTCAR_neutral_B3 (Section A9, Table S1, C_{16}^{0}, B3)
   4. CONTCAR_neutral_E (Section A9, Table S1, C_{16}^{0}, E)
   5. CONTCAR_neutral_K (Section A9, Table S1, C_{16}^{0}, K)
   6. CONTCAR_neutral_W (Section A9, Table S1, C_{16}^{0}, W)
   7. CONTCAR_anion_B1 (Section A9, Table S1, C_{16}^{-}, B1)
   8. CONTCAR_ anion _B2 (Section A9, Table S1, C_{16}^{-}, B2)
   9. CONTCAR_ anion _B3 (Section A9, Table S1, C_{16}^{-}, B3)
   10. CONTCAR_ anion _E (Section A9, Table S1, C_{16}^{-}, E)
   11. CONTCAR_ anion _K (Section A9, Table S1, C_{16}^{-}, K)
   12. CONTCAR_ anion _W (Section A9, Table S1, C_{16}^{-}, W)

The dataset consists of 32 files in total.

Section E. **Supplementary References**

1. Giessibl, F. J. High-speed force sensor for force microscopy and profilometry utilizing a quartz tuning fork. *Appl. Phys. Lett.* **73**, 3956–3958 (1998).
2. Albrecht, T. R., Grütter, P., Horne, D. & Rugar, D. Frequency modulation detection using high-Q cantilevers for enhanced force microscope sensitivity. *J. Appl. Phys.* **69**, 668–673 (1991).
3. Palatinus, L. & Chapuis, G. *SUPERFLIP* – a computer program for the solution of crystal structures by charge flipping in arbitrary dimensions. *J. Appl. Cryst.* **40**, 786–790 (2007).
4. Parois, P., Cooper, R. I. & Thompson, A. L. Crystal structures of increasingly large molecules: meeting the challenges with CRYSTALS software. *Chem. Cent. J.* **9**, 30 (2015).
5. Cooper, R. I., Thompson, A. L. & Watkin, D. J. *CRYSTALS* enhancements: dealing with hydrogen atoms in refinement. *J. Appl. Cryst.* **43**, 1100–1107 (2010).
6. Neese, F., Wennmohs, F., Becker, U. & Riplinger, C. The ORCA quantum chemistry program package. *J. Chem. Phys.* **152**, 224108 (2020).
7. Fdez. Galván, I. et al. OpenMolcas: From source code to insight. *J. Chem. Theory Comput.* **15**, 5925–5964 (2019).
8. Riplinger, C. & Neese, F. An efficient and near linear scaling pair natural orbital based local coupled cluster method. *J. Chem. Phys.* **138**, 034106 (2013).
9. Frisch, M. J. et al. Gaussian16. (Wallingford, CT, 2016).
10. Weigend, F. & Ahlrichs, R. Balanced basis sets of split valence, triple zeta valence and quadruple zeta valence quality for H to Rn: Design and assessment of accuracy. *Phys. Chem. Chem. Phys.* **7**, 3297–3305 (2005).
11. Widmark, P.-O., Malmqvist, P.-K. & Roos, B. O. Density matrix averaged atomic natural orbital (ANO) basis sets for correlated molecular wave functions. *Theor. Chim. Acta* **77**, 291–306 (1990).
12. Perdew, J. P., Burke, K. & Ernzerhof, M. Generalized gradient approximation made simple. *Phys. Rev. Lett.* **77**, 3865–3868 (1996).
13. Grimme, S., Antony, J., Ehrlich, S. & Krieg, H. A consistent and accurate ab initio parametrization of density functional dispersion correction (DFT-D) for the 94 elements H-Pu. *J. Chem. Phys.* **132**, 154104 (2010).
14. Kresse, G. & Hafner, J. *Ab initio* molecular dynamics for liquid metals. *Phys. Rev. B: Condens. Matter Mater. Phys.* **47**, 558–561 (1993).
15. Kresse, G. & Hafner, J. *Ab initio* molecular-dynamics simulation of the liquid–metal–amorphous-semiconductor transition in germanium. *Phys. Rev. B: Condens. Matter Mater. Phys.* **49**, 14251–14269 (1994).
16. Gross, L. et al. Investigating atomic contrast in atomic force microscopy and Kelvin probe force microscopy on ionic systems using functionalized tips. *Phys. Rev. B* **90**, 155455 (2014).
17. Barkoutsos, P. K. et al. Quantum algorithms for electronic structure calculations: particle-hole Hamiltonian and optimized wave-function expansions. *Phys. Rev. A* **98**, 022322 (2018).
18. Kaiser, K. et al. An sp-hybridized molecular carbon allotrope, cyclo[18]carbon. *Science* **365**, 1299–1301 (2019).

19. Scriven, L. M. et al. Synthesis of cyclo[18]carbon *via* debromination of $C_{18}Br_6$. *J. Am. Chem. Soc.* **142**, 12921–12924 (2020).

20. Rubin, Y., Kahr, M., Knobler, C. B., Diederich, F. & Wilkins, C. L. *J. Am. Chem. Soc.* **113**, 495–500 (1991).

21. Baryshnikov, G. V. et al. Aromaticity of even-number cyclo[*n*]carbons (n = 6–100). *J. Phys. Chem. A* **124**, 10849–10855 (2020).

22. G. V. Baryshnikov, R. R. Valiev, A. V. Kuklin, D. Sundholm, H. Ågren, Cyclo[18]carbon: insight into electronic structure, aromaticity, and surface coupling. *J. Phys. Chem. Lett.* **10**, 6701–6705 (2019).

23. Schapiro, I., Sivalingam, K. & Neese, F. Assessment of n-electron valence state perturbation theory for vertical excitation energies. *J. Chem. Theory Comp.* **9**, 3567–3580 (2013).

24. Chai, J.-D. & Head-Gordon, M. Long-range corrected hybrid density functionals with damped atom–atom dispersion corrections. *Phys. Chem. Chem. Phys.* **10**, 6615–6620 (2008).

25. Baryshnikov, G. V. et al. Odd-number cyclo[*n*]carbons sustaining alternating aromaticity. *J. Phys. Chem. A* **126**, 2445–2452 (2022).

26. Kozuch, S. & Martin, J. M. L. DSD-PBEP86: in search of the best double-hybrid DFT with spin-component scaled MP2 and dispersion corrections. *Phys. Chem. Chem. Phys.* **13**, 20104–20107 (2011).

27. Monaco, G., Summa, F. F. & Zanasi, R. Program package for the calculation of origin-independent electron current density and derived magnetic properties in molecular systems. *J. Chem. Inf. Model.* **61**, 270−283 (2021).

28. Baryshnikov, G. V., Valiev, R. R., Kuklin, A. V., Sundholm, D. & Ågren, H. Cyclo[18]carbon: insight into electronic structure, aromaticity, and surface coupling. *J. Phys. Chem. Lett.* **10**, 6701−6705 (2019).

29. NBO Version 3.1, E. D. Glendening, A. E. Reed, J. E. Carpenter, F. Weinhold.

30. Anderson, H. L., Patrick, C. W., Scriven, L. M. & Woltering, S. L. A short history of cyclocarbons. *Bull. Chem. Soc. Jpn.* **94**, 798–811 (2021).

31. Remya, K. & Suresh, C. H. Carbon rings: a DFT study on geometry, aromaticity, intermolecular carbon–carbon interactions and stability. *RSC Adv.* **6**, 44261–44271 (2016).

32. Repp, J., Meyer, G. & Rieder K.-H. Snell's Law for surface electrons: Refraction of an electron gas imaged in real space. *Phys. Rev. Lett.* 92, 36803 (2004)

33. Steiner, E. & Fowler, P. W. On the orbital analysis of magnetic properties. *Phys. Chem. Chem. Phys.* **6**, 261–272 (2004).
